# A novel methodology for the efficient synthesis of 3-monohalooxindoles by acidolysis of 3-phosphate-substituted oxindoles with haloid acids

**DOI:** 10.3762/bjoc.17.150

**Published:** 2021-09-07

**Authors:** Li Liu, Yue Li, Tiao Huang, Dulin Kong, Mingshu Wu

**Affiliations:** 1 Key Laboratory of Tropical Medicinal Plant Chemistry of the Ministry of Education, College of Chemistry & Chemical Engineering, Hainan Normal University, Haikou 571158, Hainan Province, P. R. China; 2 School of Pharmaceutical Sciences, Hainan Medical University, Haikou 571199, Hainan Province, P. R. China

**Keywords:** acidolysis, haloid acids, isatin, 3-monohalooxindole, 3-phosphate-substituted oxindoles

## Abstract

A novel method for the synthesis of 3-monohalooxindoles by acidolysis of isatin-derived 3-phosphate-substituted oxindoles with haloid acids was developed. This synthetic strategy involved the preparation of 3-phosphate-substituted oxindole intermediates and S_N_1 reactions with haloid acids. This new procedure features mild reaction conditions, simple operation, good yield, readily available and inexpensive starting materials, and gram-scalability.

## Introduction

**Figure 1:**
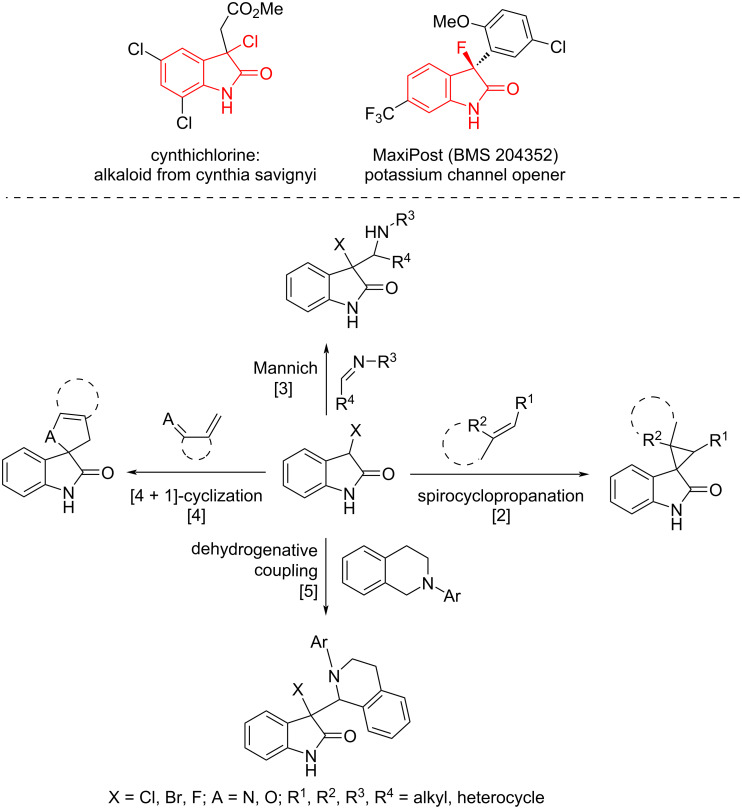
Representation of bioactive molecules and applications.

**Scheme 1:**
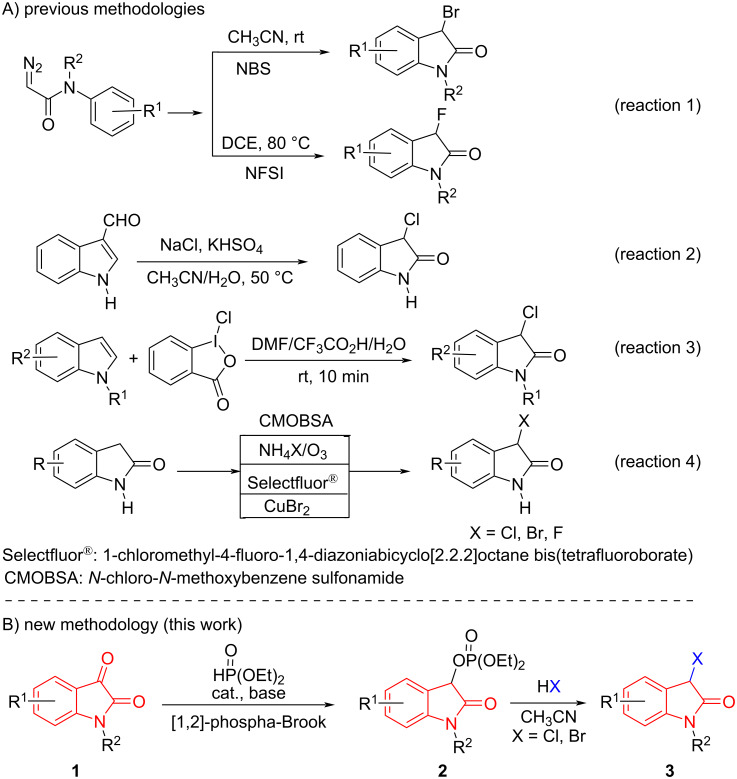
Synthetic methodologies for 3-monohalooxindoles.

3-Monohalooxindole heterocycles are not only present as a characteristic structural motif in numerous biological and medicinal molecules [1–2] but also possess dual nucleophilic and electrophilic character at the C-3 position. Owing to the dual nature at the C-3 position, 3-monohalooxindoles have emerged as a class of versatile building blocks for the construction of various 3,3-disubstituted oxindole and spirooxindole derivatives, such as spirocyclopropaneoxindoles [3–11], 3-β-amino-substituted 3-halooxindoles [12–14], five-membered-ring-based spirooxindoles [15–18] and 3-alkyl-substituted 3-fluorooxindoles (Figure 1) [19]. Despite the importance of 3-monohalooxindoles in organic synthesis and medicinal chemistry, only a few methods for the synthesis of these 3-monohalooxindoles have been reported. Recently, Xu and co-workers disclosed the application of *N*-fluorobenzenesulfonimide (NFSI) and NBS (*N*-bromosuccinimide), respectively, as the halogen sources, with diazoacetamide under catalyst-free conditions via a carbene pathway, which constructed 3-fluorooxindoles and 3-bromooxindoles (Scheme 1, reaction 1) [20–21]. Then, the Prathima group established an expedient approach for the direct oxidative chlorination of indole-3-carboxaldehyde to 3-monochlorooxindoles using a combination of NaCl and oxone as the chlorine source and oxidant in a CH_3_CN/H_2_O 1:1 system (Scheme 1, reaction 2) [22].

Nearly at the same time, Yu and co-workers reported controllable mono- and dichlorooxidation of indoles with hypervalent iodine species in DMF/CF_3_CO_2_H/H_2_O at room temperature, which generated 3,3-dichlorooxindoles and 3-monochlorooxindoles, respectively (Scheme 1, reaction 3) [23]. Apart from these methods, most traditional approaches to 3-monohalooxindoles involve the direct halogenation of oxindoles with various reactive halogenating reagents, including *N*-chloro-*N*-methoxybenzenesulfonamide [24–25], ammonium halides/oxone [13], Selectfluor^®^ [26–27], and CuBr_2_ (Scheme 1, reaction 4) [15]. However, these protocols each have a certain scope and limitations. The development of methods that provide efficient access to a wide range of 3-monohalooxindoles from readily available and inexpensive starting materials is still a formidable challenge because the synthesis should be practical for large-scale industrial use and feature reasonably priced products. Thus, further work is needed to develop a novel strategy for an efficient synthesis of such a versatile synthon.

On the other hand, diethyl (2-oxoindolin-3-yl) phosphates **2** were easily prepared by the base-catalyzed phospha-Brook rearrangement of isatins **1** with diethyl phosphite [28–29]. This compound has a remarkable structural feature: the phosphate moiety is located at the benzylic position as well as at the position α to an amide group, which makes it a good leaving group for the design and development of new reactions. Accordingly, diethyl (2-oxoindolin-3-yl) phosphates **2** have been used recently as precursors in Friedel–Crafts reactions of arenes [30–31] and cross-coupling reactions of arylboronic reagents [32]. However, the direct S_N_1 reaction of such isatin-derived 3-phosphate-substituted oxindoles by halide ions as nucleophiles has not been developed yet and remains an unsolved challenge in chemistry.

In order to achieve this goal, and on the basis of our previous experiences in the functionalization of oxindoles [33–34], we herein designed a nucleophilic substitution method of an isatin-derived 3-phosphate-substituted oxindole with haloid acids, leading to 3-monohalooxindoles (Scheme 1).

## Results and Discussion

During the exploratory study of this work, we chose concentrated hydrochloric acid (36%) as the readily available chlorinating reagent to screen the reaction conditions, and we carried out our initial synthetic reaction with diethyl (2-oxoindolin-3-yl) phosphate (**2a**) under solvent-free and catalyst-free conditions at room temperature (Table 1, entry 1). To our delight, the desired product **3a** was obtained in 19% yield. To further improve the yield, we firstly probed the solvent effect using methanol, THF, toluene, ClCH_2_CH_2_Cl, 1,4-dioxane, chloroform, dichloromethane, and acetonitrile (Table 1, entries 2–9). The results indicated that the solvent has a meaningful impact on the efficiency of the reaction. Among the tested solvents, CH_3_CN was the best choice for the process (Table 1, entry 9). In this instance, a high yield (89%) was achieved. Then, in the presence of the best solvent CH_3_CN, we tested the effect of the temperature on the reaction. Lowering the reaction temperature to 0 °C and 10 °C, respectively, led to a failure of the reaction (Table 1, entries 10 and 11), while elevating the reaction temperature from 40 °C to 50 °C resulted in the highest yield (92%, Table 1, entry 13). However, further increasing the reaction temperature to 60 °C led to a sharp decrease of the yield (Table 1, entry 14). Therefore, 50 °C was set as the most suitable reaction temperature. Furthermore, we evaluated the effect of the reaction time on the acidolysis reaction (Table 1, entry 15). Prolonging the reaction time to 8 h did not improve the yield (Table 1, entry 15), whereas shortening the reaction time to 5 h reduced the yield (Table 1, entry 16), and thus revealing 6 h to be best reaction time. Finally, the effect of Lewis acid catalysts, such as ZnCl_2_, FeCl_3_, AlCl_3_, Cu (CF_3_SO_3_)_2_, and CuCl_2_, on the reaction was also examined, but no significant improvement in the yield was found (Table 1, entries 17–21). Considering all of the reaction parameters, the optimal reaction conditions were chosen as shown in Table 1, entry 13.

**Table 1:** Optimization studies.^a^

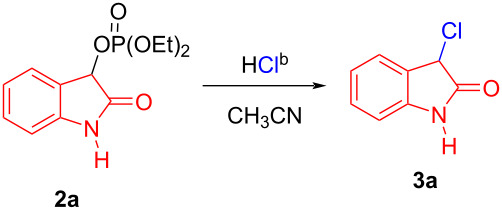					

entry	catalyst	solvent	*T* (°C)	*t* (h)	yield^c^ (%)

1	—	—	rt	6	19
2	—	CH_3_OH	rt	6	49
3	—	THF	rt	6	trace
4	—	toluene	rt	6	68
5	—	ClCH_2_CH_2_Cl	rt	6	68
6	—	1,4-dioxane	rt	6	28
7	—	CHCl_3_	rt	6	82
8	—	CH_2_Cl_2_	rt	6	67
9	—	CH_3_CN	rt	6	89
10	—	CH_3_CN	0	6	trace
11	—	CH_3_CN	10	6	38
12	—	CH_3_CN	40	6	88
13	—	CH_3_CN	50	6	92
14	—	CH_3_CN	60	6	86
15	—	CH_3_CN	50	8	92
16	—	CH_3_CN	50	5	85
17	ZnCl_2_	CH_3_CN	50	6	91
18	FeCl_3_	CH_3_CN	50	6	90
19	AlCl_3_	CH_3_CN	50	6	90
20	Cu(CF_3_SO_3_)_2_	CH_3_CN	50	6	58
21	CuCl	CH_3_CN	50	6	87

^a^Reaction conditions: **2a** (0.2 mmol), solvent (2mL). ^b^Concentrated hydrochloric acid (36%, 15 equiv). ^c^Isolated yield.

**Scheme 2:**
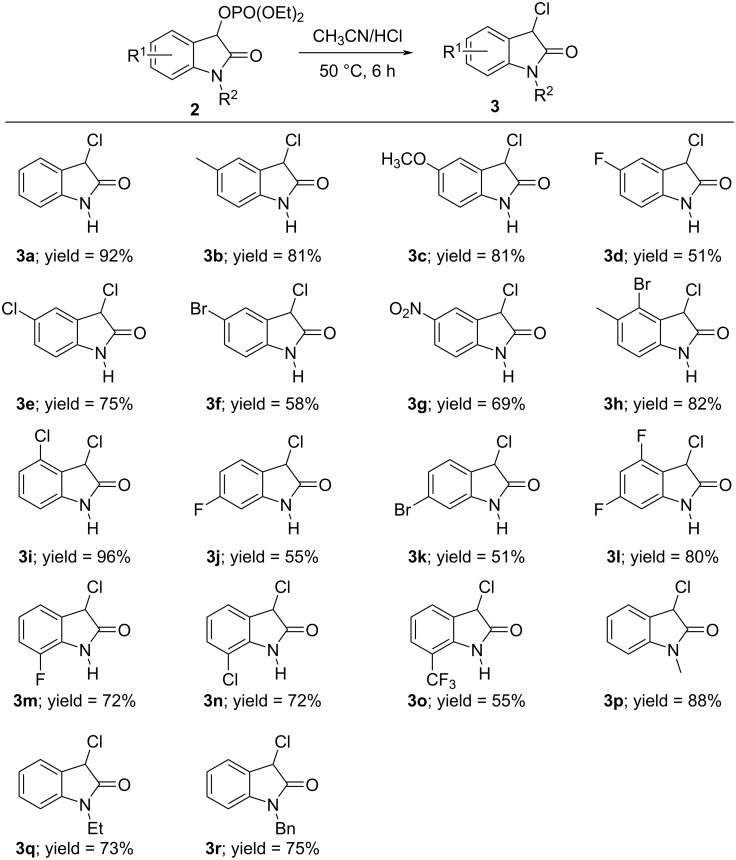
Substrate scope of the acidolysis of isatin-derived phosphates **2** with hydrochloric acid. Standard reaction conditions: **2a–r** (0.5 mmol), respectively, hydrochloric acid (7.5 mmol, 15 equiv), CH_3_CN (3 mL), 50 °C, 6 h. The isolated yields are given.

Once the optimization studies were concluded, we focused our attention on investigating the substrate scope and generality of this protocol. First, we examined the substrate scope of this transformation between hydrochloric acid (36%) with various substituted (2-oxoindolin-3-yl) phosphates **2**. As shown in Scheme 2, this reaction was applicable to a wide range of substrates, which generally offered the corresponding 3-monochlorooxindoles **3a–r** with a good yield (51–96%), regardless of the electronic nature and position of the substituents on the aromatic ring of **2**. In detail, (2-oxoindolin-3-yl) phosphates with an electron-donating methyl or methoxy substituent gave a better yield (see **3b** and **3c**) than starting materials with an electron-withdrawing group, such as a NO_2_, CF_3_, Cl, Br, or F substituent (see **3d**–**g**, **3j**, **3k**, and **3o**). Surprisingly, a phosphate possessing two electron-withdrawing substituents in the form of a 4,6-difluoro motif allowed to access the corresponding product **3l** in a higher yield. Notably, phosphate **2a** with no substitution on the aromatic ring and on the nitrogen atom showed a good reactivity, furnishing the corresponding product **3a** in the highest yield, possibly owing to not having steric hindrance. It appeared that the position of the residue R^1^ on the aryl ring exerted a pronounced effect on the reactivity. For instance, 4-bromo-, 4-chloro-, 4,6-difluoro-, 7-fluoro-, and 7-chloro-substituted phosphates afforded the corresponding products **3h**, **3i**, and **3l**–**n** in a higher yield than the 5-fluoro-, 5-bromo-, 6-fluoro-, and 6-bromo-substituted phosphates (see **3d**, **3f**, **3j**, and **3k**). In addition, N-protected (2-oxoindolin-3-yl) phosphates with R^2^ = Me, Et, and Bn, respectively, were also found to be suitable for the transformation and gave the respective products **3p**–**r** in good yield (73–88%, Scheme 2).

**Scheme 3:**
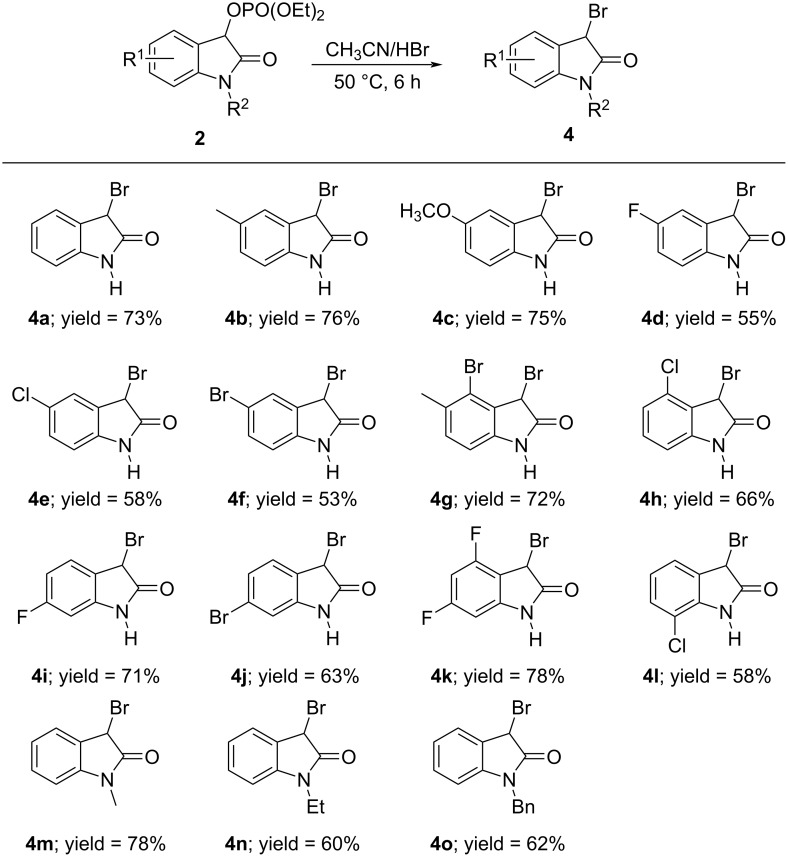
Substrate scope of the acidolysis of isatin-derived phosphates **2** with hydrobromic acid. Standard reaction conditions: **2a–2o** (0.5 mmol), hydrobromic acid (7.5 mmol, 15 equiv), CH_3_CN (3 mL), 50 °C, 6 h. The isolated yield is given.

In order to further extend the substrate scope, we tried using hydrobromic acid (40%) as a bromine source in the reaction, and the results are summarized in Scheme 3. Generally, the phosphate substrates **2** substituted with electron-donating substituents were more reactive than those with electron-withdrawing motifs, and thus gave a better result. For example, the (2-oxoindolin-3-yl) phosphate substrates with a methyl or methoxy group at the 5-position of the benzene ring could all react with hydrobromic acid in good yield, giving **4b**, **4c**, and **4g** in 72–76% yield. The position of the residue R^1^ on the phenyl ring of the (2-oxoindolin-3-yl) phosphate had an obvious effect on the reactivity. For example, (2-oxoindolin-3-yl) phosphates bearing a bromo or fluoro substituent in the 6-position all gave the corresponding products in a higher yield than the analogous precursors substituted in the 5-position (see **4i** and **4j** vs **4d** and **4f**). Moreover, there was seemingly no significant difference in the reactivity of the starting materials carrying a chloro substituent in the 4- and the 7-position, respectively, since the products **4h** and **4l** were obtained in a comparable yield of 66 and 58%, respectively. The substrate with no residue R^1^ on the phenyl ring produced the corresponding product **4a** in a higher yield than some the substituted substrates. In addition, N-protected (2-oxoindolin-3-yl) phosphate substrates could also deliver the products in good yield (see **4m–o**), even though bulkier N-protecting groups, i.e., benzyl and ethyl, slightly decreased the yields of the products (see **4n** and **4o**).

**Scheme 4:**
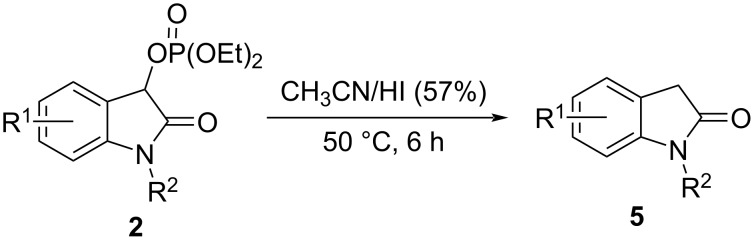
Reduction of the substrates **2** to the corresponding oxindoles **5**.

Regrettably, when a substrate **2** bearing a strong electron-withdrawing nitro or trifluoromethyl group on the phenyl ring was employed, the reaction gave very complex side products under the standard conditions, and almost no product was observed. In addition, we also tested hydroiodic and hydrofluoric acid as a halogenating reagent in the reaction, which did not provide any desired product. Interestingly, the (2-oxoindolin-3-yl) phosphate substrates could be directly reduced into oxindoles using hydroiodic acid (57%, Scheme 4).

To show the utility of this novel method, we performed the syntheses of **3a** from Scheme 2 on a 1 mol-scale. This larger-scale reaction smoothly took place to give the product **3a** in 95% yield under the standard conditions, which was similar to the result of the smaller-scale reaction, and column chromatography separation is not usually required. This outcome indicated that the transformation could be applicable for larger-scale syntheses of 3-monohalooxindoles products. In addition, the structure of all products **3** and **4** was unambiguously assigned by ^1^H and ^13^C NMR spectroscopy and HRMS. Especially the proton at the C-3-position of 3-monohalooxindoles gave diagnostic singlets (5.25–5.93 Hz) instead of double peaks due to the absence of coupling with the phosphorus atom in the ^1^H NMR experiment. This indicated that the methylene moiety adjacent to the phosphate group had been displaced by a halogen atom, which further implied that the halogenation reaction with haloid acids had occurred.

**Scheme 5:**
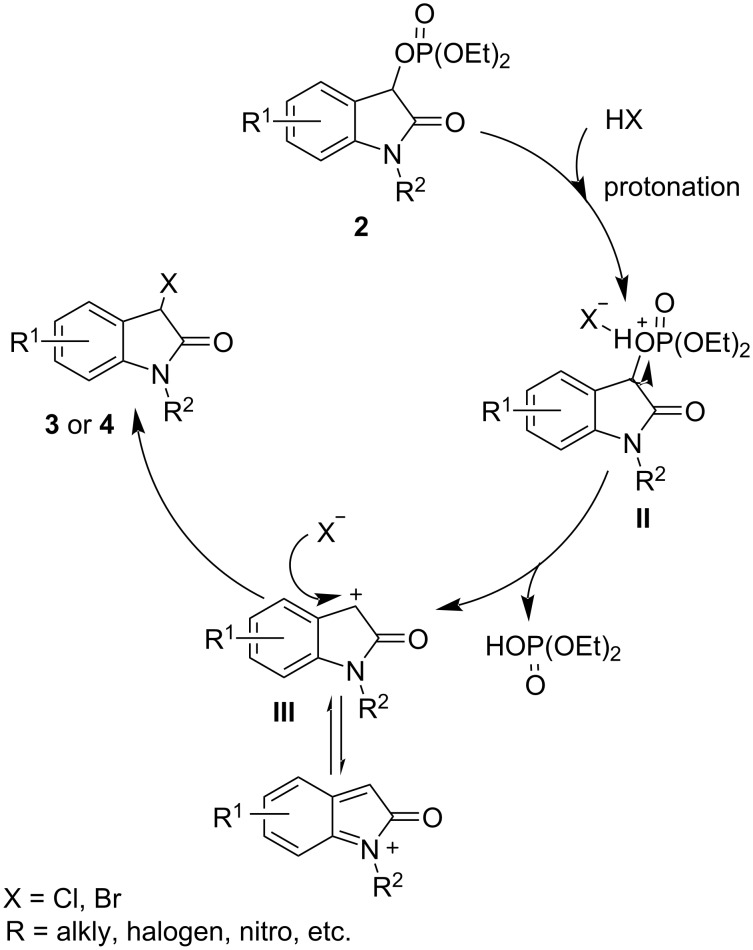
Plausible reaction mechanism.

On the basis of this study and the early related reports [30,35], an S_N_1 mechanism for this transformation is proposed as illustrated in Scheme 5. Initially, the C–O bond of the C-3 position of a diethyl (2-oxoindolin-3-yl) phosphate **2** is activated by protonation with a haloid acid. Subsequently the phosphate leaving group is eliminated to generate the carbocation intermediate **III**, which is then followed by rapid combination with a nucleophilic halide ion to form a 3-monohalooxindoles **3** or **4**.

## Conclusion

In summary, a new method for the synthesis of 3-monohalooxindoles via acidolysis of isatin-derived 3-phosphate-substituted oxindoles with haloid acids was developed. The present methodology involves the formation of an oxindole having a phosphate moiety at the C-3 position via the [1,2]-phospha-Brook rearrangement under Brønsted base catalysis and the subsequent acidolysis with haloid acids. The mild reaction conditions, simple operation, good yield, and readily available and inexpensive starting materials make this protocol a valuable method for the preparation of various 3-halooxindoles on a large-scale industrial application.

## Supporting Information

File 1 Experimental details as well as compound characterization and spectral data of the products.
