# Supplementary material for: A novel methodology for the efficient synthesis of 3-monohalooxindoles by acidolysis of 3-phosphate-substituted oxindoles with haloid acids
Source: Beilstein J Org Chem. 2021 Sep 7;17:2321–8. doi: 10.3762/bjoc.17.150 (PMC8450974; doi:10.3762/bjoc.17.150)
Supplement: File 1 — Experimental details as well as compound characterization and spectral data of the products. [file Beilstein_J_Org_Chem-17-2321-s001.pdf]

BEILSTEIN JOURNAL OF ORGANIC CHEMISTRY

Supporting Information

for

A novel methodology for the efficient synthesis of 3-monohalooxindoles by acidolysis of 3-phosphate-substituted oxindoles with haloid acids

Li Liu, Yue Li, Tiao Huang, Dulin Kong and Mingshu Wu

Beilstein J. Org. Chem. **2021,** *17,* 2321–2328. doi:10.3762/bjoc.17.150

Experimental details as well as compound characterization and spectral data of the products

License and Terms: This is a supporting information file under the terms of the Creative Commons Attribution License (https://creativecommons.org/licenses/by/4.0). Please note that the reuse, redistribution and reproduction in particular requires that the author(s) and source are credited and that individual graphics may be subject to special legal provisions.
The license is subject to the *Beilstein Journal of Organic Chemistry* terms and conditions: (https://www.beilstein-journals.org/bjoc/terms)

Table of contents

I Experimental section

II Spectra of compounds **2**–**4**

References

I Experimental section

General methods

The reactions were monitored by thin-layer chromatography (TLC) using silica gel GF254. All compounds were fully characterized by spectroscopic data. The NMR spectra were recorded on a Bruker Avance III (^{1}H: 400 MHz, ^{13}C: 100 MHz, ^{19}F NMR: 377 MHz, ^{31}P: 162 MHz), chemical shifts (δ) are expressed in ppm, and *J* values are given in Hz. $CDCl_3$ and DMSO-d_6 were used as solvents. High-resolution mass spectra were recorded on LCMS-IT-TOF. All chemicals and solvents were used as received without further purification unless otherwise stated. Column chromatography was performed on silica gel (200–300 mesh).

General procedure for preparation of oxindole derivatives 2

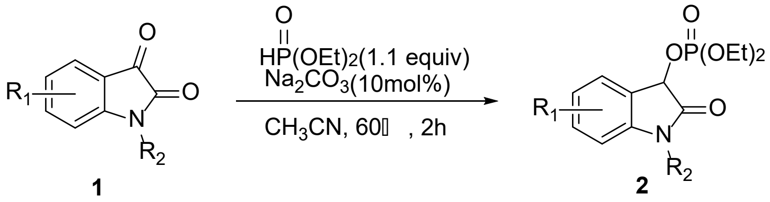

All 3-phosphate-substituted oxindoles were prepared according to a procedure described in the literature [1-3].

A 100-mL round-bottom flask was charged with an isatin **1** (10 mmol), Na_2CO_3 (1 mmol), diethyl phosphite (11 mmol), and CH_3CN (20 mL). The reaction mixture was stirred at 60 °C in a preheated oil bath until completion of the reaction (4 h). After

completion of the reaction, the residue was purified by column chromatography to provide **2**.

General procedure for preparation of compounds 3 and 4

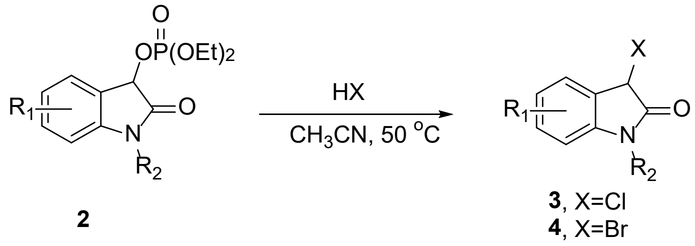

To a 10-mL screw-cap glass vial equipped with a magnetic stirrer was added CH_3CN (3mL), a diethyl (2-oxoindolin-3-yl)phosphate **2** (0.5 mmol), concentrated hydrochloric acid (0.64 mL, 36%, 7.5 mmol) or concentrated hydrobromic acid (1.10 mL, 40%, 7.5 mmol). Then, the reaction mixture was stirred at 50 °C for 6 h until completion of the reaction. The solvent was evaporated under vacuum and the residue mixture was directly purified by flash column chromatography on silica gel to obtain a product **3** or **4**.

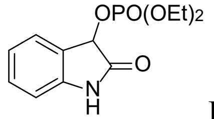

Diethyl (2-oxoindolin-3-yl) phosphate (2a): Brown oil (95%). **^{1}H NMR** (400 MHz, Chloroform-*d*) δ 9.44 (s, 1H), 7.41 (d, *J* = 7.5 Hz, 1H), 7.23 – 7.12 (m, 1H), 6.95 (td, *J* = 7.6, 1.0 Hz, 1H), 6.82 (d, *J* = 7.8 Hz, 1H), 5.49 (d, J_{P-H} = 12.9 Hz, 1H), 4.23 – 4.07 (m, 4H), 1.27 (m, *J* = 12.0, 7.1, 1.2 Hz, 6H). **^{13}C NMR** (100 MHz, Chloroform-*d*) δ 173.13 (d, *J* = 6.5 Hz), 141.02, 129.61, 125.00, 123.52 (d, *J* = 2.9 Hz), 121.82, 109.76, 71.91 (d, *J* = 5.9 Hz), 63.72 (d, *J* = 6.2 Hz), 63.48 (d, *J* = 6.0 Hz), 15.00 (d, *J* = 7.1 Hz, 2C). **^{31}P NMR** (162 MHz, Chloroform-*d*) δ -1.25(s).

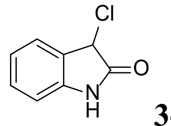

3-Chloroindolin-2-one (3a) [4]**:** Brown solid; mp: 165- 166 °C (92%). **^{1}H NMR** (400 MHz, DMSO-*d*6) δ 10.77 (s, 1H), 7.35 (d, J = 7.4 Hz, 1H), 7.28 (t, J = 7.7 Hz, 1H), 7.02 (s, 1H), 6.88 (d, J = 7.8 Hz, 1H), 5.55 (s, 1H). **^{13}C NMR** (100 MHz, DMSO-*d*6) δ 173.21, 142.43, 130.32, 126.51, 125.64, 122.32, 110.18, 52.25. **HRMS**

(ESI): m/z calcd for C_8H_6ClNO [M-H]$^-$: 166.0065; found: 166.0056.

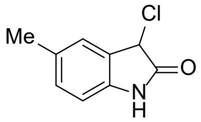

3-Chloro-5-methylindolin-2-one (3b) [5]: solid; mp: 96- 97 ºC (81%). **^{1}H NMR** (400 MHz, DMSO-*d*$_6$) δ 10.66 (s, 1H), 7.16 (s, 1H), 7.08 (d, *J* = 7.9 Hz, 1H), 6.76 (d, *J* = 7.9 Hz, 1H), 5.50 (s, 1H), 2.25 (d, *J* = 2.0 Hz, 3H). **^{13}C NMR** (100 MHz, DMSO-*d*$_6$) δ 173.2, 139.9, 131.4, 130.5, 126.5, 126.2, 109.9, 52.4, 20.5. **HRMS (ESI)**: m/z calcd for C_9H_8ClNO [M-H]$^-$: 180.0222; found: 180.0215.

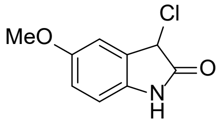

3-Chloro-5-methoxyindolin-2-one (3c): solid; mp: 106-107 ºC (81%). **^{1}H NMR** (400 MHz, DMSO-*d*$_6$) δ 10.58 (s, 1H), 6.98 (d, *J* = 2.5 Hz, 1H), 6.86 (dd, *J* = 8.5, 2.6 Hz, 1H), 6.79 (d, *J* = 8.5 Hz, 1H), 5.52 (s, 1H), 3.72 (s, 3H). **^{13}C NMR** (100 MHz, DMSO-*d*$_6$) δ 173.0, 155.2, 135.6, 127.6, 115.4, 112.1, 110.7, 55.6, 52.6. **HRMS (ESI)**: m/z calcd for $C_9H_8ClNO_2$ [M-H]$^-$: 196.0171; found: 196.0172.

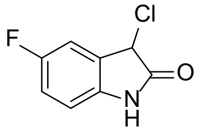

3-Chloro-5-fluoroindolin-2-one (3d): solid; mp: 183- 184 ºC (51%). **^{1}H NMR** (400 MHz, DMSO-*d6*) δ 10.79 (s, 1H), 7.28 (d, J = 8.1 Hz, 1H), 7.14 (t, J = 9.1 Hz, 1H), 6.87 (dd, J = 8.6, 4.4 Hz, 1H), 5.58 (s, 1H). **^{13}C NMR** (100 MHz, DMSO-*d6*) δ 173.1, 158.1 (d, $J_{F\text{-}C}$= 237.9 Hz), 138.6 (d, J = 2.0 Hz), 128.1 (d, J = 8.9 Hz), 116.7 (d, J = 23.3 Hz), 113.3 (d, J = 25.2 Hz), 111.1 (d, J = 8.1 Hz), 52.1 (d, J = 1.8 Hz). **^{19}F NMR** (377 MHz, DMSO-*d*$_6$) δ -120.90. **HRMS (ESI)**: m/z calcd for C_8H_5ClFNO [M-H]$^-$: 183.9971; found: 183.9964.

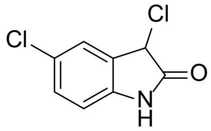

3,5-Dichloroindolin-2-one (3e) [5]**:** solid; mp: 200- 201 ºC (75%). **^{1}H NMR** (400 MHz, DMSO-*d*$_6$) δ 10.90 (s, 1H), 7.42 (d, *J* = 2.2 Hz, 1H), 7.34 (dd, *J* = 8.3, 2.2 Hz, 1H), 6.89 (s, 1H), 5.58 (s, 1H). **^{13}C NMR** (100 MHz, DMSO-*d*$_6$) δ 172.9, 141.3, 130.1, 128.5, 126.2, 125.7, 111.7, 51.7. **HRMS (ESI)**: m/z calcd for $C_8H_5Cl_2NO$ [M-H]$^-$: 199.9675; found: 199.9667.

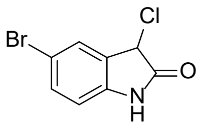

5-Bromo-3-chloroindolin-2-one (3f) [6]**:** solid; mp: 189-190 ºC (58%). **^{1}H NMR** (400 MHz, DMSO-*d*$_6$) δ 10.91 (s, 1H), 7.54 (d, *J* = 2.1 Hz, 1H), 7.47 (dd, *J* = 8.3, 2.1 Hz, 1H), 6.84 (d, *J* = 8.3 Hz, 1H), 5.58 (s, 1H). **^{13}C NMR** (100 MHz, DMSO-*d*$_6$) δ 172.7, 141.7, 133.0, 128.9, 128.4, 113.7, 112.1, 51.6. **HRMS (ESI)**: m/z

calcd for $C_8H_5BrClNO$ $[M-H]^-$: 243.9170; found: 243.9158.

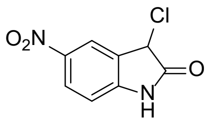

3-Chloro-5-nitroindolin-2-one (3g): solid; mp: 178-179ºC (69%). **^{1}H NMR** (400 MHz, DMSO-*d*$_6$) δ 11.46 (s, 1H), 8.32 – 7.86 (m, 2H), 7.21 – 6.83 (m, 1H), 5.67 (d, *J* = 3.3 Hz, 1H). **^{13}C NMR** (100 MHz, DMSO-*d*$_6$) δ 173.49, 148.75, 142.50, 127.54 (d, *J* = 3.5 Hz), 127.28 (d, *J* = 3.5 Hz), 121.23, 110.48 (d, *J* = 2.4 Hz), 51.08. **HRMS (ESI)**: m/z calcd for $C_8H_5ClN_2O_3$ $[M-H]^-$: 210.9916; found: 210.9909.

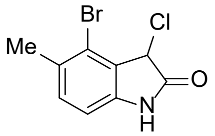

4-Bromo-3-chloro-5-methylindolin-2-one (3h): solid; mp: 181-182 ºC (82%). **^{1}H NMR** (400 MHz, DMSO-*d*$_6$) δ 10.88 (s, 1H), 7.25 (d, *J* = 7.9 Hz, 1H), 6.78 (d, *J* = 7.9 Hz, 1H), 5.40 (s, 1H), 2.27 (s, 3H). **^{13}C NMR** (100 MHz, DMSO-*d*$_6$) δ 172.0, 141.9, 132.3, 130.9, 126.0, 122.4, 109.3, 53.5, 21.4. **HRMS (ESI)**: m/z calcd for $C_9H_7BrClNO$ $[M-H]^-$: 257.9327; found: 257.9333.

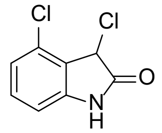

3,4-Dichloroindolin-2-one (3i): solid; mp: 168-169ºC (96%). **^{1}H NMR** (400 MHz, DMSO-*d*$_6$) δ 11.00 (s, 1H), 7.33 (s, 0H), 7.06 (d, *J* = 8.2 Hz, 1H), 6.85 (d, *J* = 7.8 Hz, 1H), 5.57 (s, 1H). **^{13}C NMR** (100 MHz, DMSO-*d*$_6$) δ 172.2, 144.3, 132.2, 131.0, 123.6, 122.4, 109.1, 51.4. **HRMS (ESI)**: m/z calcd for $C_8H_5Cl_2NO$ $[M-H]^-$: 199.9675; found: 199.9668.

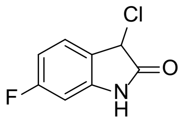

3-Chloro-6-fluoroindolin-2-one (3j) [5]**:** solid; mp: 165- 166 ºC (55%). **^{1}H NMR** (400 MHz, DMSO-*d*$_6$) δ 10.92 (s, 1H), 7.38 (t, *J* = 4.3 Hz, 1H), 6.82 (dd, *J* = 8.2, 2.3 Hz, 1H), 6.70 (d, *J* = 9.1 Hz, 1H), 5.52 (s, 1H). **^{13}C NMR** (100 MHz, DMSO-*d*$_6$) δ 173.5, 163.3 (d, $J_{F\text{-}C}$ = 245.1 Hz), 144.2 (d, *J* = 12.6 Hz), 127.4 (d, *J* = 10.3 Hz), 122.5 (d, *J* = 2.9 Hz), 108.6 (d, *J* = 22.7 Hz), 98.5 (d, *J* = 27.4 Hz), 51.6. **^{19}F NMR** (377 MHz, DMSO-*d*$_6$) δ -109.39. **HRMS (ESI)**: m/z calcd for C_8H_5ClFNO $[M - H]^-$: 183.9971; found: 199.9961.

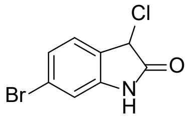

6-Bromo-3-chloroindolin-2-one (3k): solid; mp: 185- 186 ºC (51%). **^{1}H NMR** (400 MHz, DMSO-*d*$_6$) δ 10.92 (s, 1H), 7.32 (d, *J* = 8.0 Hz, 1H), 7.23 (d, *J* = 1.7 Hz, 1H), 7.03 (d, *J* = 1.8 Hz, 1H), 5.56 (s, 1H). **^{13}C NMR** (100 MHz, DMSO-*d*$_6$) δ 173.0, 144.0, 127.4, 125.8, 125.0, 122.9, 113.0, 51.6. **HRMS (ESI)**: m/z calcd for

$C_8H_5BrClNO$ [M-H]$^-$: 243.9170; found: 243.9161.

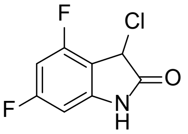

3-Chloro-4,6-*d*ifluoroindolin-2-one (3l): Yellow solid; mp: 179- 180 ºC (80%). **^{1}H NMR** (400 MHz, DMSO-*d6*) δ 11.19 (s, 1H), 6.87 (t, *J* = 10.0 Hz, 1H), 6.63 (d, *J* = 8.7 Hz, 1H), 5.69 (s, 1H). **^{13}C NMR** (100 MHz, DMSO-d_6) δ 172.7, 164.1 (dd, $J_{F\text{-}C}$ = 247.5, 13.3 Hz), 158.7 (dd, $J_{F\text{-}C}$ = 251.3, 15.2 Hz), 145.5 (dd, *J* = 15.1, 10.3 Hz), 108.7 (d, *J* = 16.3 Hz), 97.6 (m), 95.4 (d, *J* = 27.3 Hz), 48.9. **^{19}F NMR** (377 MHz, DMSO-d_6) δ -105.22 (d, J = 8.9 Hz), -112.69 (d, J = 8.9 Hz). **HRMS (ESI)**: m/z calcd for $C_8H_4ClF_2NO$ [M-H]$^-$: 201.9877; found: 201.9866.

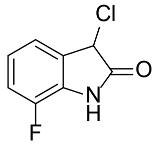

3-Chloro-7-fluoroindolin-2-one (3m) [7]**:** solid; mp: 165- 166 ºC (72%). **^{1}H NMR** (400 MHz, DMSO-d_6) δ 11.31 (s, 1H), 7.25 – 7.17 (m, 2H), 7.04 (td, *J* = 8.2, 4.7 Hz, 1H), 5.64 (s, 1H). **^{13}C NMR** (100 MHz, DMSO-d_6) δ 173.0, 146.3 (d, $J_{F\text{-}C}$ = 243.3 Hz), 129.6 (d, *J* = 13.0 Hz), 129.3 (d, *J* = 3.8 Hz), 123.2 (d, *J* = 6.0 Hz), 121.7 (d, *J* = 3.3 Hz), 117.3 (d, *J* = 17.1 Hz), 51.9 (d, *J* = 3.3 Hz). **^{19}F NMR** (377 MHz, DMSO-d_6) δ -132.21. **HRMS (ESI)**: m/z calcd for C_8H_5ClFNO [M-H]$^-$: 183.9971; found: 183.9971.

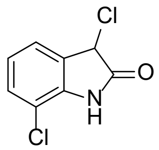

3,7-Dichloroindolin-2-one (3n): solid; mp: 172- 173 ºC (72%). **^{1}H NMR** (400 MHz, DMSO-d_6) δ 11.22 (s, 1H), 7.54 – 7.25 (m, 2H), 7.05 (t, *J* = 7.8 Hz, 1H), 5.67 (s, 1H). **^{13}C NMR** (100MHz, DMSO-d_6) δ 173.1, 140.1, 130.2, 128.3, 124.3, 123.6, 114.3, 52.3. **HRMS (ESI)**: m/z calcd for $C_8H_5Cl_2NO$ [M-H]$^-$: 199.9675; found: 199.9667.

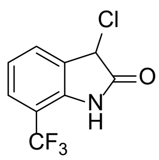

3-Chloro-7-(trifluoromethyl)indolin-2-one (3o) [7]**:** solid; mp: 121- 122 ºC (55%). **^{1}H NMR** (400 MHz, DMSO-d_6) δ 11.27 (s, 1H), 7.67 (d, *J* = 7.5 Hz, 1H), 7.59 (d, *J* = 8.1 Hz, 1H), 7.22 (t, *J* = 7.8 Hz, 1H), 5.67 (s, 1H). **^{13}C NMR** (100 MHz, DMSO-d_6) δ 173.6, 139.7 (d, *J* = 2.4 Hz), 129.7, 128.5, 126.6 (d, *J* = 4.5 Hz), 123.3 (d, $J_{F\text{-}C}$= 271.8 Hz), 122.5, 111.3 (d, *J* = 33.1 Hz), 50.7. **^{19}F NMR** (377 MHz, DMSO-d_6) δ -60.31. **HRMS (ESI)**: m/z calcd for $C_9H_5ClF_3NO$ [M-H]$^-$: 233.9939; found: 233.9930.

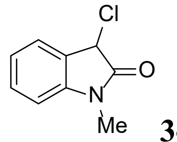

3-Chloro-1-methylindolin-2-one (3p) [8]**:** Yellow solid; mp: 152- 153 ºC (88%). ^{1}H NMR (400 MHz, DMSO-d_6) δ 7.46 – 7.35 (m, 2H), 7.11 (t, *J* = 7.6 Hz, 1H), 7.05 (dd, *J* = 7.9, 2.2 Hz, 1H), 5.65 (s, 1H), 3.14 (s, 3H). **^{13}C NMR** (100 MHz, DMSO-d_6) δ 173.2, 139.9, 131.4, 130.5, 126.5, 126.1, 109.9, 52.4, 20.5. **HRMS (ESI)**: m/z calcd for C_9H_8ClNO [M-H]$^-$: 180.0222; found: 180.0210.

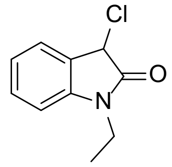

3-Chloro-1-ethylindolin-2-one (3q) [9]**:** Brown solid; mp: 69-70 ºC (73%). **^{1}H NMR** (400 MHz, DMSO-d_6) δ 7.48 – 7.35 (m, 2H), 7.12 (d, *J* = 7.8 Hz, 2H), 5.66 (s, 1H), 3.71 (d, *J* = 7.1 Hz, 2H), 1.15 (s, 3H). **^{13}C NMR** (100 MHz, DMSO-d_6) δ 171.1, 142.7, 130.4, 125.8, 125.5, 122.8, 109.3, 51.7, 34.5, 12.3. **HRMS (ESI)**: m/z calcd for $C_{10}H_{10}ClNO$ [M-H]$^-$: 194.0378; found: 194.0380.

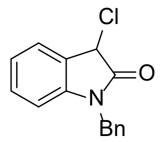

1-Benzyl-3-chloroindolin-2-one (3r) [8]**:** solid; mp: 139-140 ºC (75%). **^{1}H NMR** (400 MHz, DMSO-d_6) δ 7.45 (d, *J* = 7.4 Hz, 1H), 7.36 – 7.26 (m, 6H), 7.10 (td, *J* = 7.6, 1.0 Hz, 1H), 6.98 (d, *J* = 7.9 Hz, 1H), 5.82 (s, 1H), 4.93 (d, *J* = 1.6 Hz, 2H).**^{13}C NMR** (100 MHz, DMSO-d_6) δ 171.8, 142.7, 135.8, 130.3, 128.7 (2C), 127.6, 127.2 (2C), 125.8, 125.5, 123.1, 109.8, 51.6, 42.9. **HRMS (ESI)**: m/z calcd for $C_{15}H_{12}ClNO$ [M+H] $^+$: 258.0680; found: 258.0669.

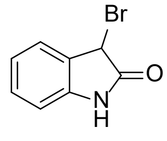

3-Bromoindolin-2-one (4a) [10]**:** Brown solid; mp: 68-69 ºC (73%). **^{1}H NMR** (400 MHz, DMSO-d_6) δ 10.76 (s, 1H), 7.33 (d, *J* = 7.5 Hz, 1H), 7.26 (m, *J* = 7.7, 1.0 Hz, 1H), 7.01 (m, *J* = 7.5, 1.0 Hz, 1H), 6.85 (dd, *J* = 7.8, 0.9 Hz, 1H), 5.70 (s, 1H). **^{13}C NMR** (100 MHz, DMSO-d_6) δ 173.5, 142.4, 130.2, 127.0, 126.0, 122.3, 110.1, 40.5. **HRMS (ESI)**: m/z calcd for C_8H_6BrNO [M-H]$^-$: 209.9560; found: 209.9554.

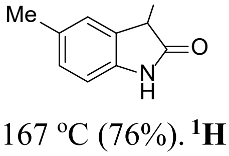

3-Bromo-5-methylindolin-2-one (4b) [11]**:** Brown solid; mp: 166-167 ºC (76%). **^{1}H NMR** (400 MHz, DMSO-d_6) δ 10.65 (s, 1H), 7.12 (s, 1H), 7.04 (dd, *J* = 7.9, 1.8 Hz, 1H), 6.74 (d, *J* = 7.9 Hz, 1H), 5.63 (s, 1H), 2.23 (s, 3H). **^{13}C NMR** (100 MHz, DMSO-d_6) δ 173.5, 139.9, 131.3, 130.5, 127.0, 126.5, 109.9, 40.7, 20.5. **HRMS (ESI)**: m/z calcd for C_9H_8BrNO [M-H]$^-$: 223.9717; found: 223.9712.

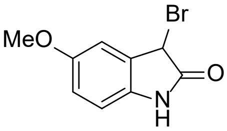

3-Bromo-5-methoxyindolin-2-one (4c): Yellow solid; mp: 166-167 °C (75%). **^{1}H NMR** (400 MHz, DMSO-d_6) δ 10.59 (s, 1H), 6.95 (d, J = 2.6 Hz, 1H), 6.83 (d, J = 2.6 Hz, 1H), 6.77 (d, J = 8.5 Hz, 1H), 5.65 (s, 1H), 3.71 (s, 3H). **^{13}C NMR** (100 MHz, DMSO-d_6) δ 173.5, 155.2, 135.6, 128.1, 115.4, 112.3, 110.7, 55.6, 40.9. **HRMS (ESI)**: m/z calcd for $C_9H_8BrNO_2$ $[M-H]^-$: 239.9666; found: 239.9654.

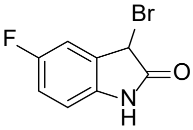

3-Bromo-5-fluoroindolin-2-one (4d): Brown solid; mp: 167- 168 °C (55%). **^{1}H NMR** (400 MHz, DMSO-d_6) δ 10.79 (s, 1H), 7.30 – 7.20 (m, 1H), 7.11 (d, J = 2.8 Hz, 1H), 6.86 (dd, J = 8.5, 4.4 Hz, 1H), 5.70 (s, 1H). **^{13}C NMR** (100 MHz, DMSO-d_6) δ 173.55, 158.07 (d, J_{F-C} = 237.7 Hz), 138.60 (d, J = 2.2 Hz), 128.65 (d, J = 9.1 Hz), 116.64 (d, J = 23.4 Hz), 113.53 (d, J = 25.2 Hz), 111.10 (d, J = 8.1 Hz), 40.00 (d, J = 1.9 Hz). **^{19}F NMR** (377 MHz, DMSO-d_6) δ -121.02. **HRMS (ESI)**: m/z calcd for C_8H_5BrFNO $[M-H]^-$: 227.9466; found: 227.9462.

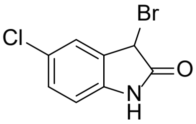

3-Bromo-5-chloroindolin-2-one (4e) [11]**:** Brown solid; mp: 122-123 °C (58%). **^{1}H NMR** (400 MHz, DMSO-d_6) δ 10.89 (s, 1H), 7.39 (d, J = 2.2 Hz, 1H), 7.34 – 7.26 (m, 1H), 6.87 (d, J = 8.3 Hz, 1H), 5.70 (s, 1H). **^{13}C NMR** (100 MHz, DMSO-d_6) δ 173.34, 141.31, 130.04, 129.11, 126.20, 125.95, 111.74, 39.65. **HRMS (ESI)**: m/z calcd for $C_8H_5BrClNO$ $[M-H]^-$: 243.9170; found: 243.9182.

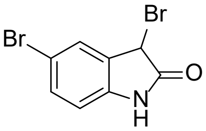

3,5-Dibromoindolin-2-one (4f): Brown solid; mp: 138- 139 °C (53%). **^{1}H NMR** (400 MHz, DMSO-d_6) δ 10.90 (s, 1H), 7.50 (s, 1H), 7.44 (dd, J = 8.3, 2.1 Hz, 1H), 6.82 (d, J = 8.3 Hz, 1H), 5.70 (s, 1H). **^{13}C NMR** (100 MHz, DMSO-d_6) δ 172.8, 141.7, 133.0, 128.9, 128.4, 113.8, 112.2, 51.6. **HRMS (ESI)**: m/z calcd for $C_8H_5Br_2NO$ $[M-H]^-$: 289.8645; found: 289.8646.

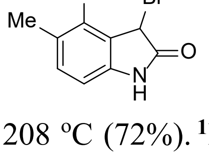

3,4-Dibromo-5-methylindolin-2-one (4g): Brown solid; mp: 207-208 °C (72%). **^{1}H NMR** (400 MHz, DMSO-d_6) δ 10.87 (s, 1H), 7.24 (d, J = 7.9 Hz, 1H), 6.78 (d, J = 7.8 Hz, 1H), 5.49 (s, 1H), 2.27 (s, 3H). **^{13}C NMR** (100 MHz, DMSO-d_6) δ 172.6, 141.8, 132.2, 130.7, 126.4, 122.5, 109.2, 41.9, 21.5. **HRMS (ESI)**: m/z calcd for $C_9H_7Br_2NO$ $[M-H]^-$: 303.8801; found: 303.8788.

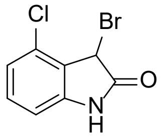

3-Bromo-4-chloroindolin-2-one (4h): Brown solid; mp: 168- 169 ºC (66%). **^{1}H NMR** (400 MHz, DMSO-*d_6*) δ 10.99 (s, 1H), 7.29 (d, *J* = 8.0 Hz, 1H), 7.04 (dd, *J* = 8.3, 0.8 Hz, 1H), 6.85 (d, *J* = 7.8 Hz, 1H), 5.65 (s, 1H). **^{13}C NMR** (100 MHz, DMSO-*d_6*) δ 172.77, 144.21, 132.01, 131.02, 124.14, 122.34, 109.09, 39.24. **HRMS (ESI)**: m/z calcd for $C_8H_5BrClNO$ $[M-H]^-$: 243.9170; found: 243.9169.

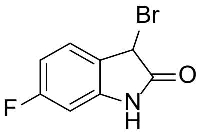

3-Bromo-6-fluoroindolin-2-one (4i): Brown solid; mp: 147- 148 ºC (71%). **^{1}H NMR** (400 MHz, DMSO-*d_6*) δ 10.91 (s, 1H), 7.35 (dd, *J* = 8.4, 5.5 Hz, 1H), 6.80 (d, *J* = 2.1 Hz, 1H), 6.68 (dd, *J* = 9.1, 2.5 Hz, 1H), 5.66 (s, 1H). **^{13}C NMR** (100 MHz, DMSO-*d_6*) δ 174.0, 163.1 (d, J_{F-C}= 245.1 Hz), 144.1 (d, *J* = 12.7 Hz), 127.7 (d, *J* = 10.3 Hz), 123.0 (d, *J* = 2.9 Hz), 108.6 (d, *J* = 22.8 Hz), 98.4 (d, *J* = 27.4 Hz), 39.7. **^{19}F NMR** (377 MHz, DMSO-*d_6*) δ -109.40. **HRMS (ESI)**: m/z calcd for C_8H_5BrFNO $[M-H]^-$: 227.9466; found: 227.9461.

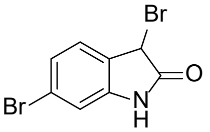

3,6-Dibromoindolin-2-one (4j): Brown solid; mp: 163- 164 ºC (63%). ^{1}H NMR (400 MHz, DMSO-*d_6*) δ 10.92 (s, 1H), 7.27 (d, *J* = 8.0 Hz, 1H), 7.18 (dd, *J* = 8.0, 1.8 Hz, 1H), 7.02 (d, *J* = 1.8 Hz, 1H), 5.67 (s, 1H). **^{13}C NMR** (100 MHz, DMSO-*d_6*) δ 173.45, 143.94, 127.72, 126.43, 124.97, 122.78, 113.06, 39.58. **HRMS (ESI)**: m/z calcd for $C_8H_5Br_2NO$ $[M+H]^+$: 290.8717; found: 290.8730.

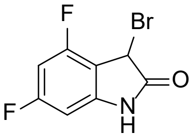

3-Bromo-4,6-*d*ifluoroindolin-2-one (4k): Yellow solid; mp: 156- 157 ºC (78%). **^{1}H NMR** (400 MHz, DMSO-*d6*) δ 11.18 (s, 1H), 6.85 (t, *J* = 10.0 Hz, 1H), 6.62 (d, *J* = 8.7 Hz, 1H), 5.77 (s, 1H). **^{13}C NMR (**100 MHz, DMSO-*d_6*) δ 173.3, 164.1 (dd, *J* = 247.7, 13.3 Hz), 158.6 (dd, *J* = 251.7, 15.1 Hz), 145.4 (dd, *J* = 15.0, 10.3 Hz), 109.3 (d, *J* = 19.4 Hz), 97.5 (m), 95.4 (d, *J* = 27.5 Hz), 36.0. **^{19}F NMR** (377 MHz, DMSO-*d_6*) δ -105.36 (d, *J* = 9.0 Hz), -111.67 (d, *J* = 9.0 Hz). **HRMS (ESI)**: m/z calcd for $C_8H_4BrF_2NO$ $[M+Na]^+$: 269.9337; found: 269.9349.

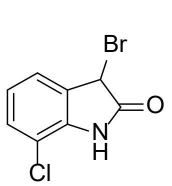

3-Bromo-7-chloroindolin-2-one (4l): Brown solid; mp: 166- 167 ºC (58%). **^{1}H NMR** (400 MHz, DMSO-*d_6*) δ 11.20 (s, 1H), 7.30 (t, *J* = 7.7 Hz, 2H), 7.02 (t, *J* = 7.8 Hz, 1H), 5.78 (s, 1H). **^{13}C NMR** (100 MHz, DMSO-*d_6*) δ 173.94, 140.48, 130.41, 129.28, 125.07, 123.98, 114.65, 40.69. **HRMS (ESI)**: m/z calcd for

$C_8H_5BrClNO$ [M-H]$^-$: 243.9170; found: 243.9168.

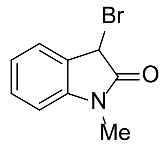

3-Bromo-1-methylindolin-2-one (4m) [8]**:** Brown solid; mp: 107- 108 °C (78%). **^{1}H NMR** (400 MHz, DMSO-*d*$_6$) δ 7.43 – 7.31 (m, 2H), 7.09 (td, *J* = 7.6, 1.0 Hz, 1H), 7.03 (d, *J* = 7.8 Hz, 1H), 5.79 (s, 1H), 3.14 (s, 3H). ^{13}C NMR (100 MHz, DMSO-*d*$_6$) δ 171.85, 143.68, 130.24, 126.17, 125.66, 122.90, 109.25, 39.60, 26.44. **HRMS (ESI)**: m/z calcd for C_9H_5BrNO [M+H]$^+$: 225.9862; found: 225.9858.

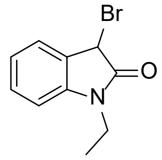

3-Bromo-1-ethylindolin-2-one (4n) [8]**:** Brown solid; mp: 112-113 °C (60%). **^{1}H NMR** (400 MHz, DMSO-*d*$_6$) δ 7.39 – 7.31 (m, 2H), 7.07 (t, *J* = 7.5 Hz, 2H), 5.77 (s, 1H), 3.69 (q, *J* = 7.2 Hz, 2H), 1.13 (t, *J* = 7.1 Hz, 3H). **^{13}C NMR** (100 MHz, DMSO-*d*$_6$) δ 171.51, 142.64, 130.25, 126.33, 125.91, 122.75, 109.26, 39.74, 34.59, 12.29. **HRMS (ESI)**: m/z calcd for $C_{10}H_{10}BrNO$ [M+H]$^+$: 240.0019; found: 240.0007.

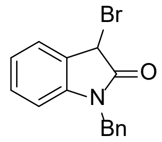

1-Benzyl-3-bromoindolin-2-one (4o) [8]**:** Brown solid; mp: 127-128 °C (62%). **^{1}H NMR** (400 MHz, DMSO-*d*$_6$) δ 7.42 (d, *J* = 7.4 Hz, 1H), 7.34 (d, *J* = 5.2 Hz, 4H), 7.31 – 7.23 (m, 1H), 7.08 (dd, *J* = 7.6, 1.0 Hz, 1H), 6.95 (d, *J* = 7.9 Hz, 1H), 5.93 (s, 1H), 4.92 (d, *J* = 9.1 Hz, 2H). **^{13}C NMR** (100 MHz, DMSO-*d*$_6$) δ 172.23, 142.65, 135.87, 130.14, 128.71, 127.56, 127.16, 126.29, 125.93, 123.06, 109.81, 42.92, 39.57. **HRMS (ESI)**: m/z calcd for $C_{15}H_{12}BrNO$ [M+H]$^+$: 302.0175; found: 302.0164.

II Spectra of compounds 2–4

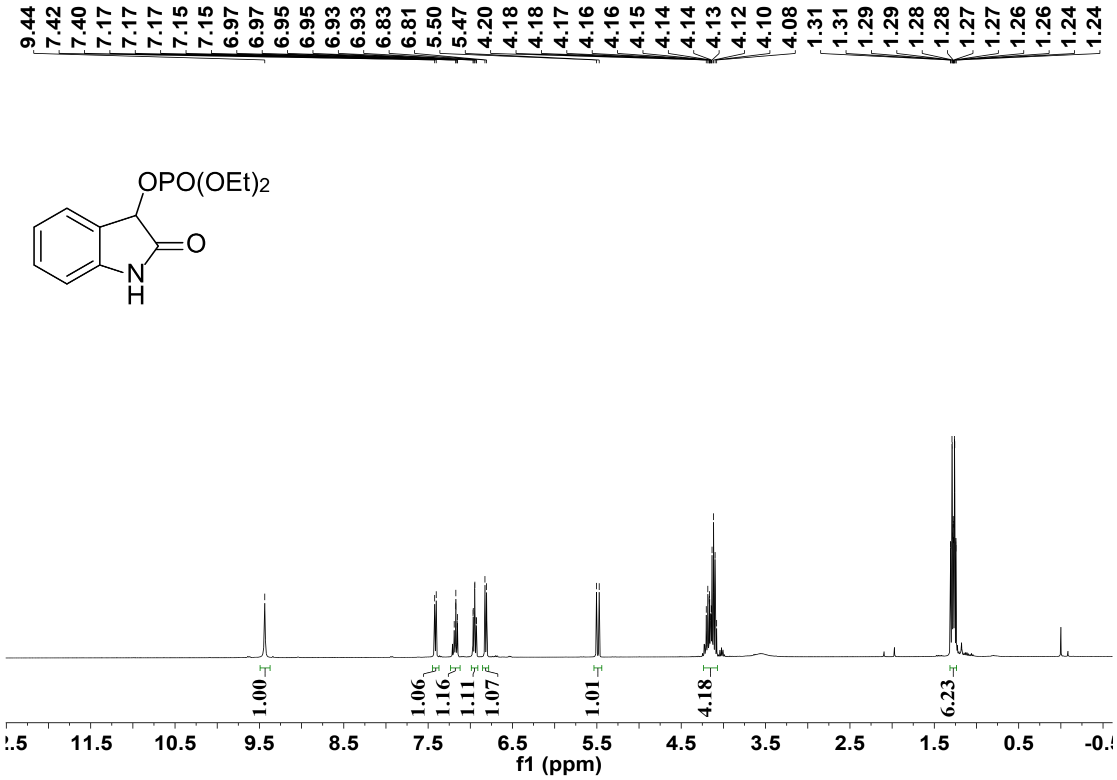

^{1}**H NMR** spectrum (400 MHz, DMSO-d_6) of **2a**

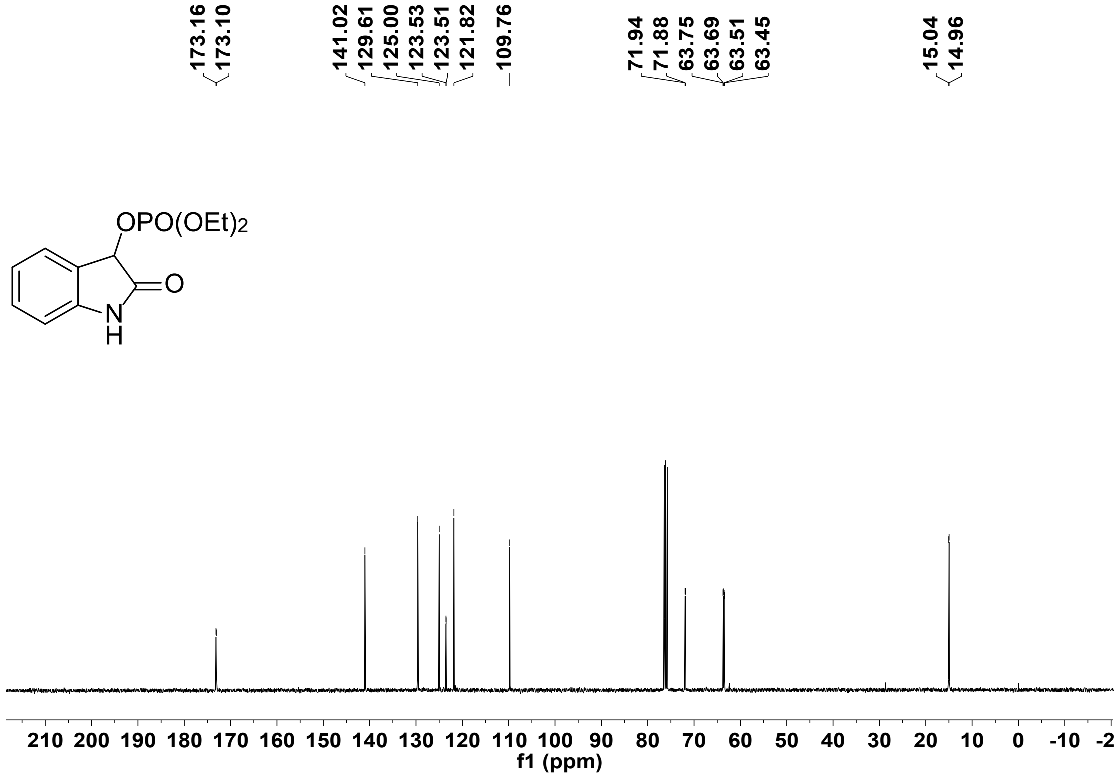

^{13}**C NMR** spectrum (100 MHz, DMSO-d_6) of **2a**

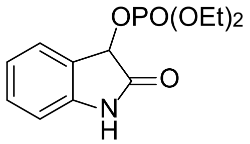

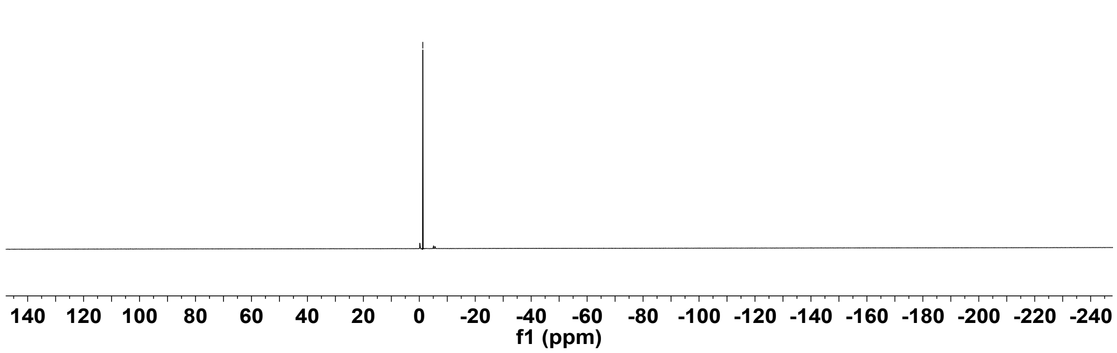

^{31}P NMR spectrum (377 MHz, DMSO-d_6) of **2a**

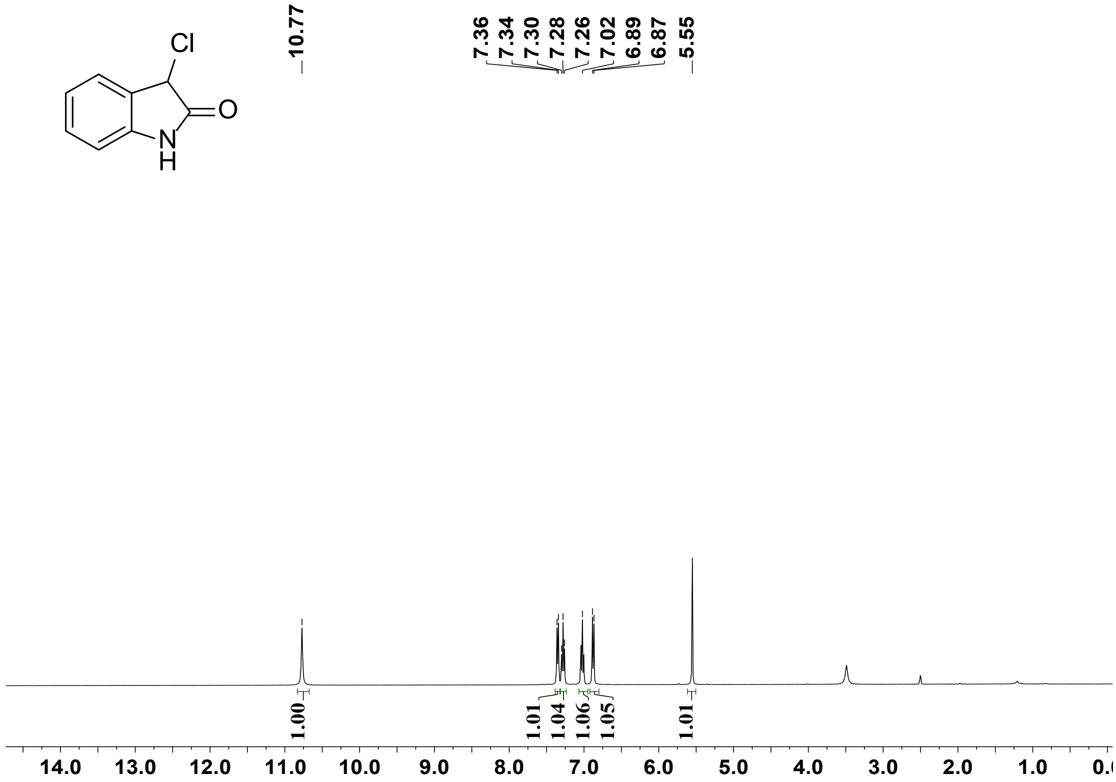

^{1}H NMR spectrum (400 MHz, DMSO-d_6) of **3a**

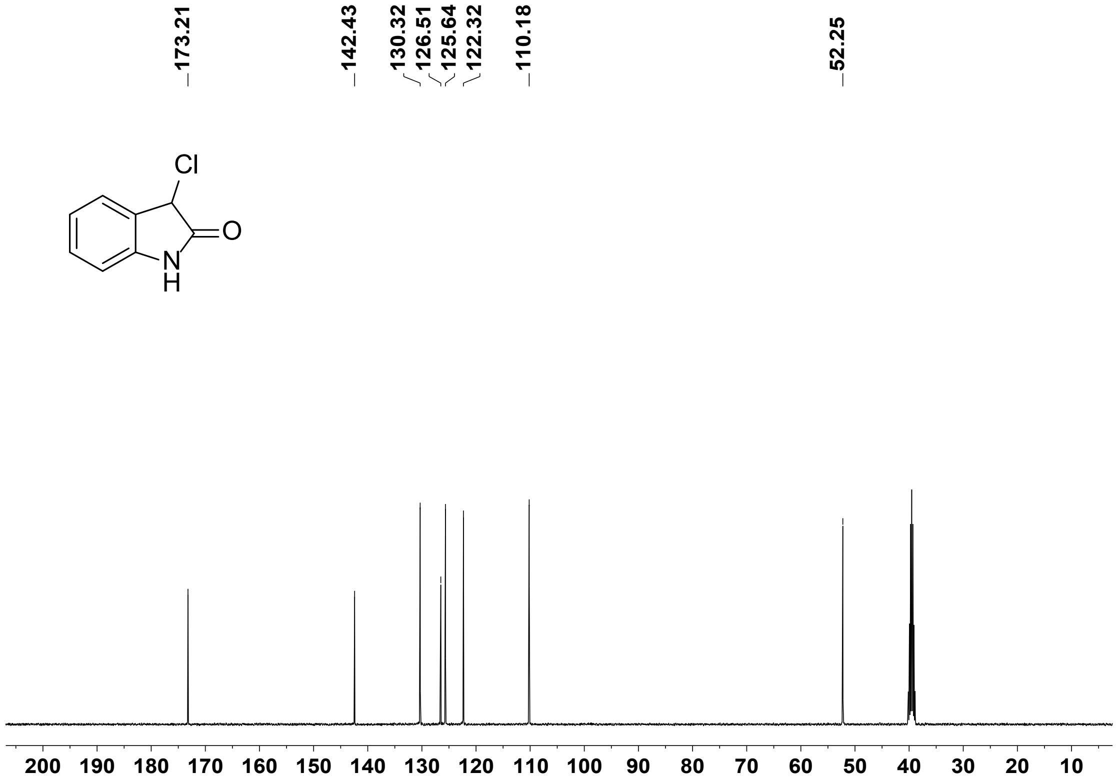

^{13}C NMR spectrum (100 MHz, DMSO-d_6) of **3a**

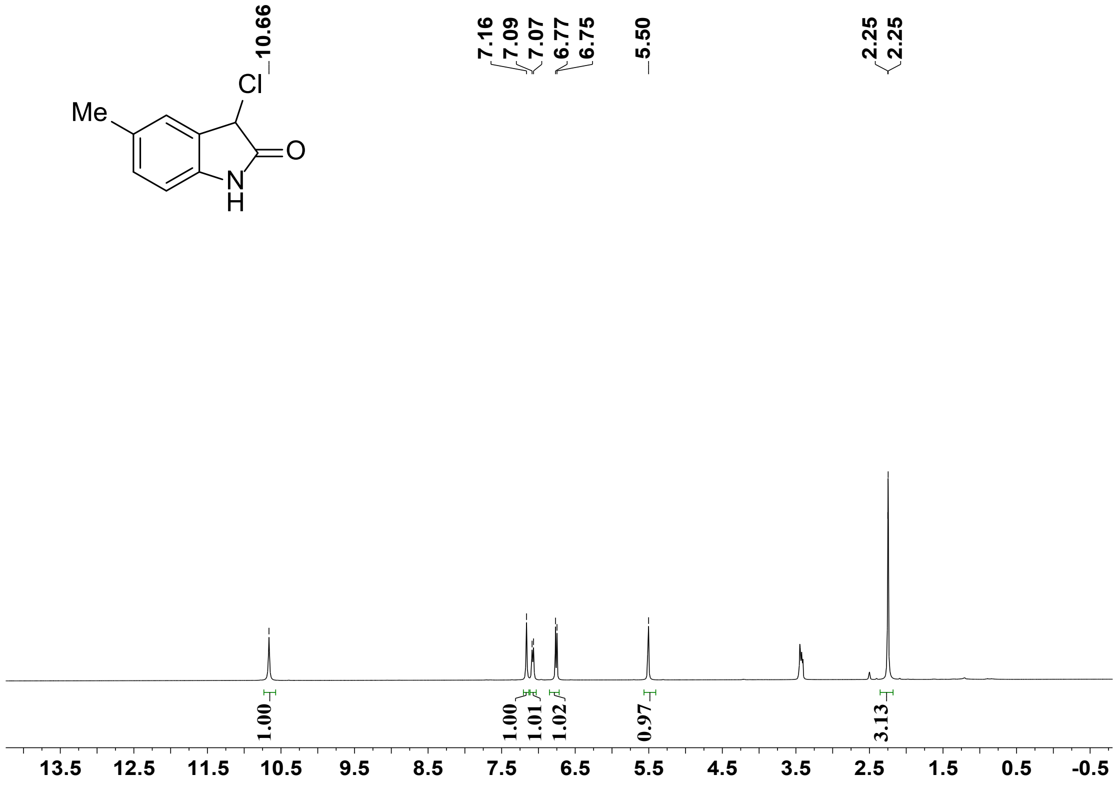

^{1}H NMR spectrum (400 MHz, DMSO-d_6) of **3b**

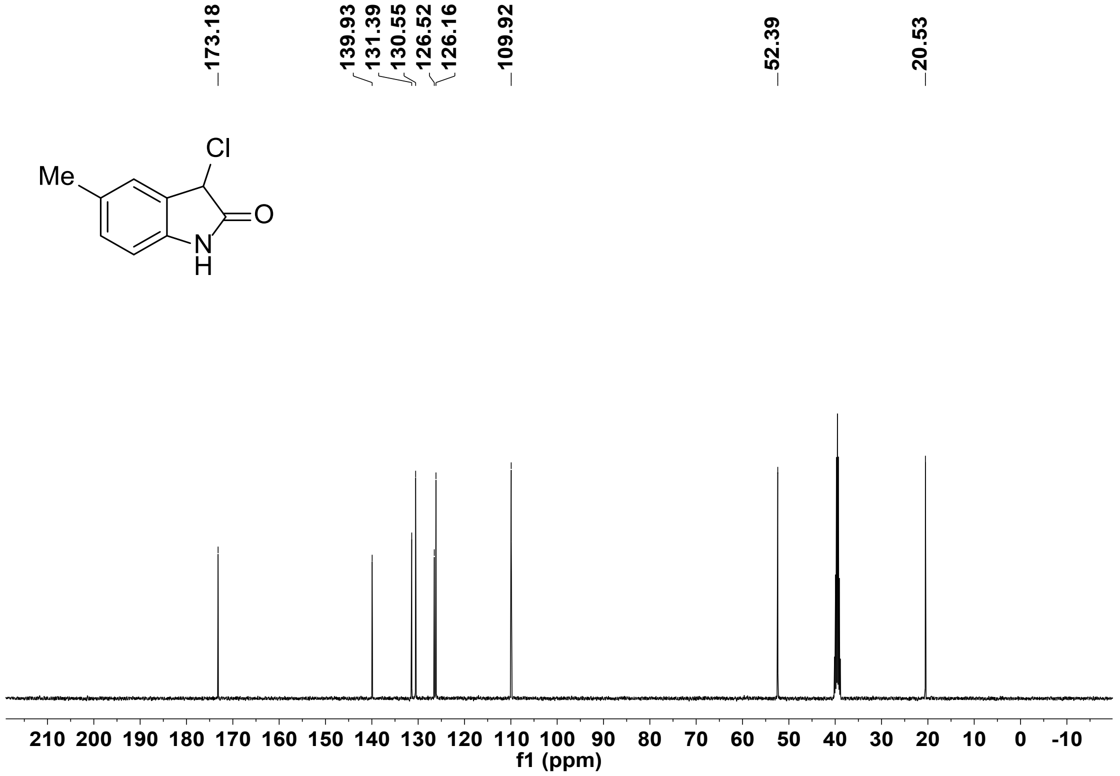

^{13}C NMR spectrum (100 MHz, DMSO-d_6) of **3b**

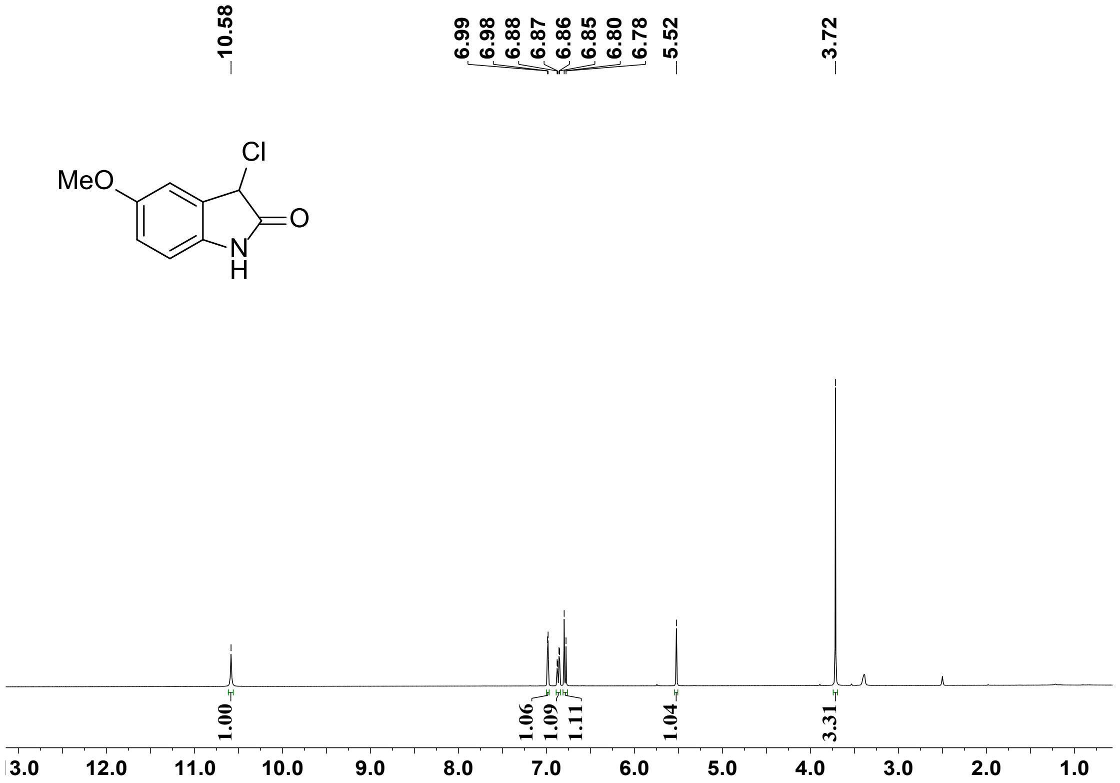

^{1}H NMR spectrum (400 MHz, DMSO-d_6) of **3c**

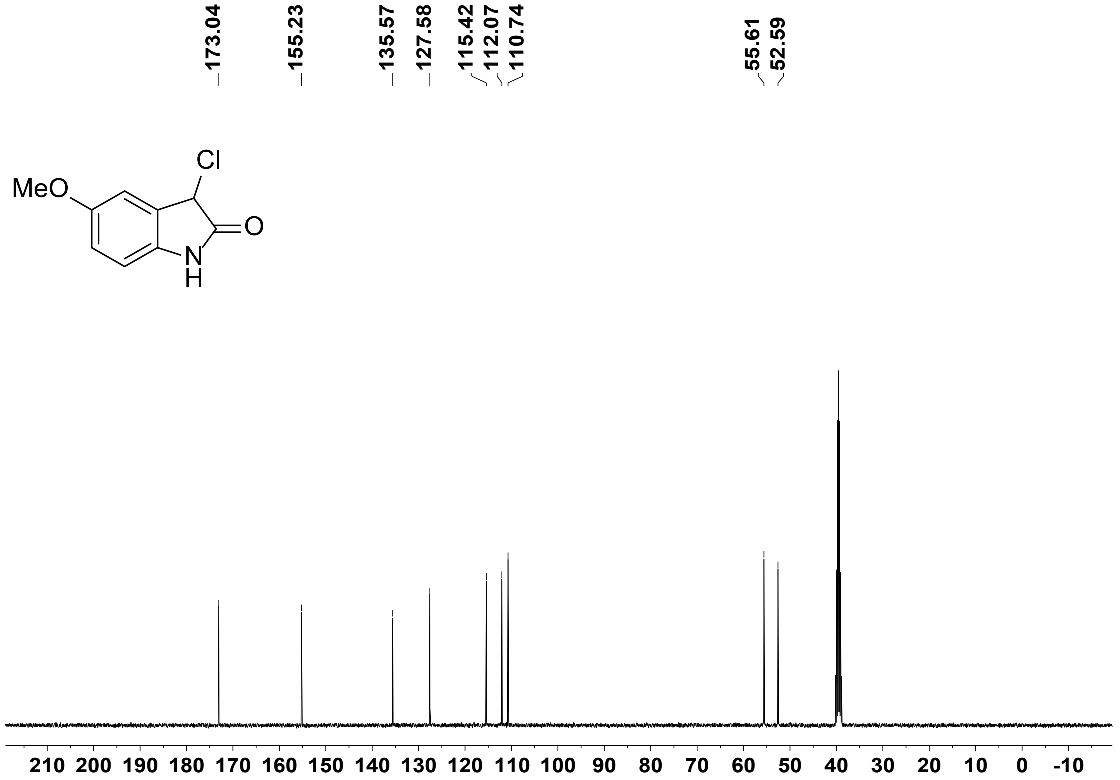

^{13}C NMR spectrum (100 MHz, DMSO-d_6) of **3c**

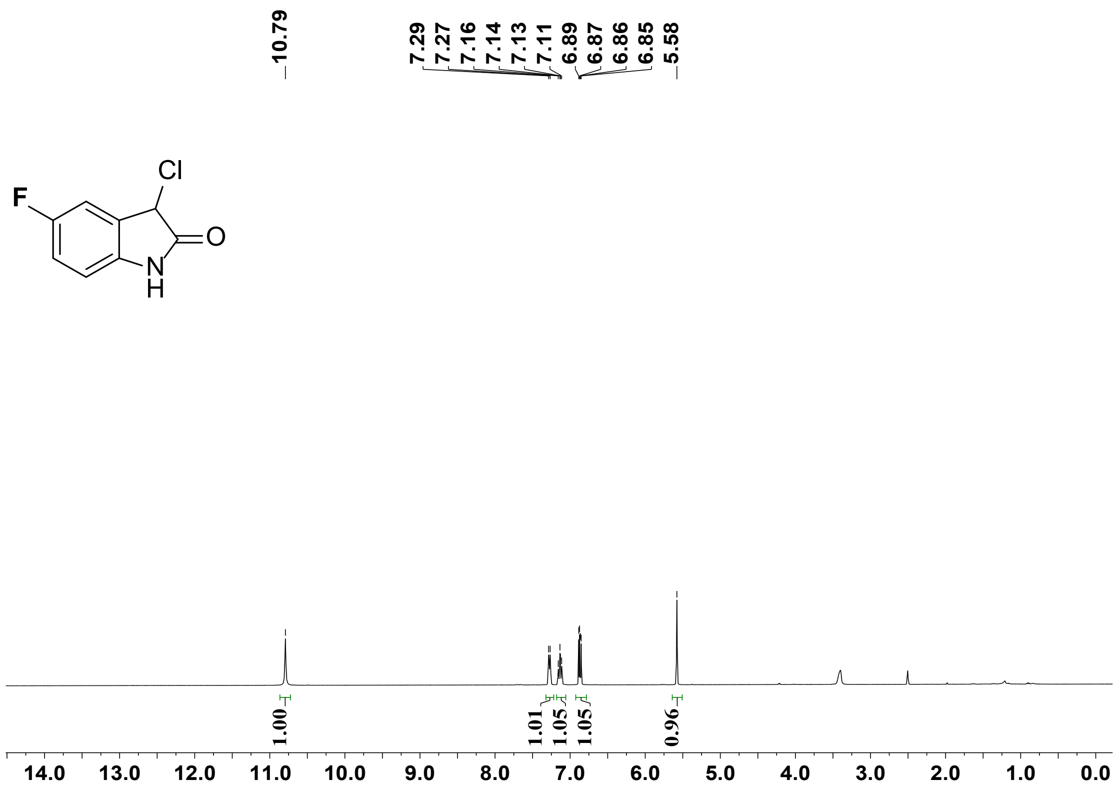

^{1}H NMR spectrum (400 MHz, DMSO-d_6) of **3d**

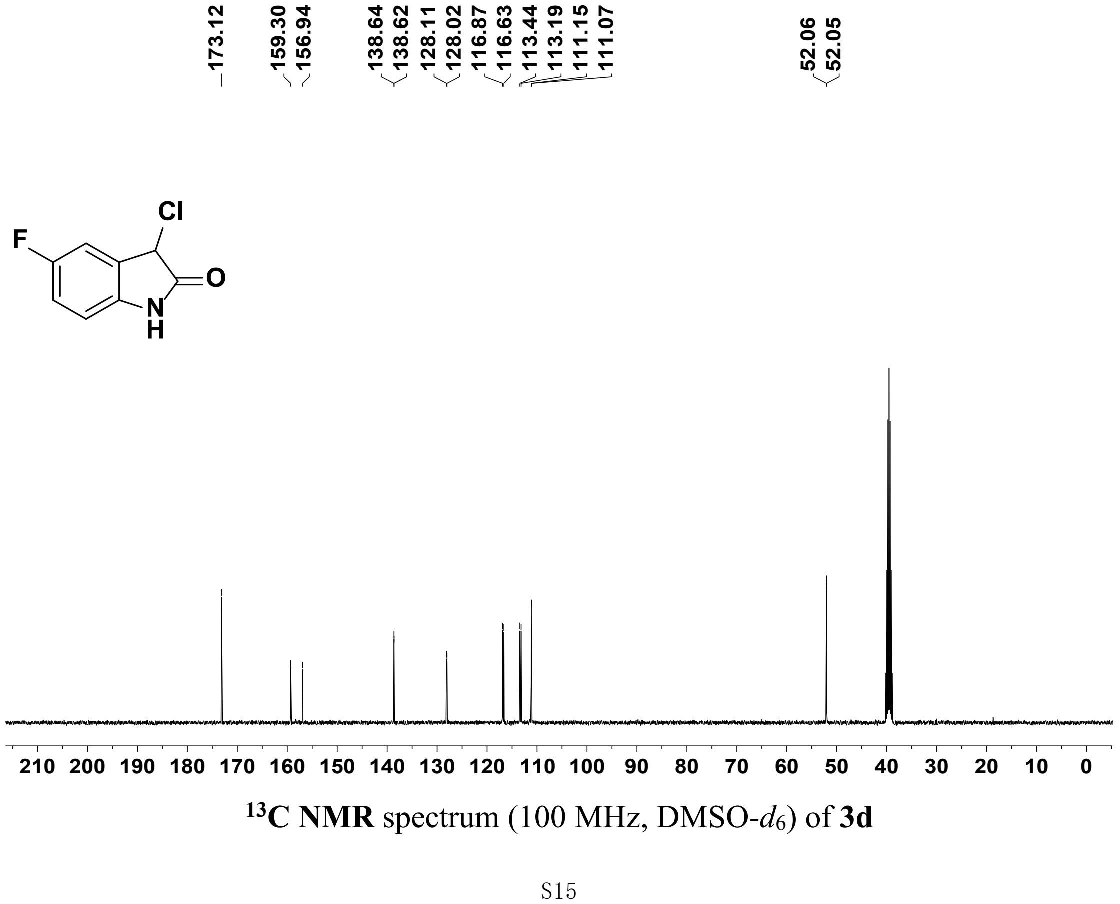

^{13}C NMR spectrum (100 MHz, DMSO-d_6) of **3d**

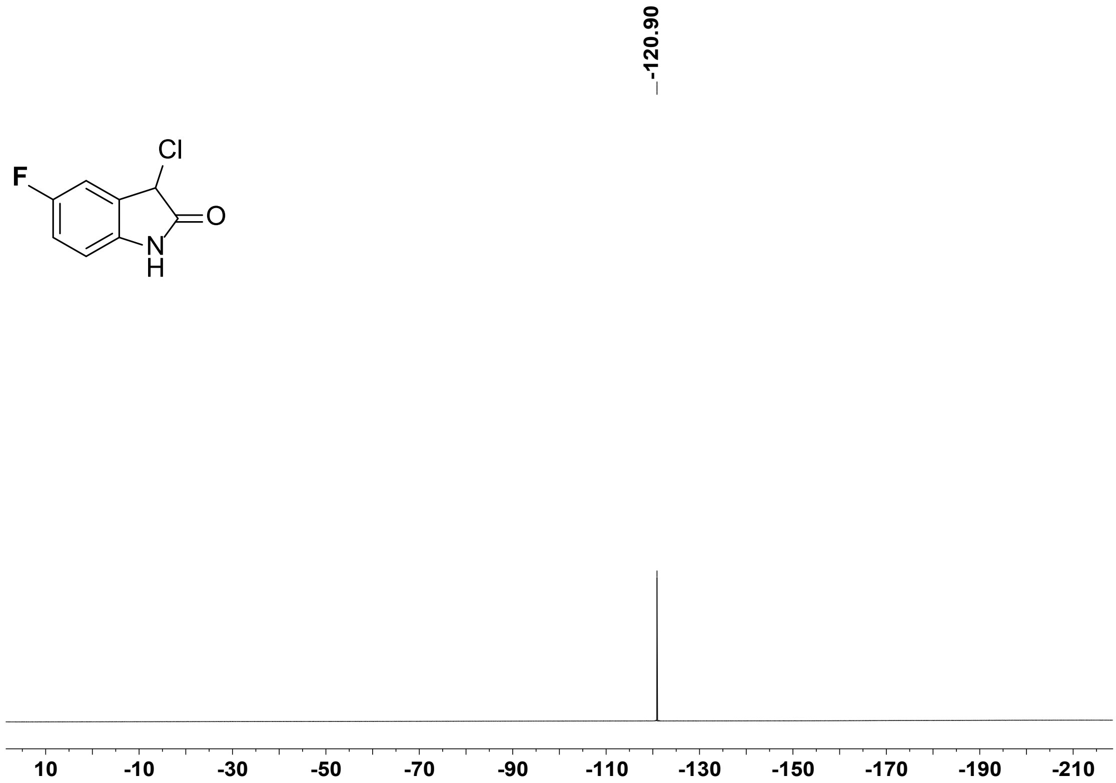

^{19}F NMR spectrum (377 MHz, DMSO-d_6) of **3d**

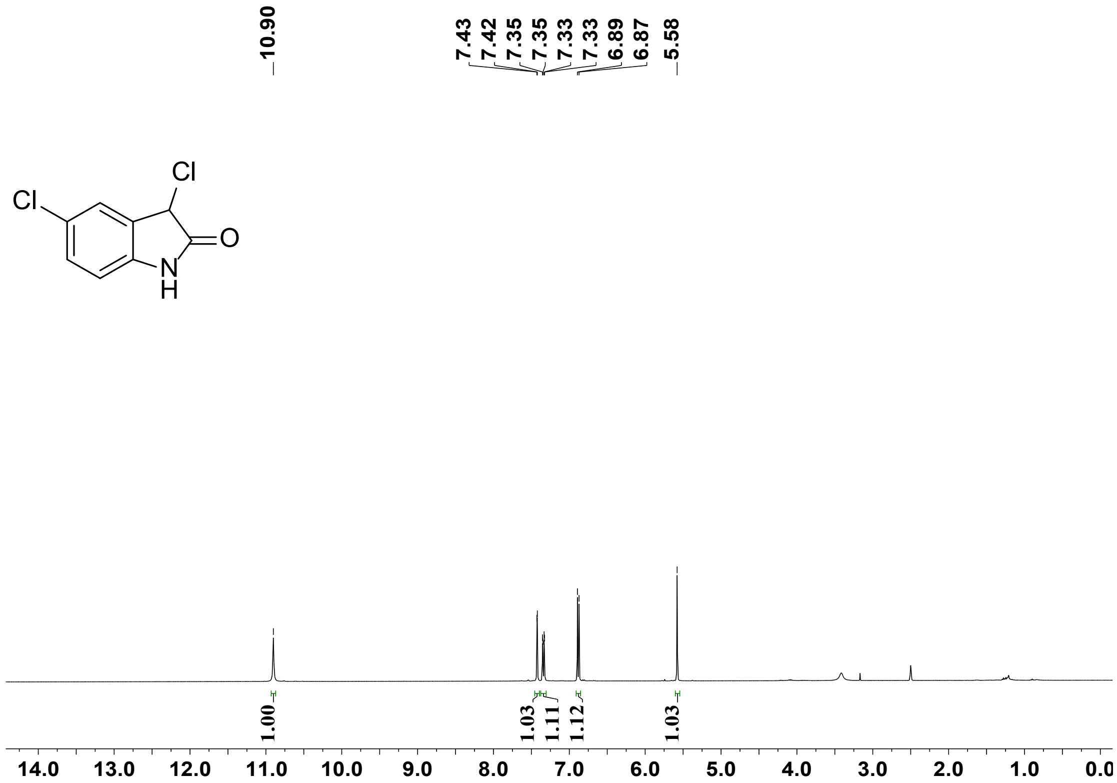

^{1}H NMR spectrum (400 MHz, DMSO-d_6) of **3e**

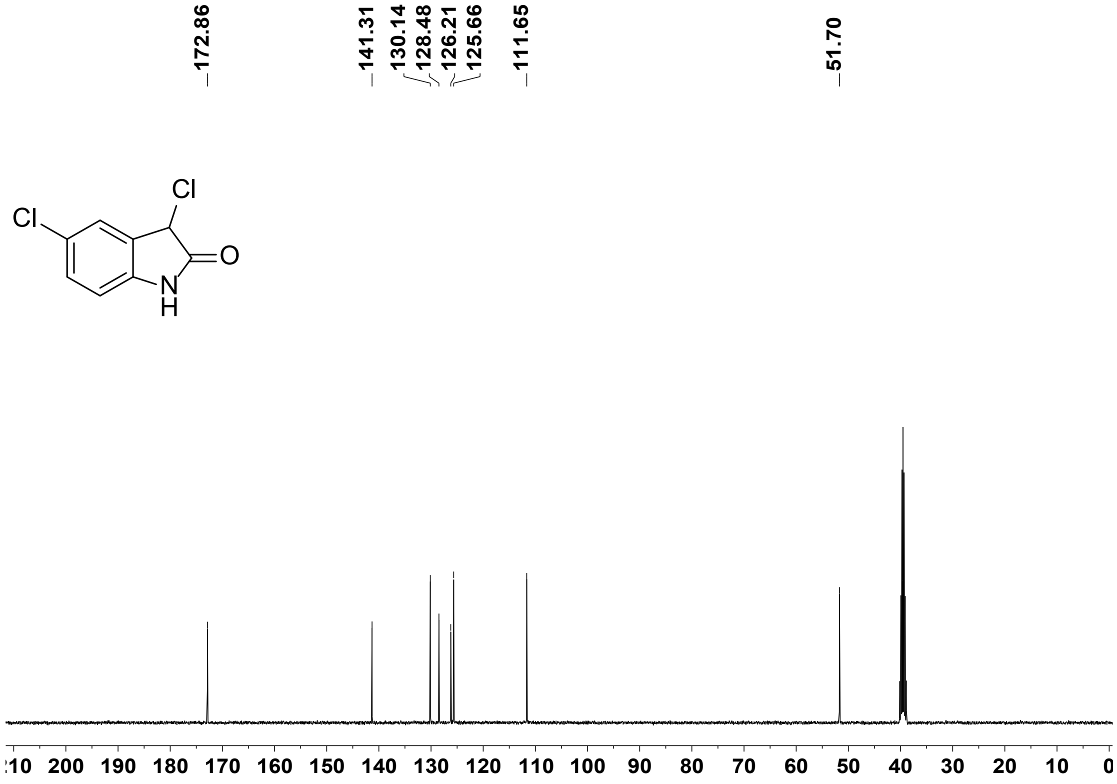

^{13}C NMR spectrum (100 MHz, DMSO-d_6) of **3e**

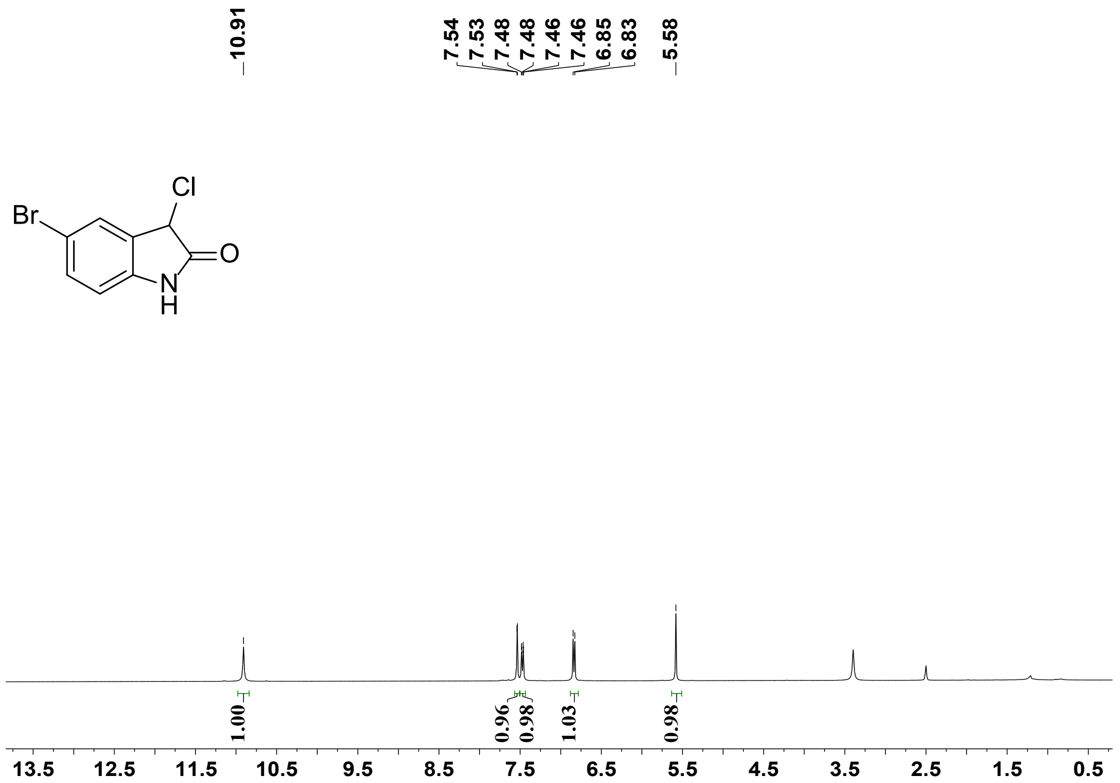

^{1}H NMR spectrum (400 MHz, DMSO-d_6) of **3f**

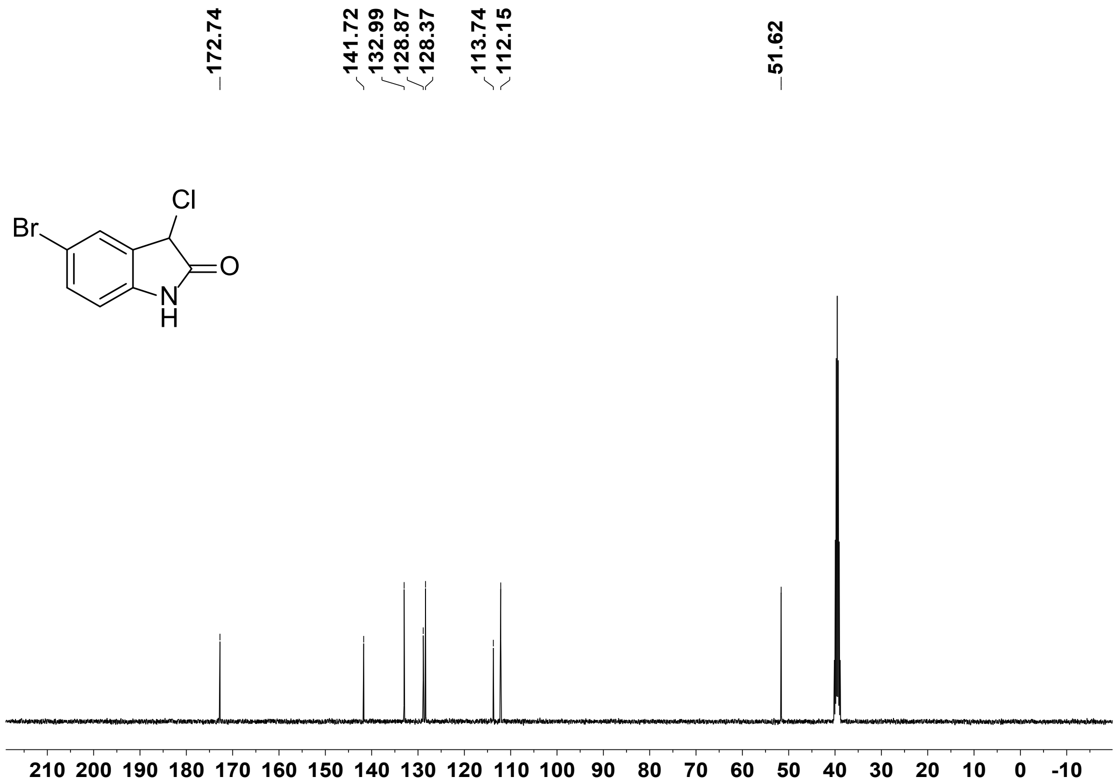

^{13}C NMR spectrum (100 MHz, DMSO-d_6) of **3f**

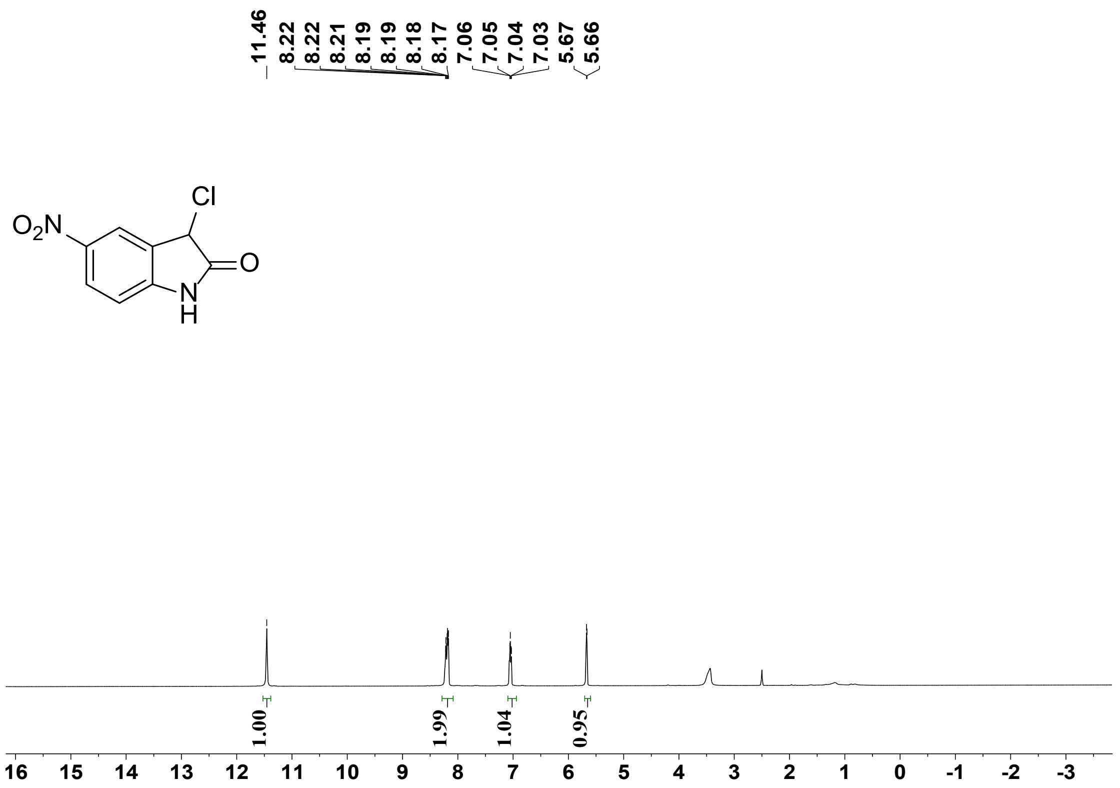

^{1}H NMR spectrum (400 MHz, DMSO-d_6) of **3g**

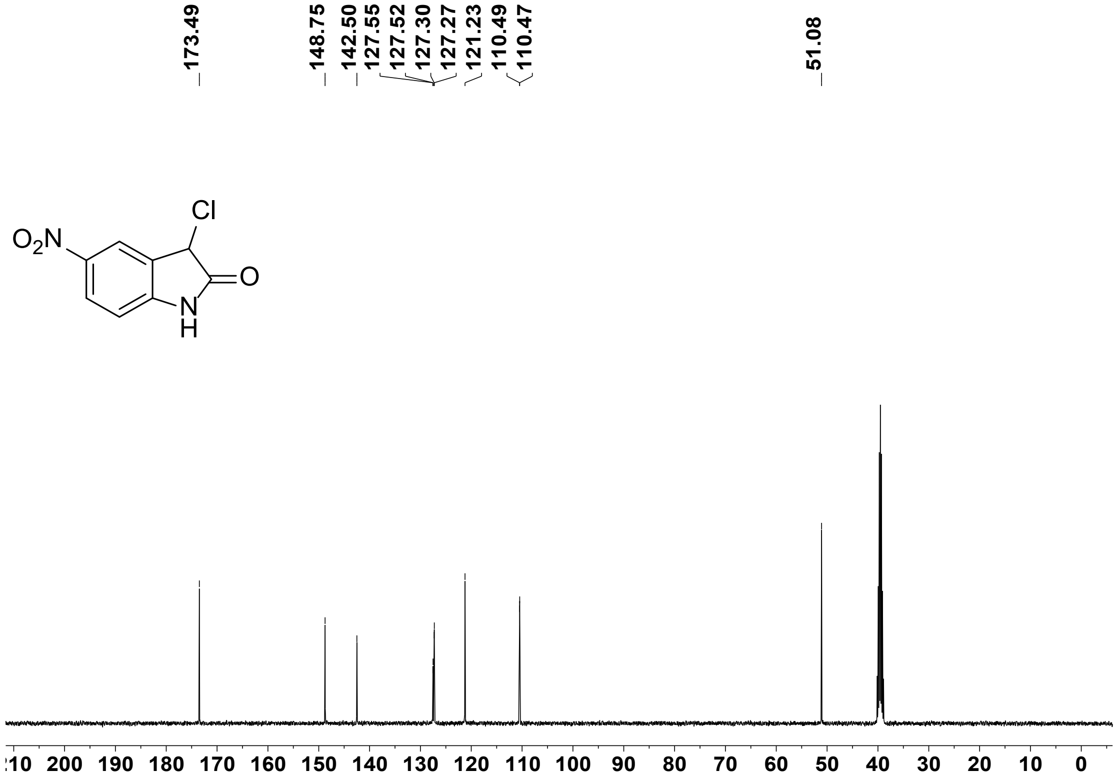

^{13}C NMR spectrum (100 MHz, DMSO-d_6) of **3g**

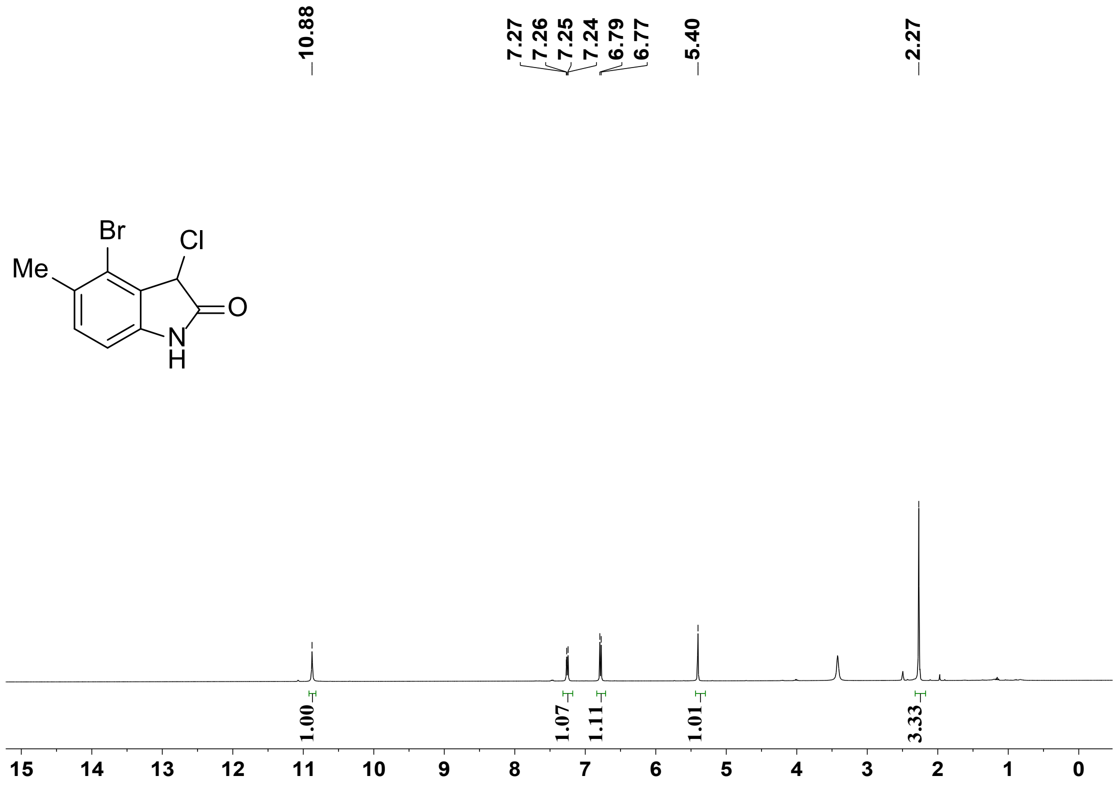

^{1}**H NMR** spectrum (400 MHz, DMSO-d_6) of **3h**

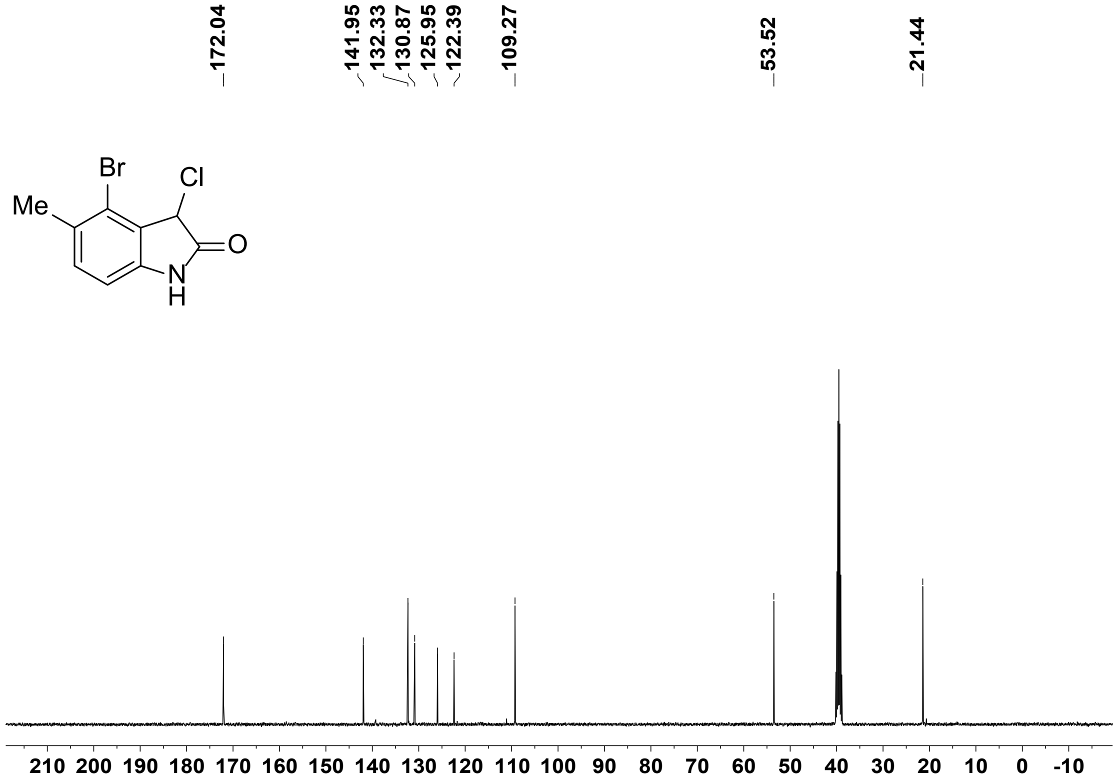

^{13}**C NMR** spectrum (100 MHz, DMSO-d_6) of **3h**

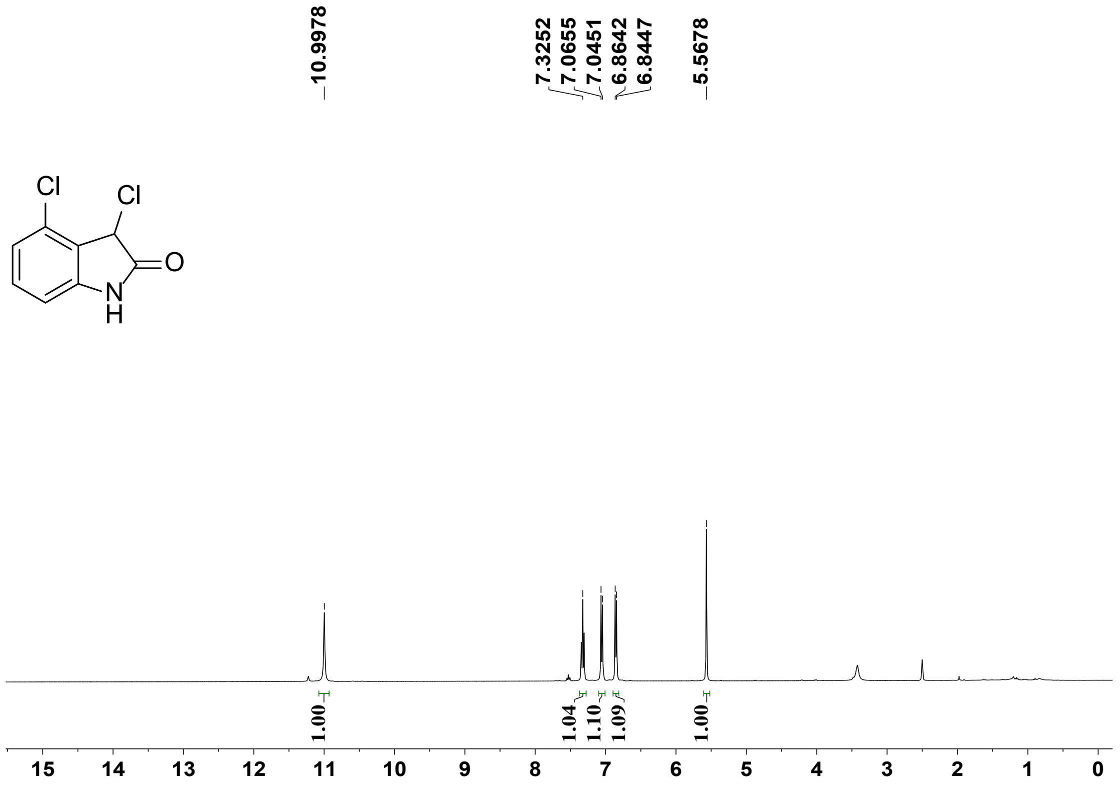

^{1}H NMR spectrum (400 MHz, DMSO-d_6) of **3i**

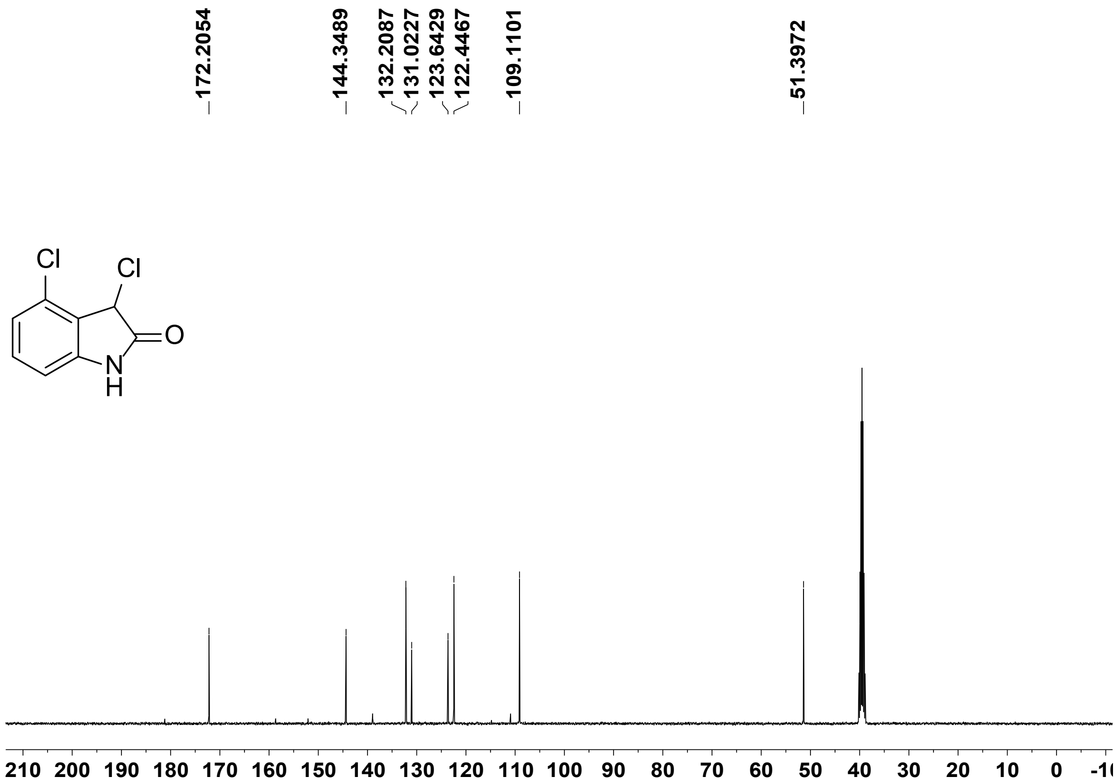

^{13}C NMR spectrum (100 MHz, DMSO-d_6) of **3i**

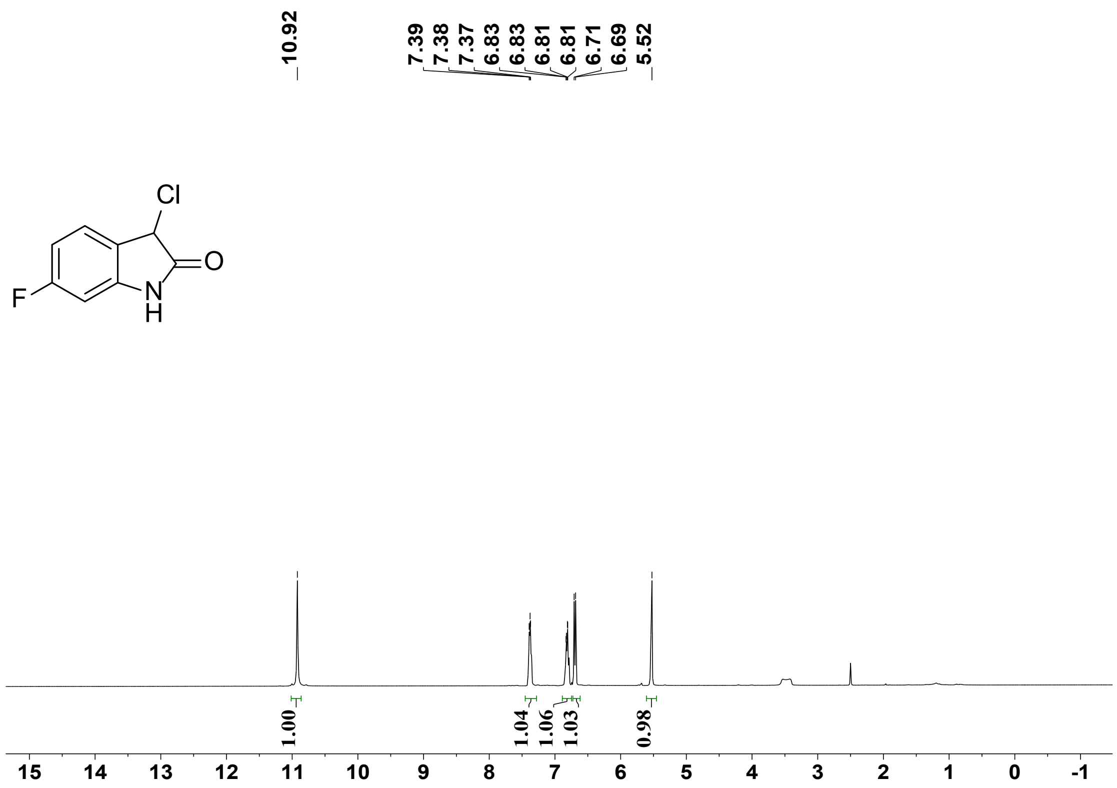

^{1}H NMR spectrum (400 MHz, DMSO-d_6) of **3j**

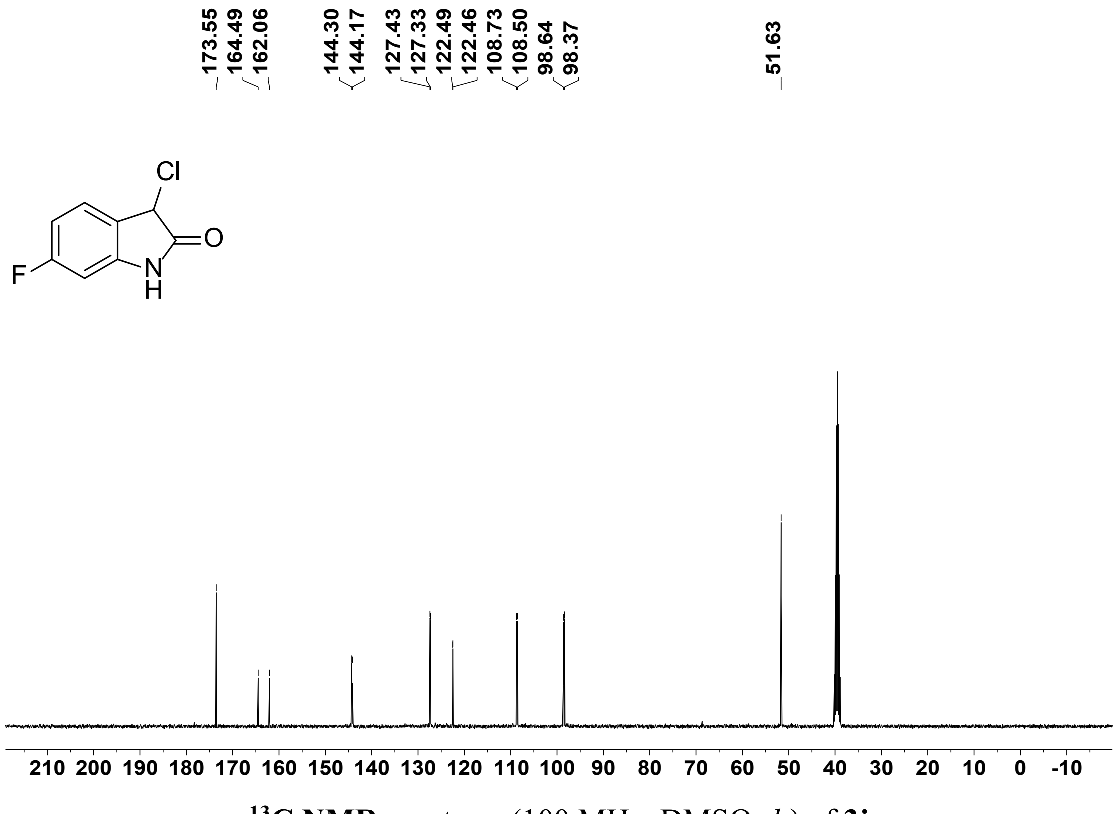

^{13}C NMR spectrum (100 MHz, DMSO-d_6) of **3j**

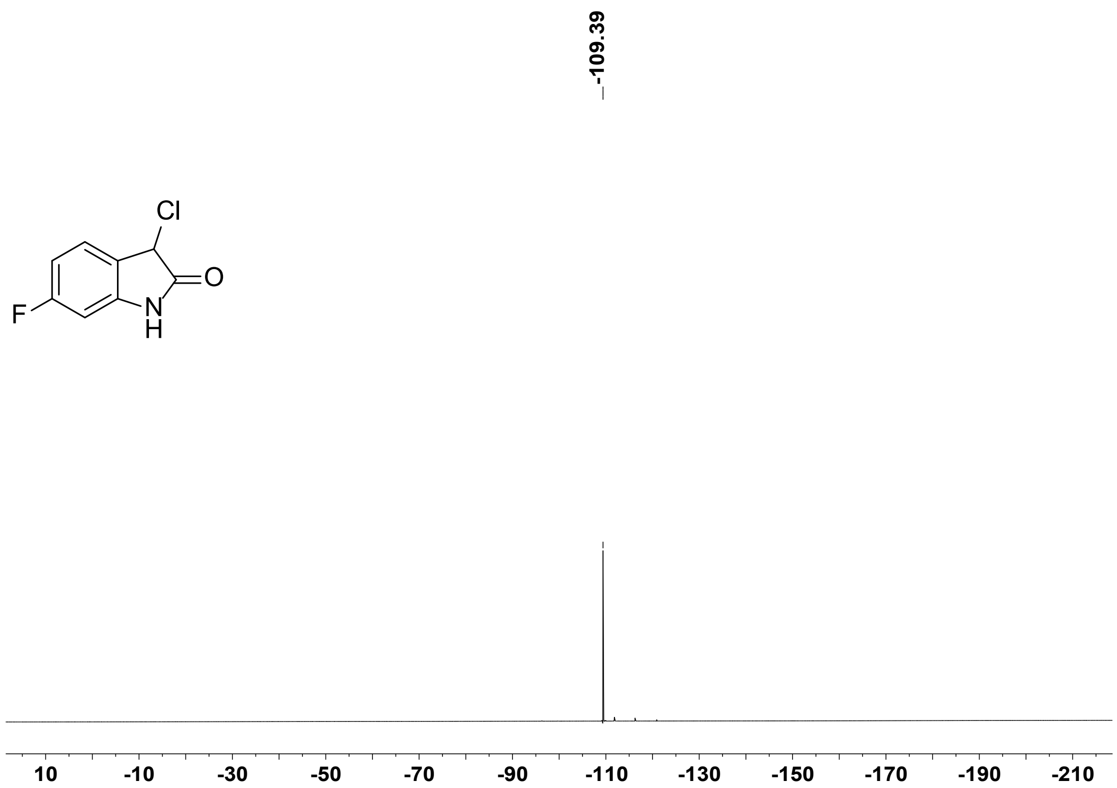

^{19}F NMR spectrum (377 MHz, DMSO-d_6) of **3j**

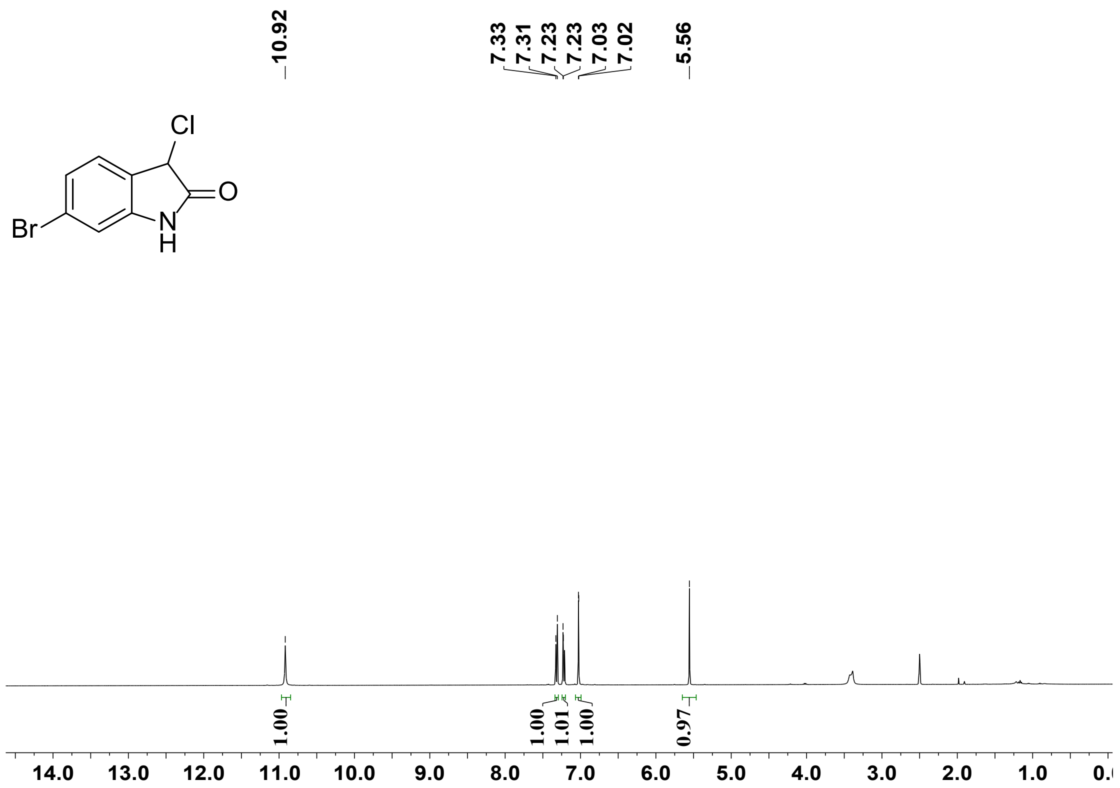

¹H NMR spectrum (400 MHz, DMSO-d_6) of **3k**

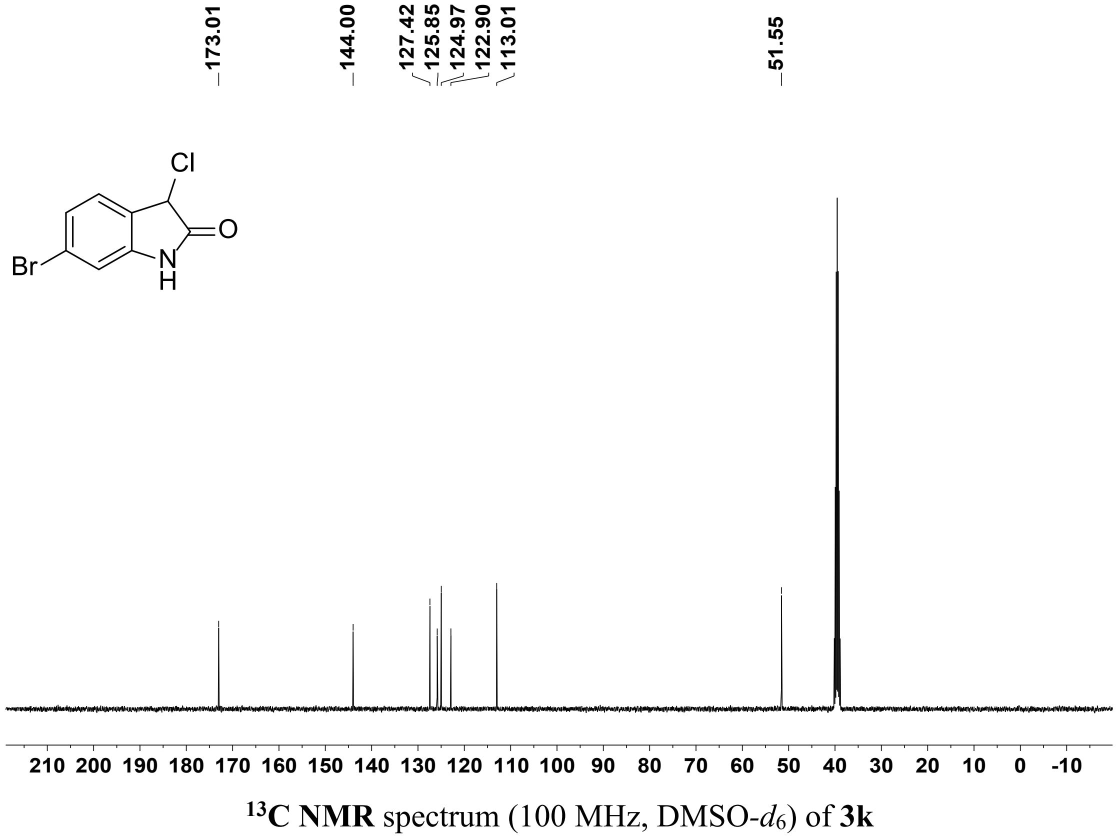

¹³C NMR spectrum (100 MHz, DMSO-d_6) of **3k**

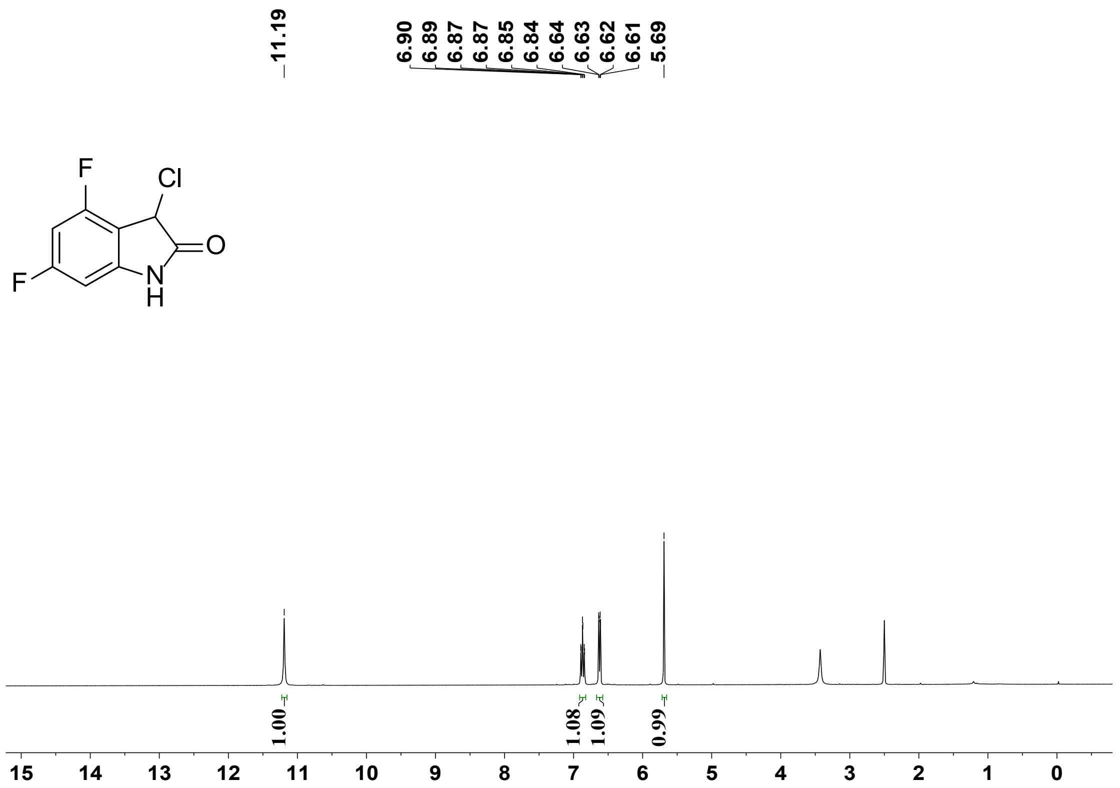

^{1}H NMR spectrum (400 MHz, DMSO-d_6) of **3l**

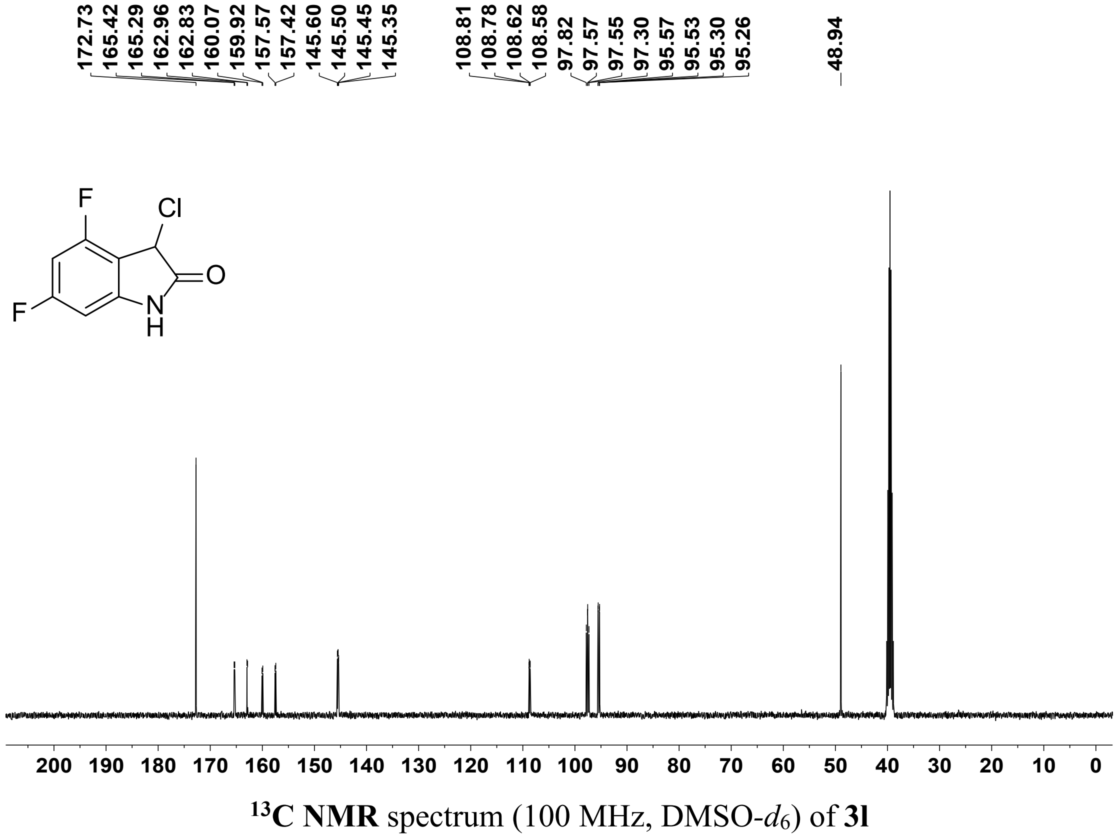

^{13}C NMR spectrum (100 MHz, DMSO-d_6) of **3l**

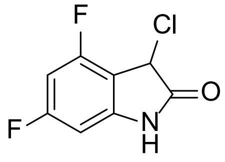

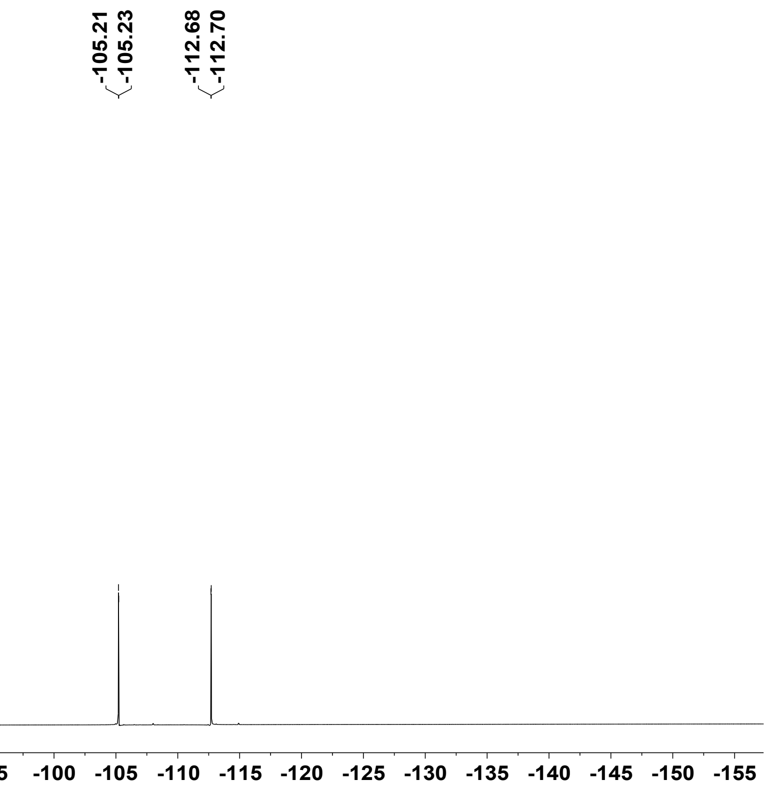

^{19}F NMR spectrum (377 MHz, DMSO-d_6) of **3l**

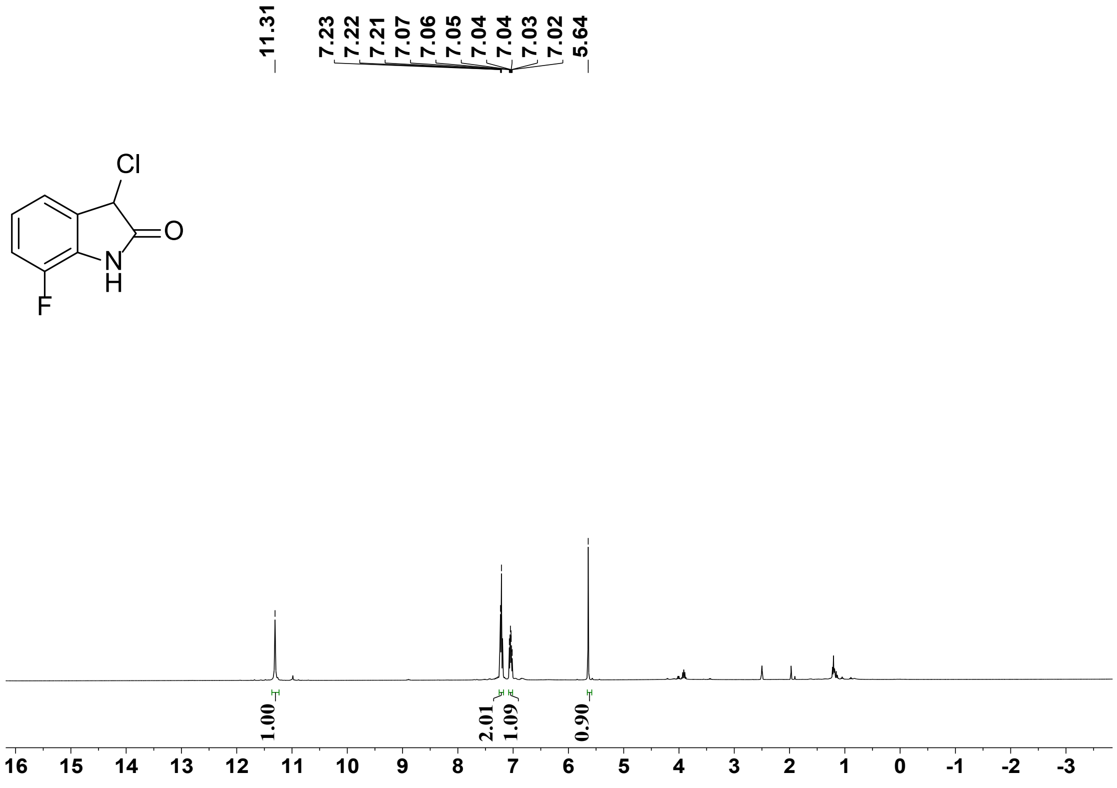

^{1}H NMR spectrum (400 MHz, DMSO-d_6) of **3m**

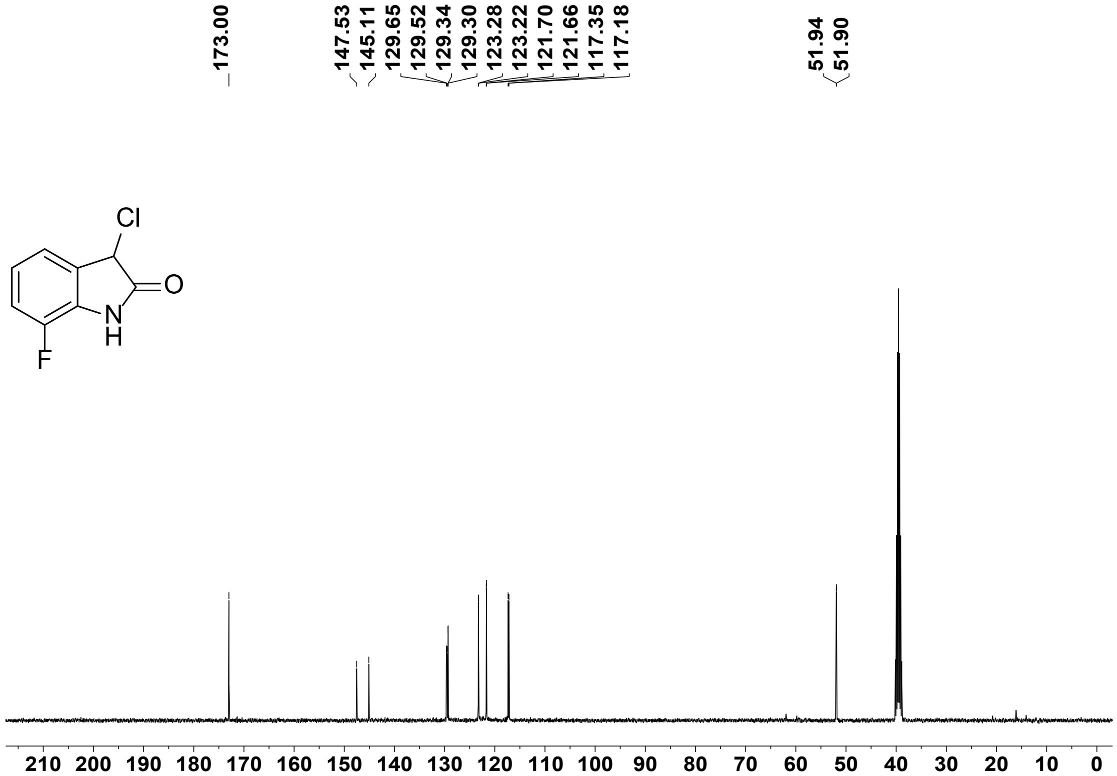

^{13}C NMR spectrum (100 MHz, DMSO-d_6) of **3m**

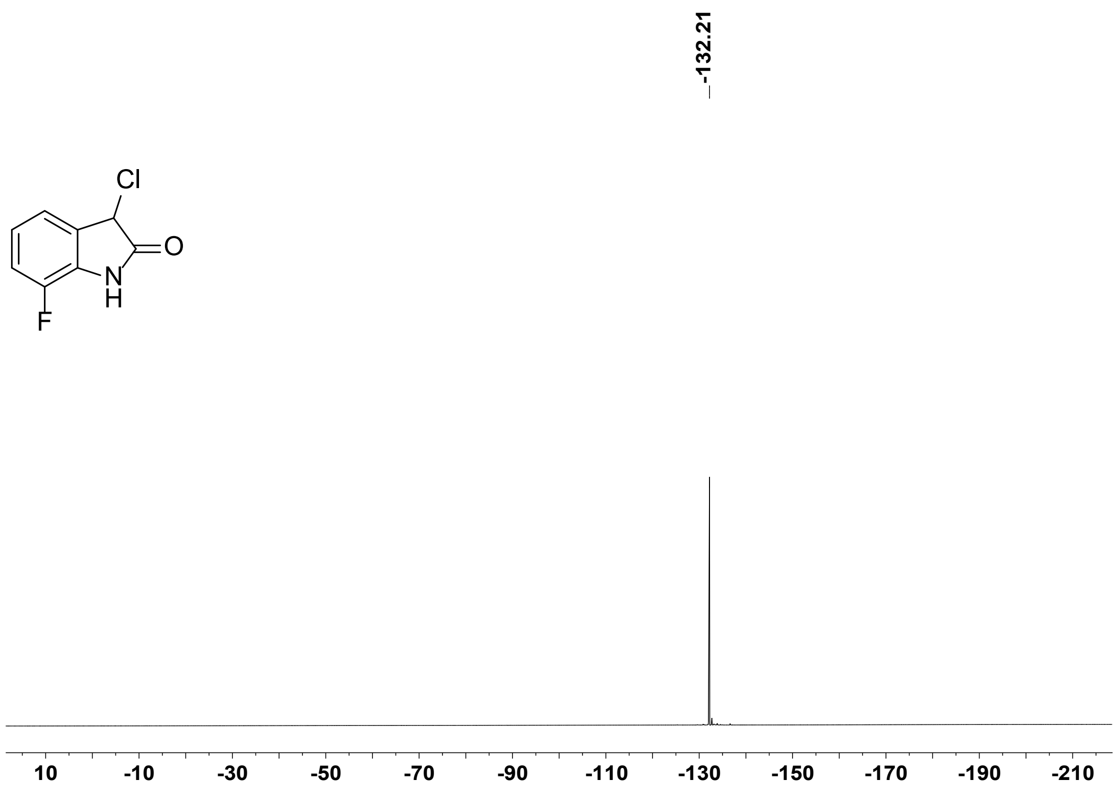

^{19}F NMR spectrum (377 MHz, DMSO-d_6) of **3m**

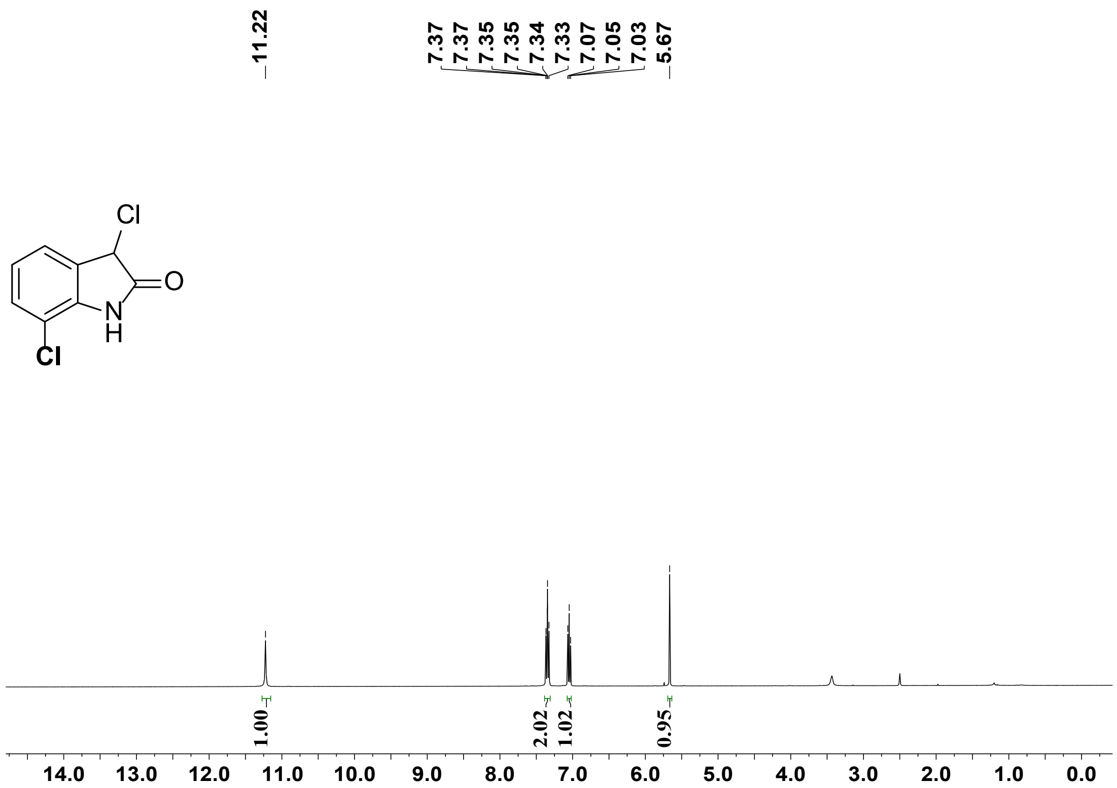

^{1}H NMR spectrum (400 MHz, DMSO-d_6) of **3n**

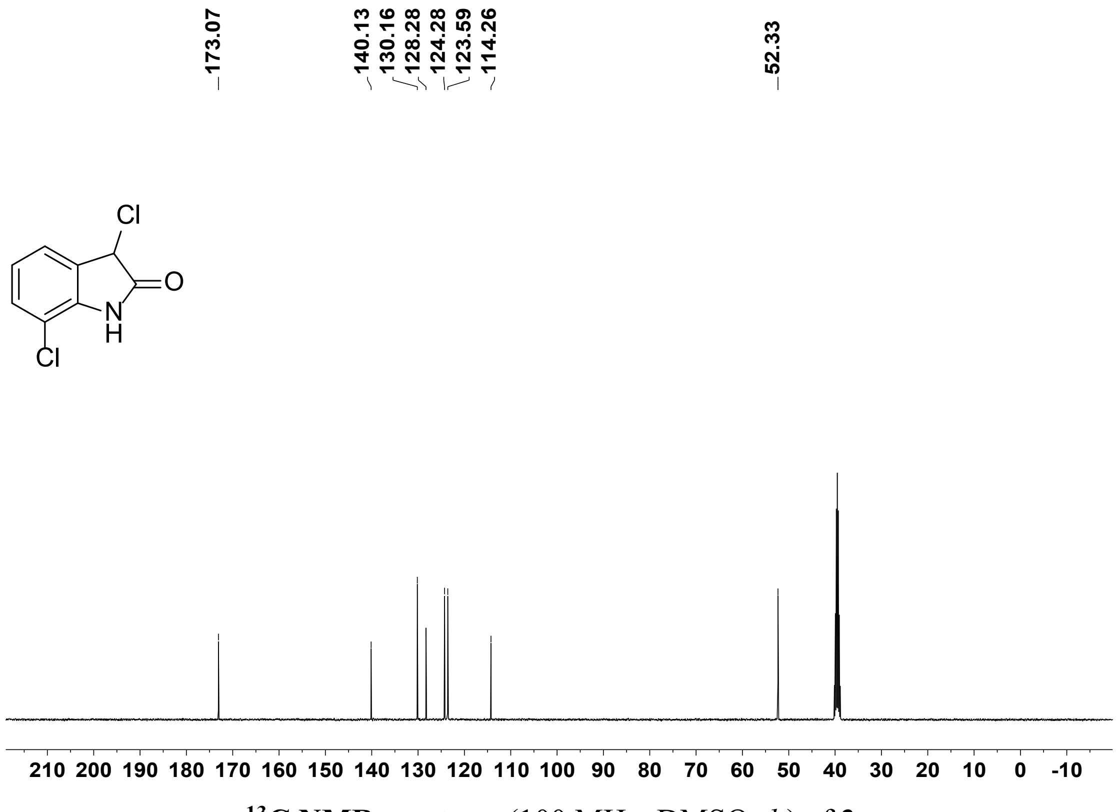

^{13}C NMR spectrum (100 MHz, DMSO-d_6) of **3n**

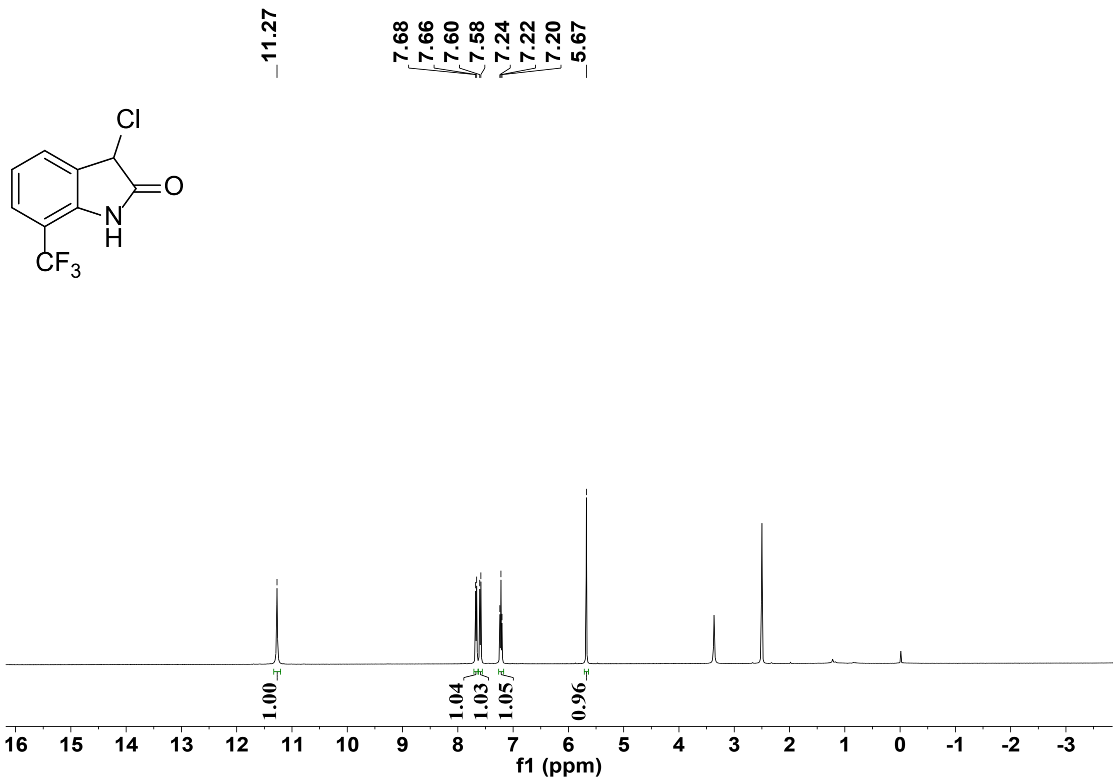

^{1}H NMR spectrum (400 MHz, DMSO-d_6) of **3o**

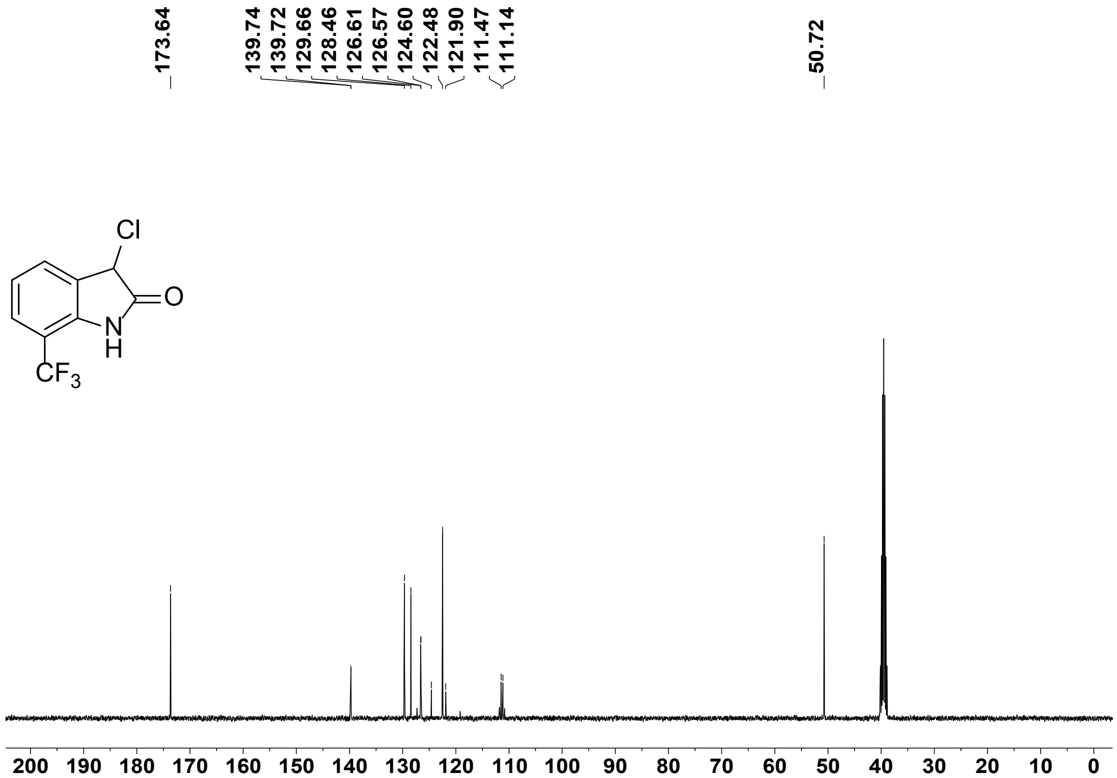

^{13}C NMR spectrum (100 MHz, DMSO-d_6) of **3o**

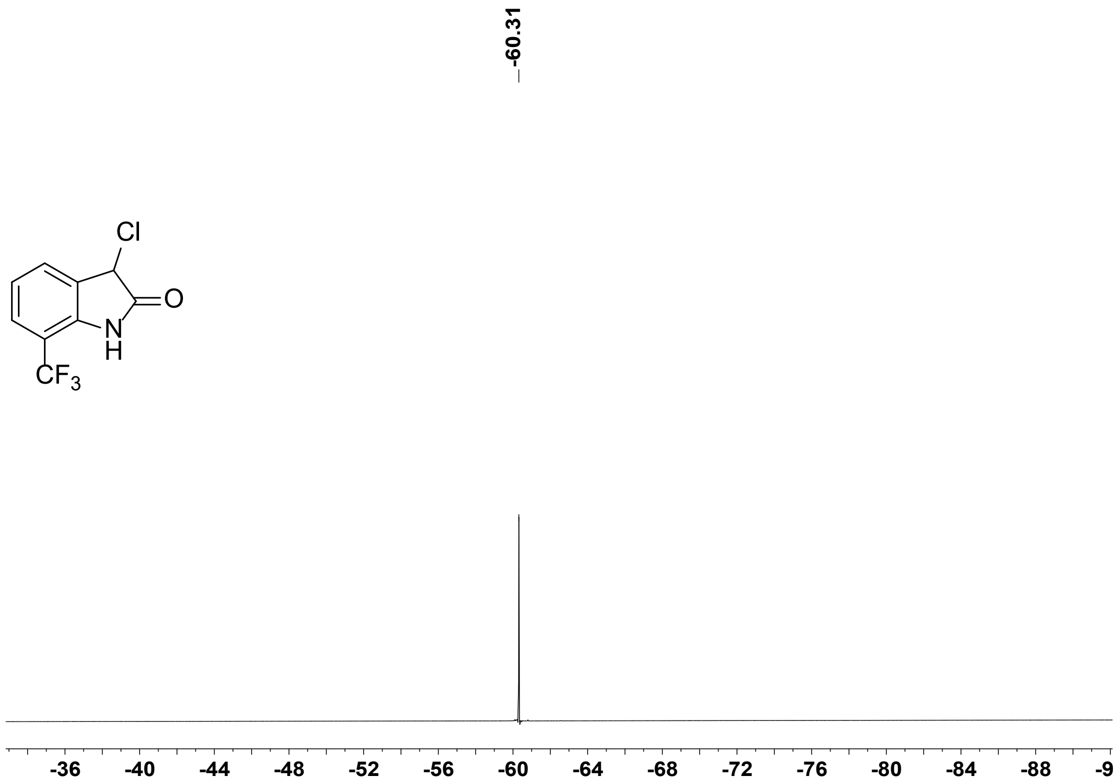

^{19}**F NMR** spectrum (377 MHz, DMSO-d_6) of **3o**

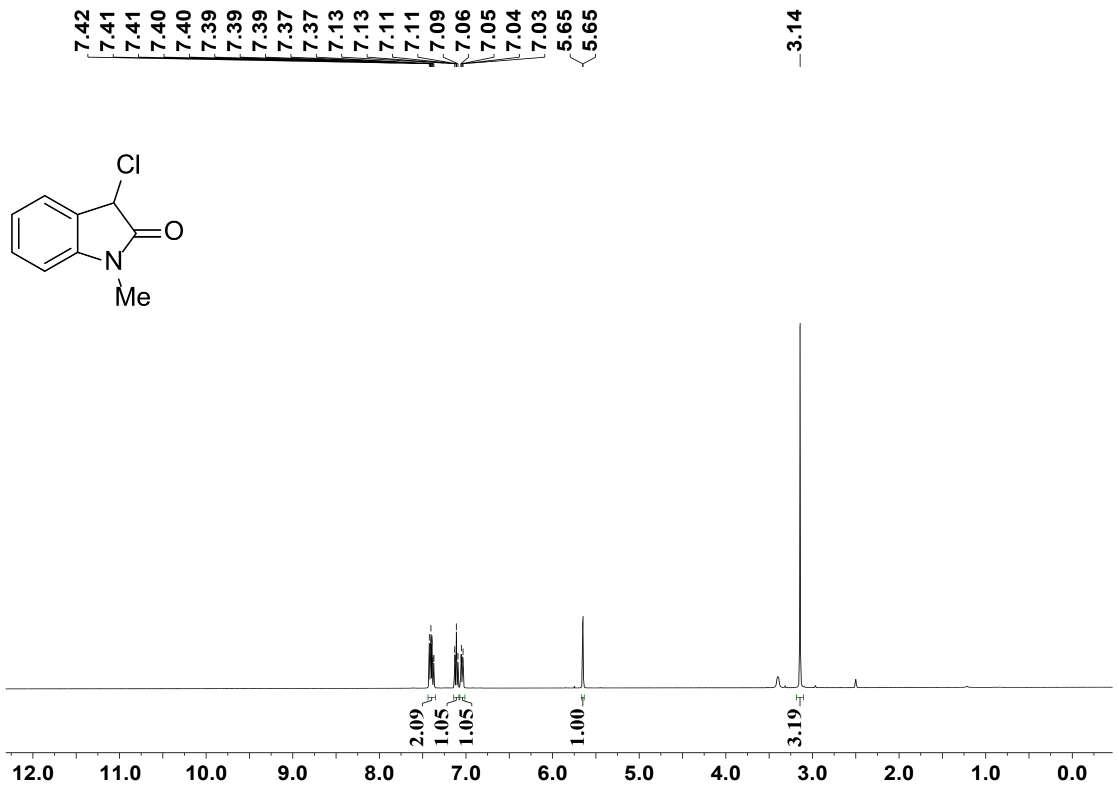

^{1}H NMR spectrum (400 MHz, DMSO-d_6) of **3p**

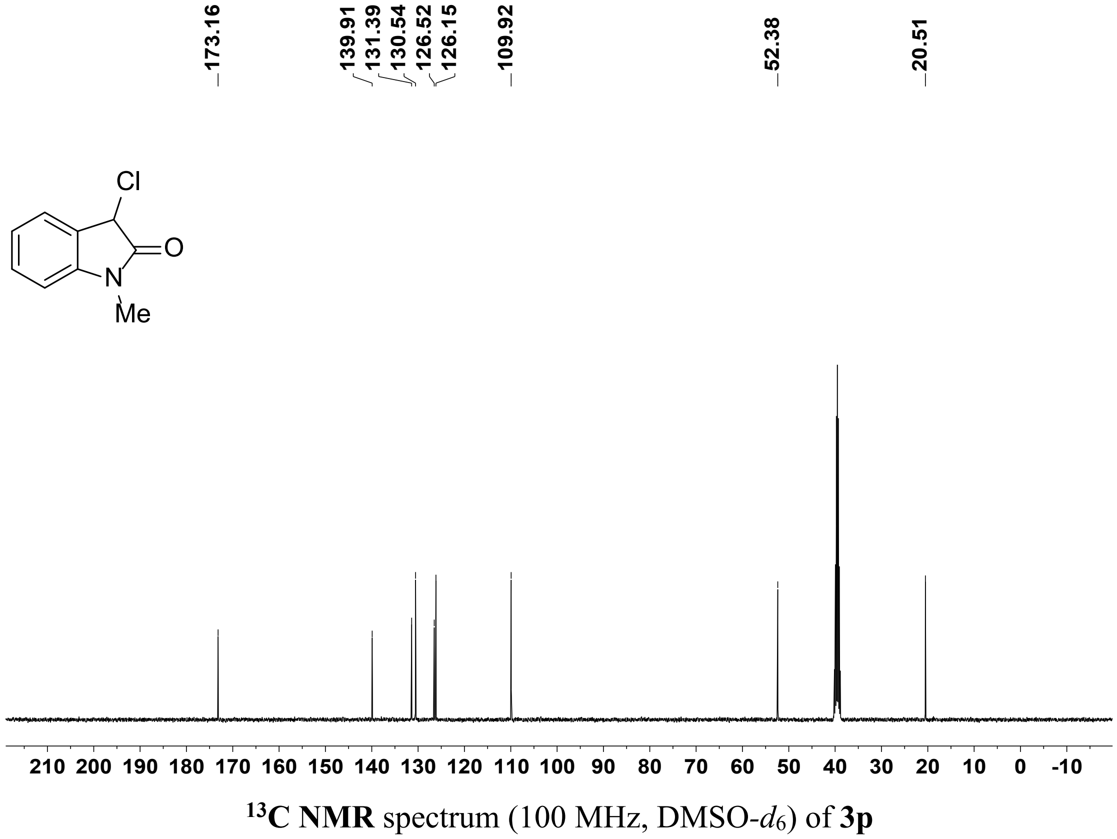

^{13}C NMR spectrum (100 MHz, DMSO-d_6) of **3p**

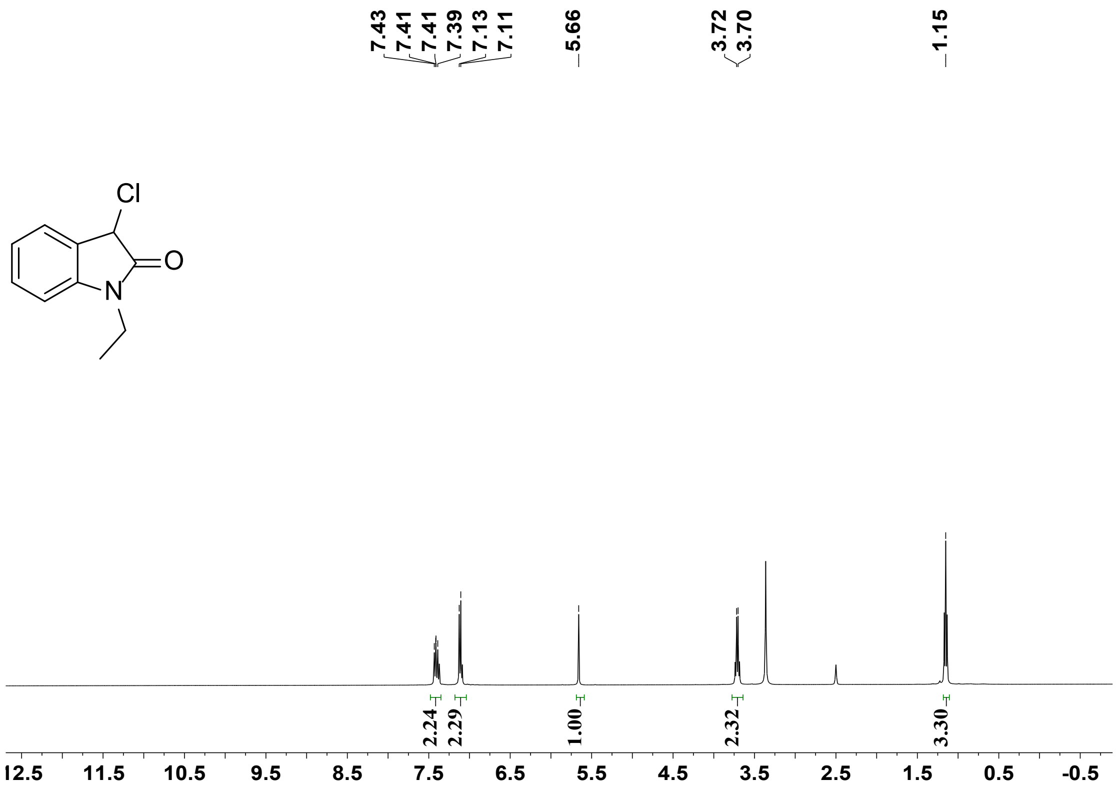

^{1}H NMR spectrum (400 MHz, DMSO-d_6) of **3q**

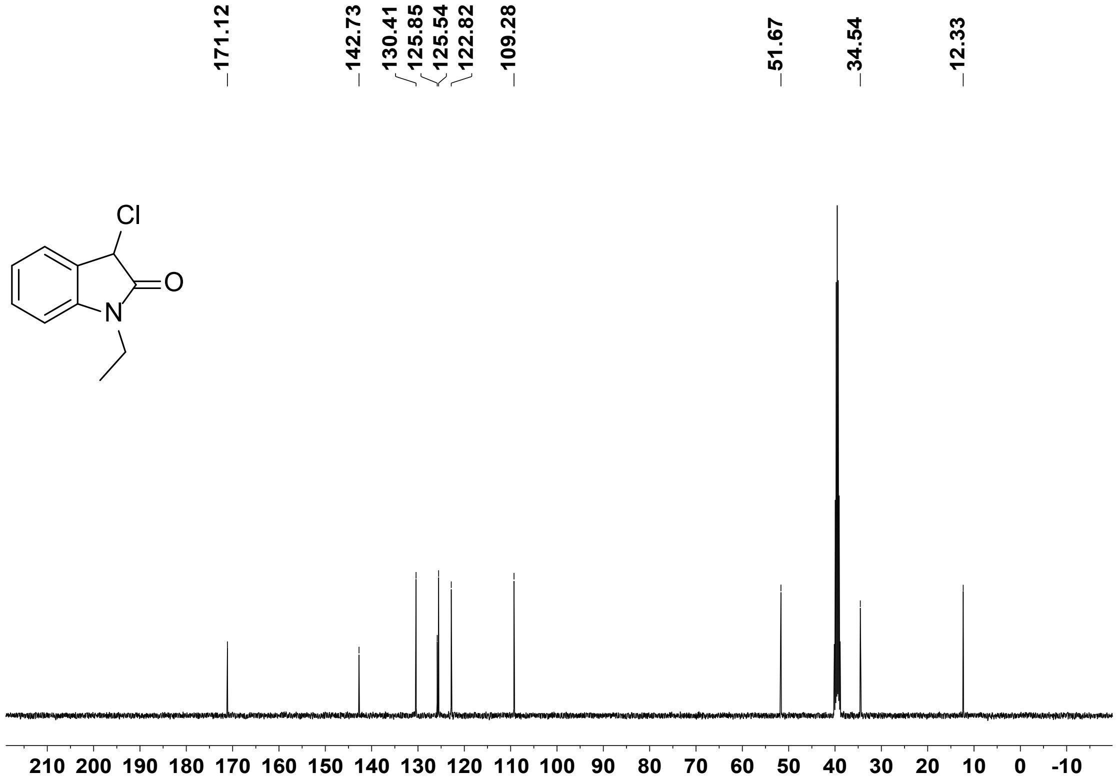

^{13}C NMR spectrum (100 MHz, DMSO-d_6) of **3q**

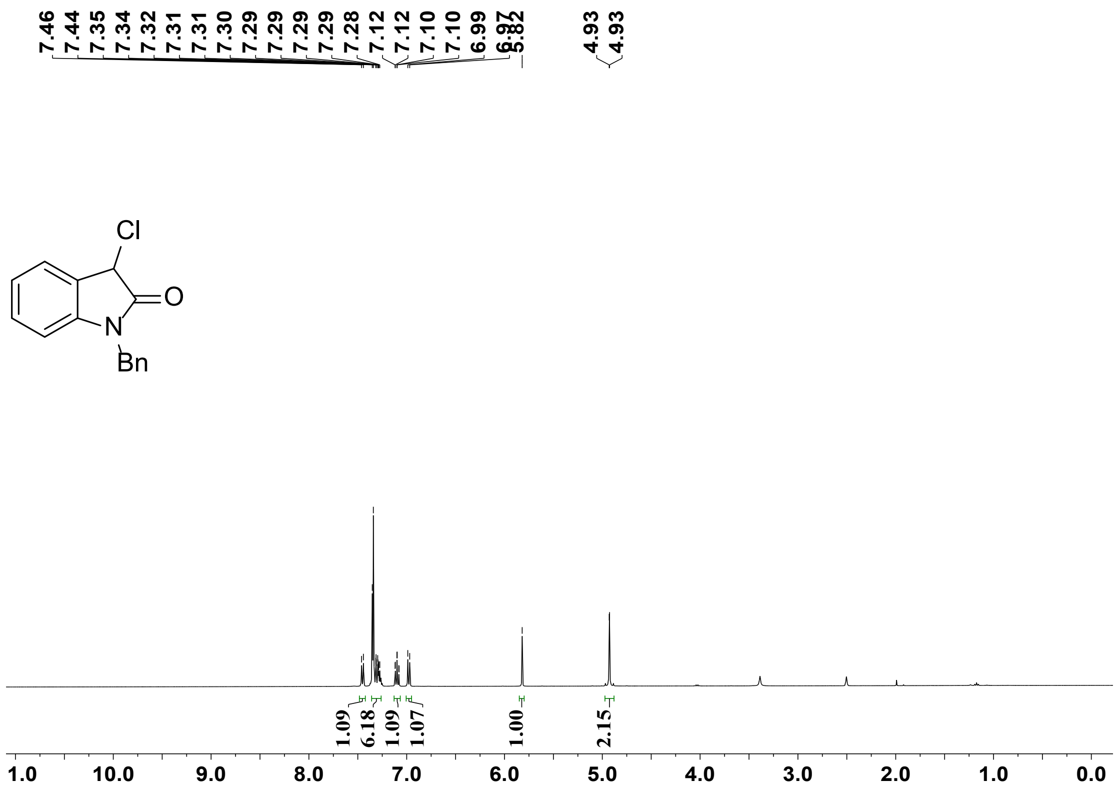

^{1}H NMR spectrum (400 MHz, DMSO-d_6) of **3r**

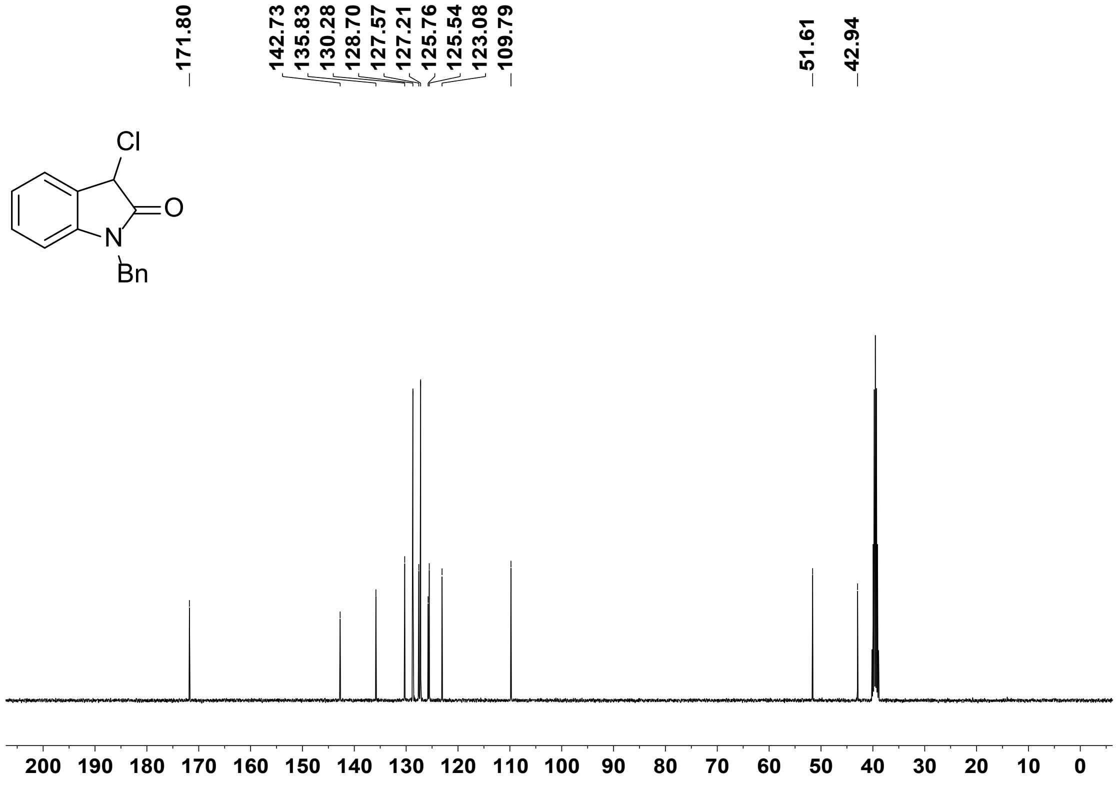

^{13}C NMR spectrum (100 MHz, DMSO-d_6) of **3r**

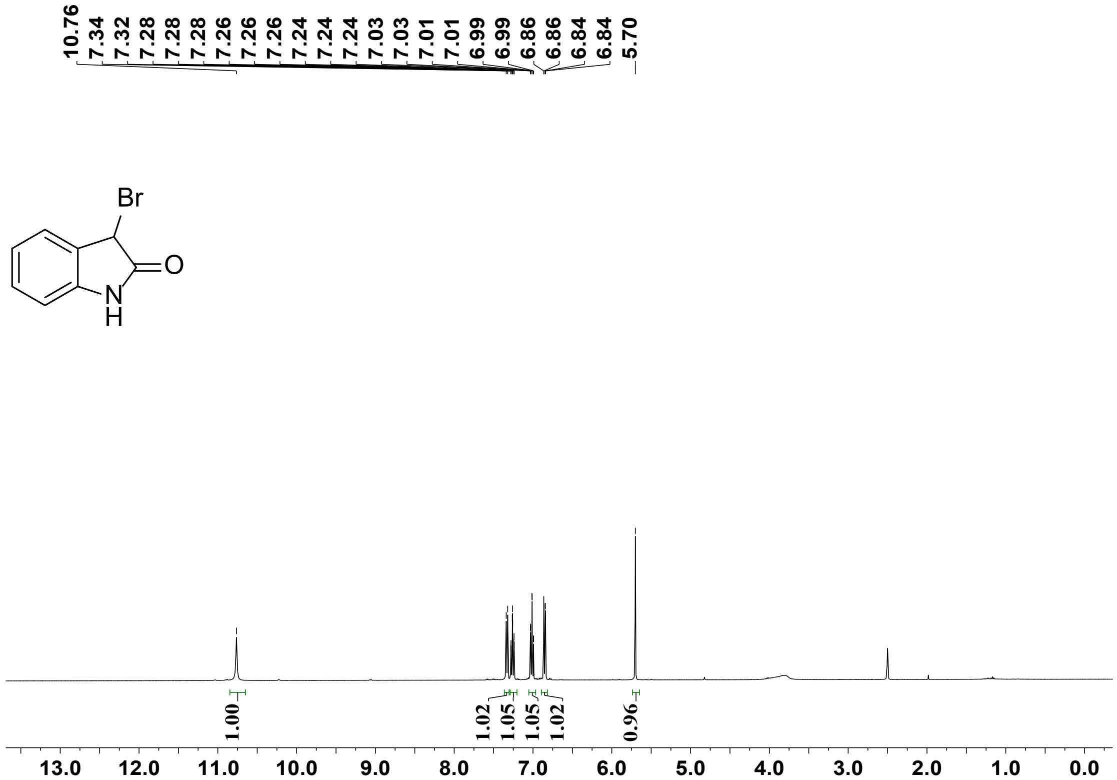

^{1}H NMR spectrum (400 MHz, DMSO-d_6) of **4a**

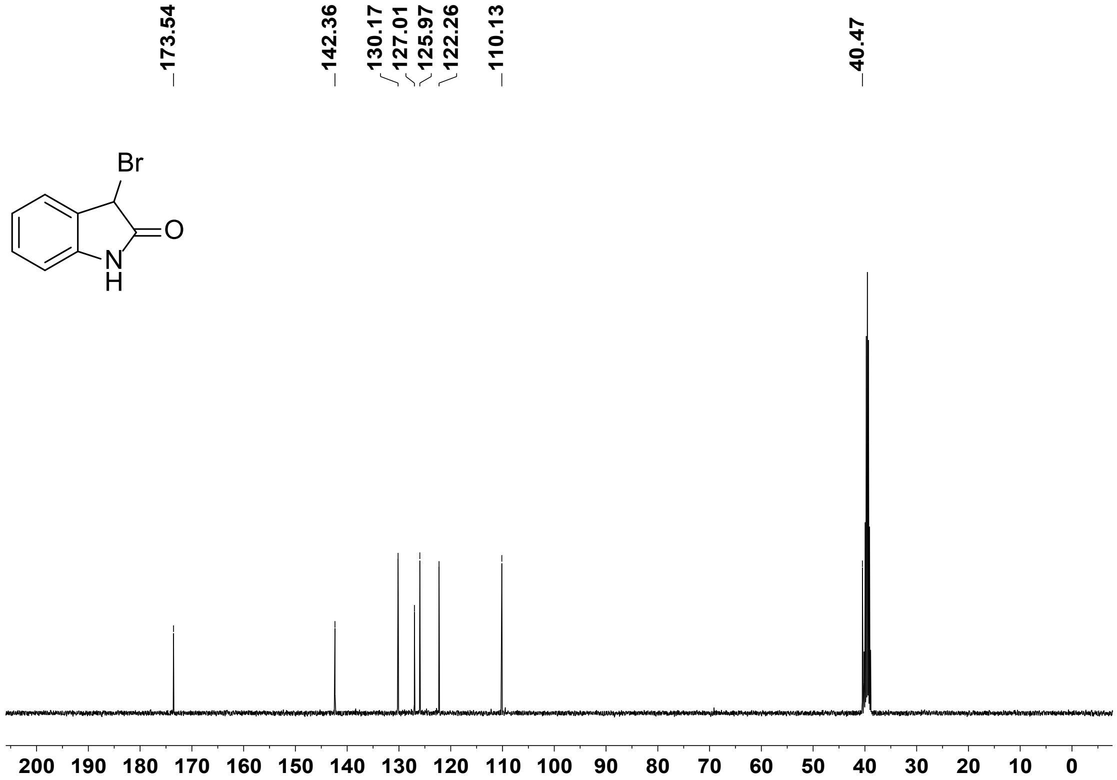

^{13}C NMR spectrum (100 MHz, DMSO-d_6) of **4a**

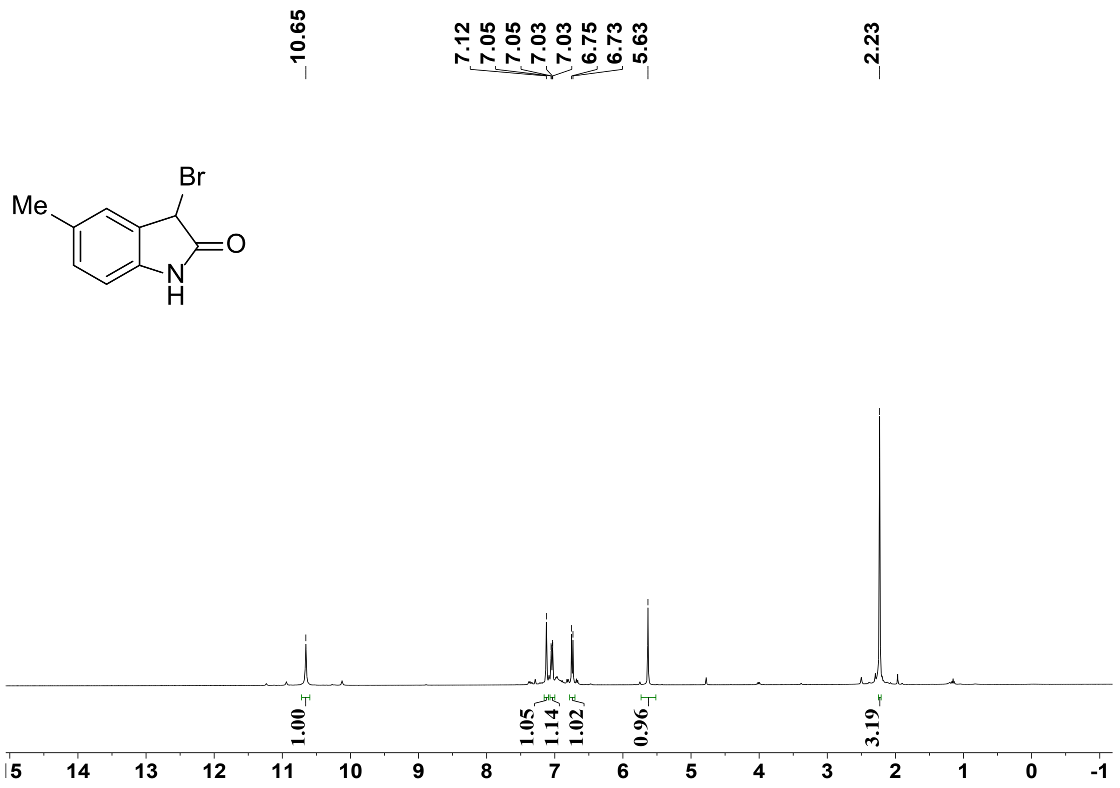

^{1}**H NMR** spectrum (400 MHz, DMSO-d_6) of **4b**

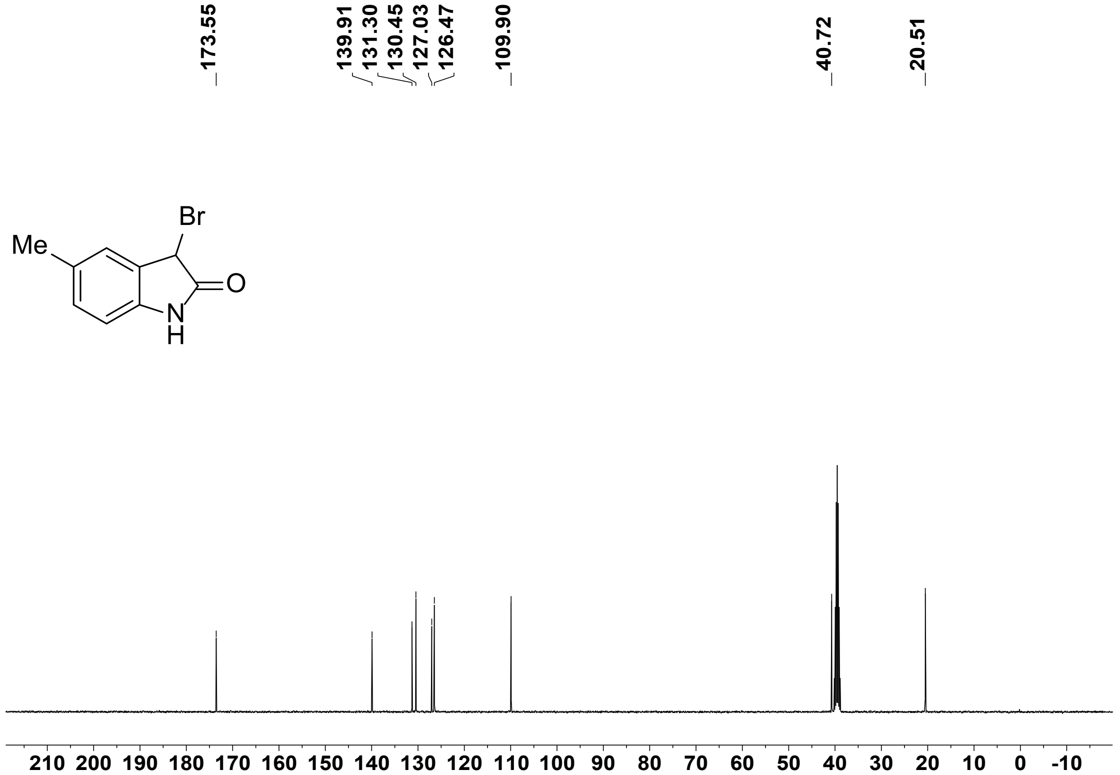

^{13}**C NMR** spectrum (100 MHz, DMSO-d_6) of **4b**

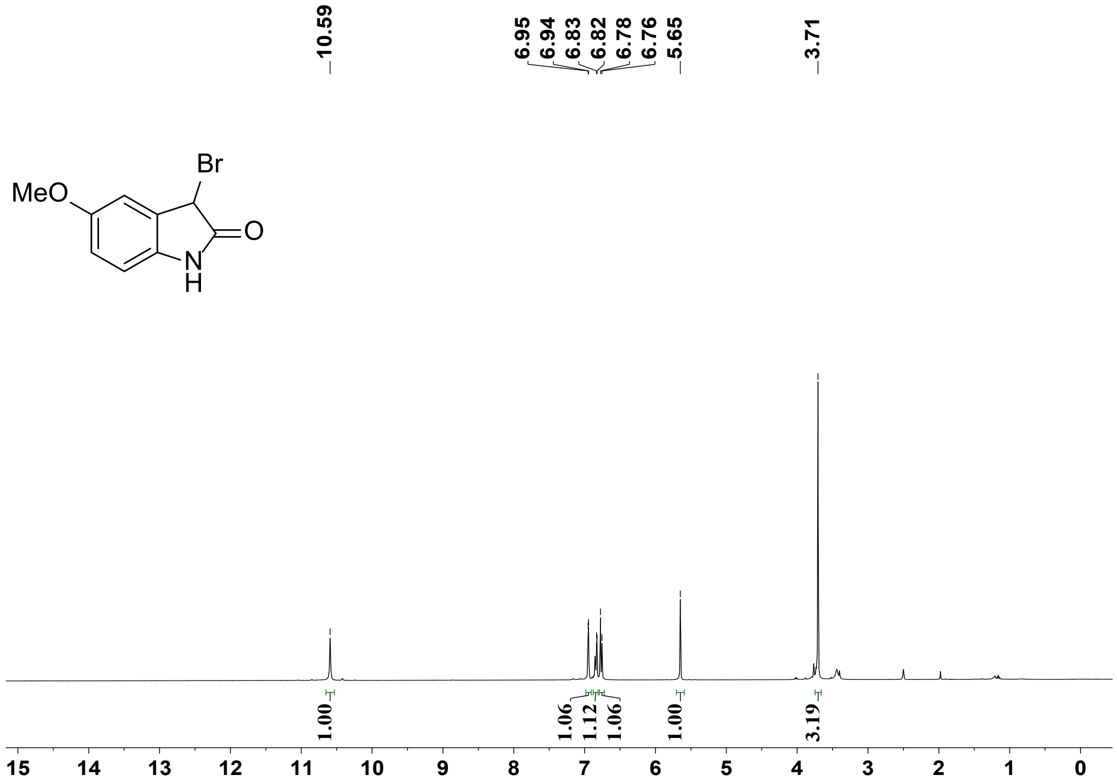

^{1}H NMR spectrum (400 MHz, DMSO-d_6) of **4c**

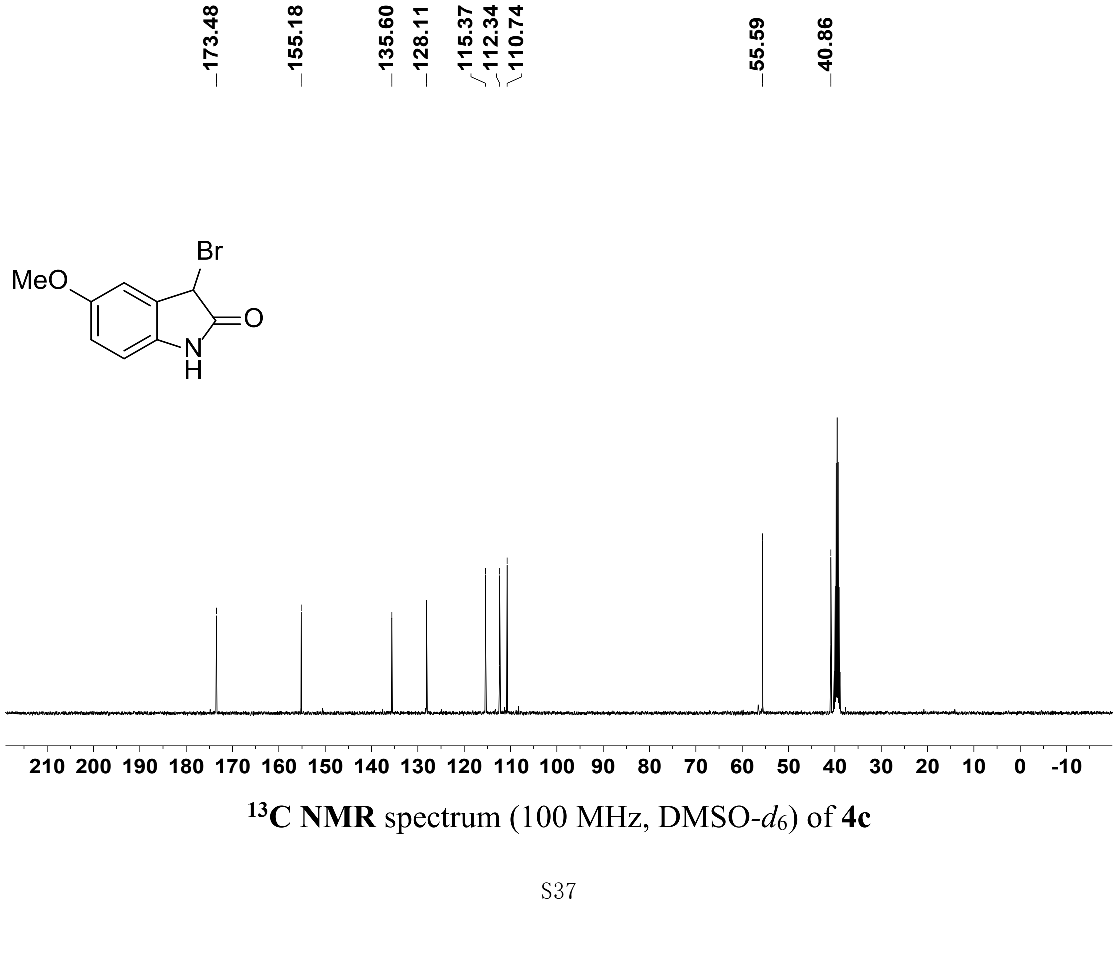

^{13}C NMR spectrum (100 MHz, DMSO-d_6) of **4c**

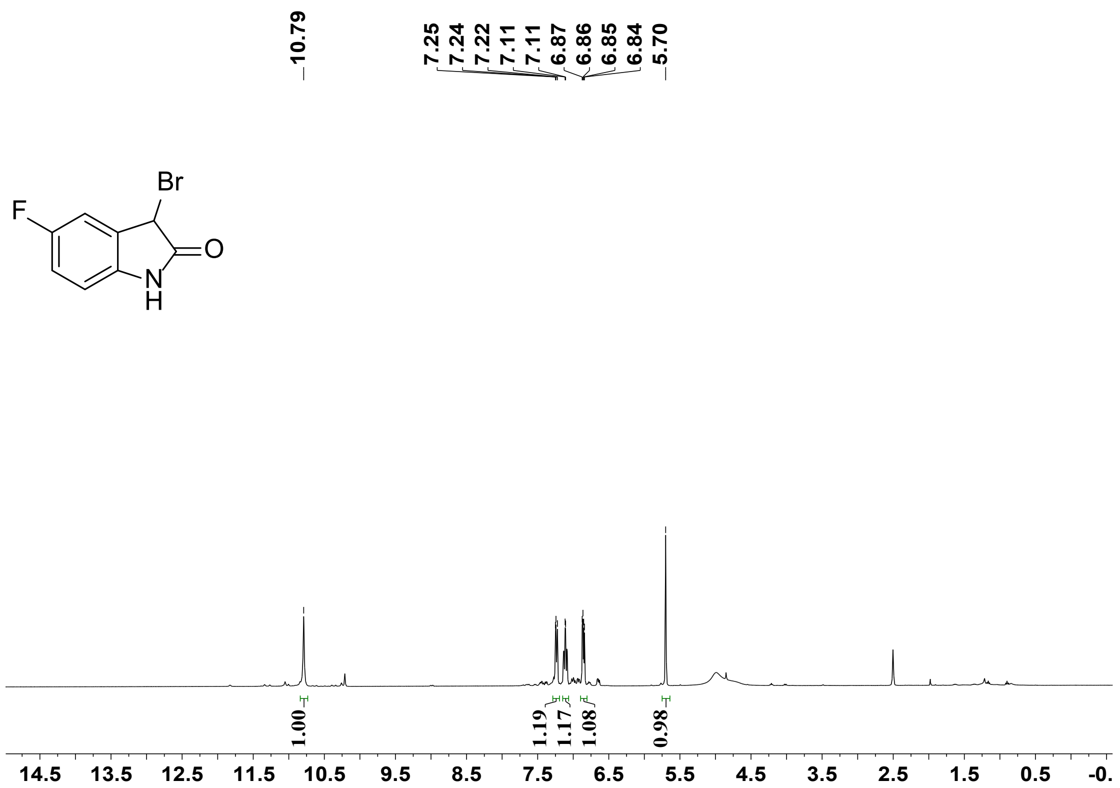

^{1}H NMR spectrum (400 MHz, DMSO-d_6) of **4d**

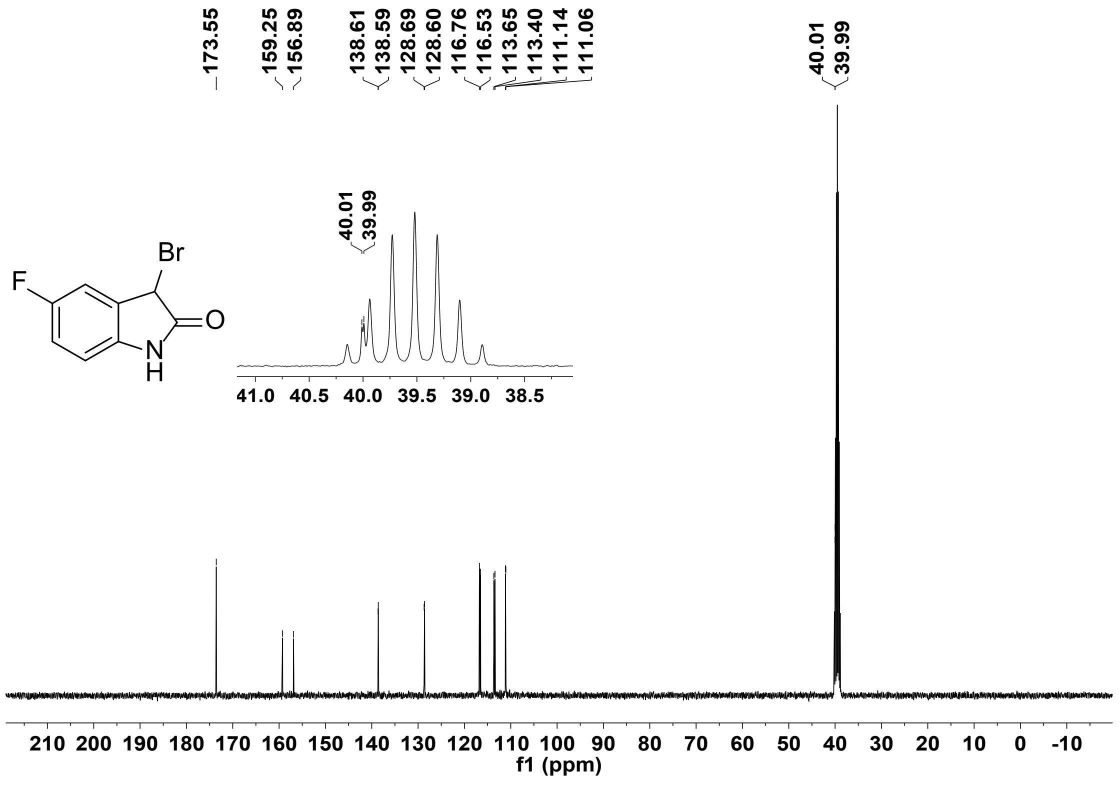

^{13}C NMR spectrum (100 MHz, DMSO-d_6) of **4d**

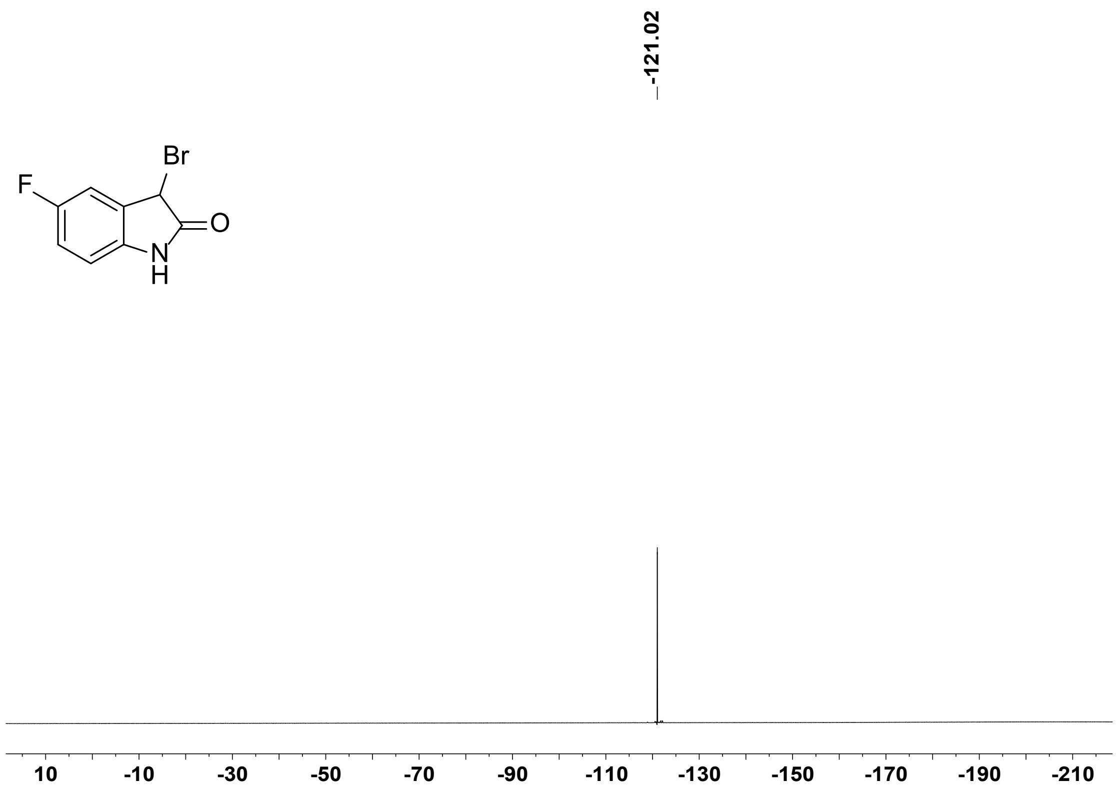

^{19}**F NMR** spectrum (377 MHz, DMSO-d_6) of **4d**

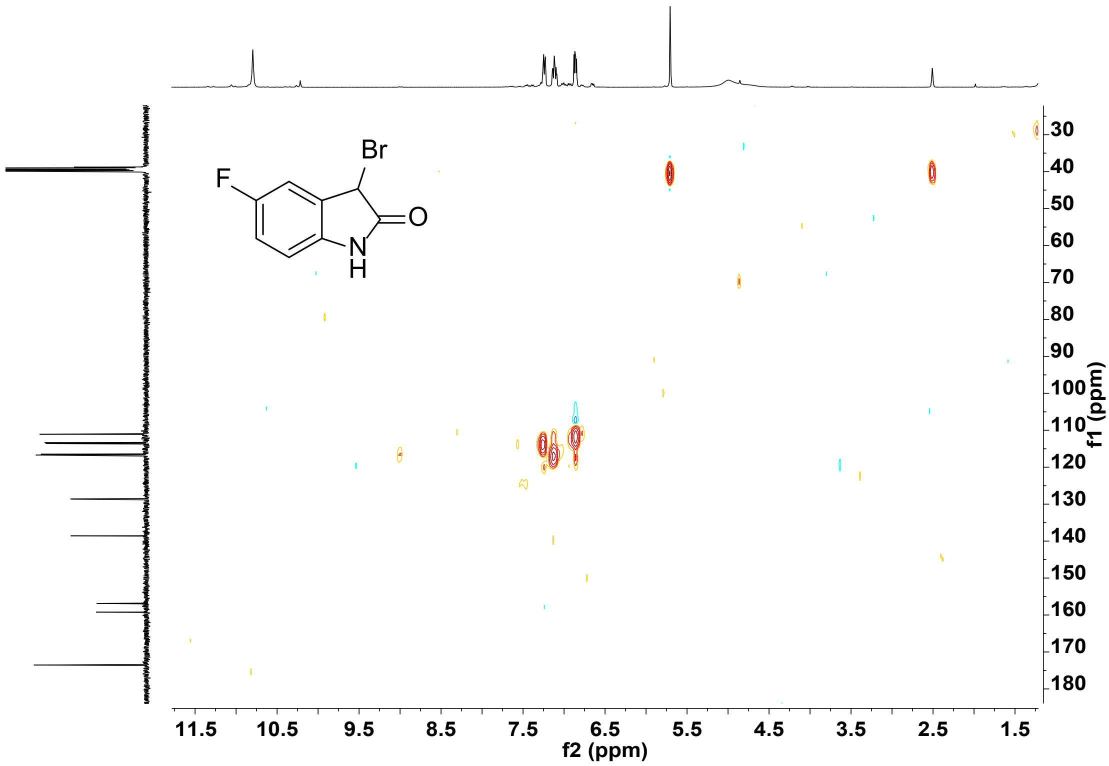

HSQC spectrum of **4d**

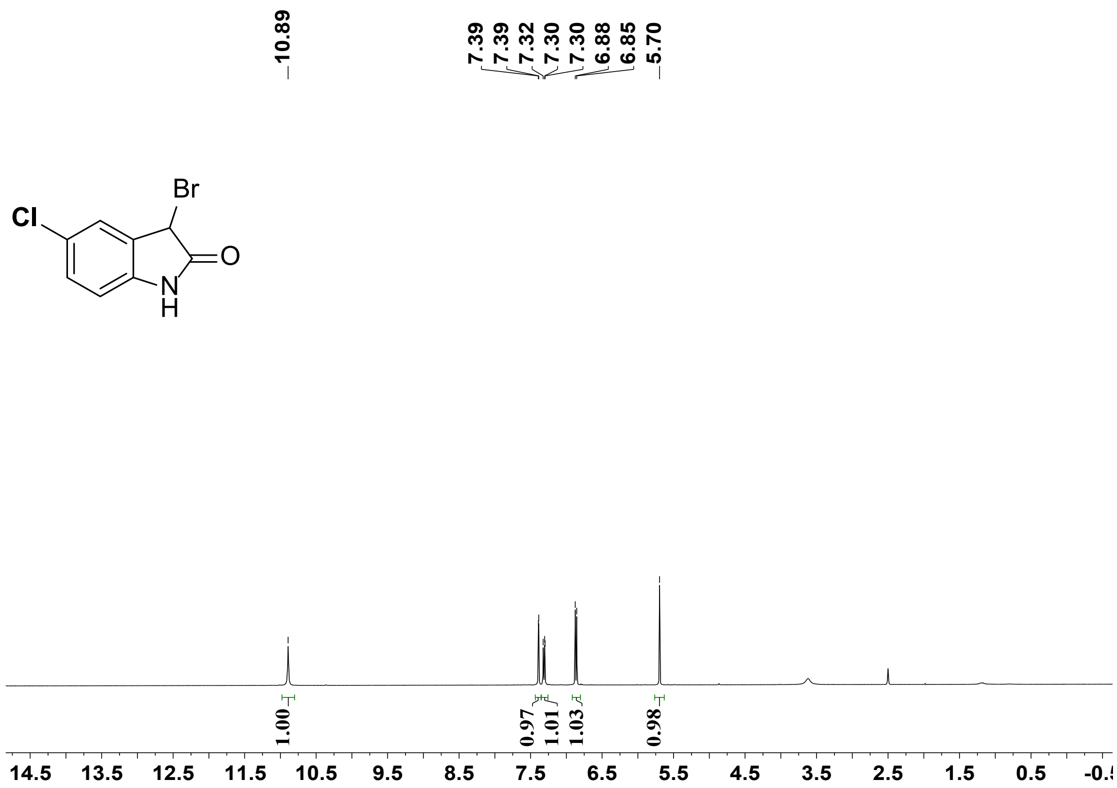

^{1}H NMR spectrum (400 MHz, DMSO-d_6) of **4e**

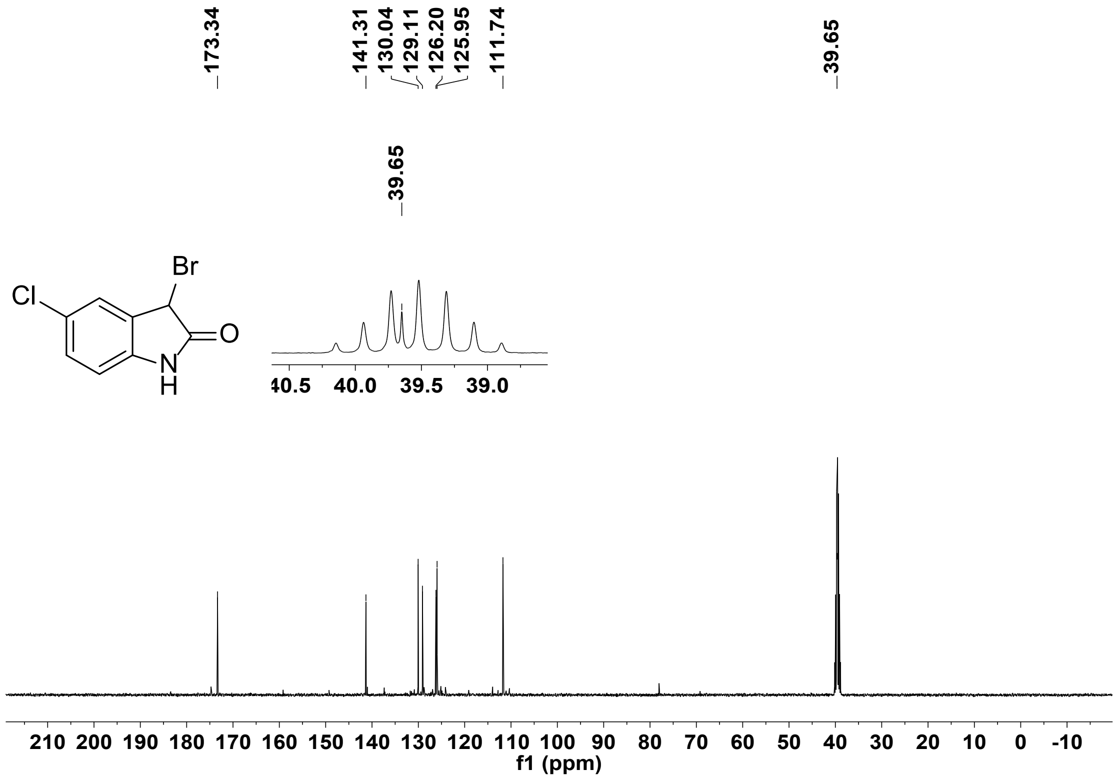

^{13}C NMR spectrum (100 MHz, DMSO-d_6) of **4e**

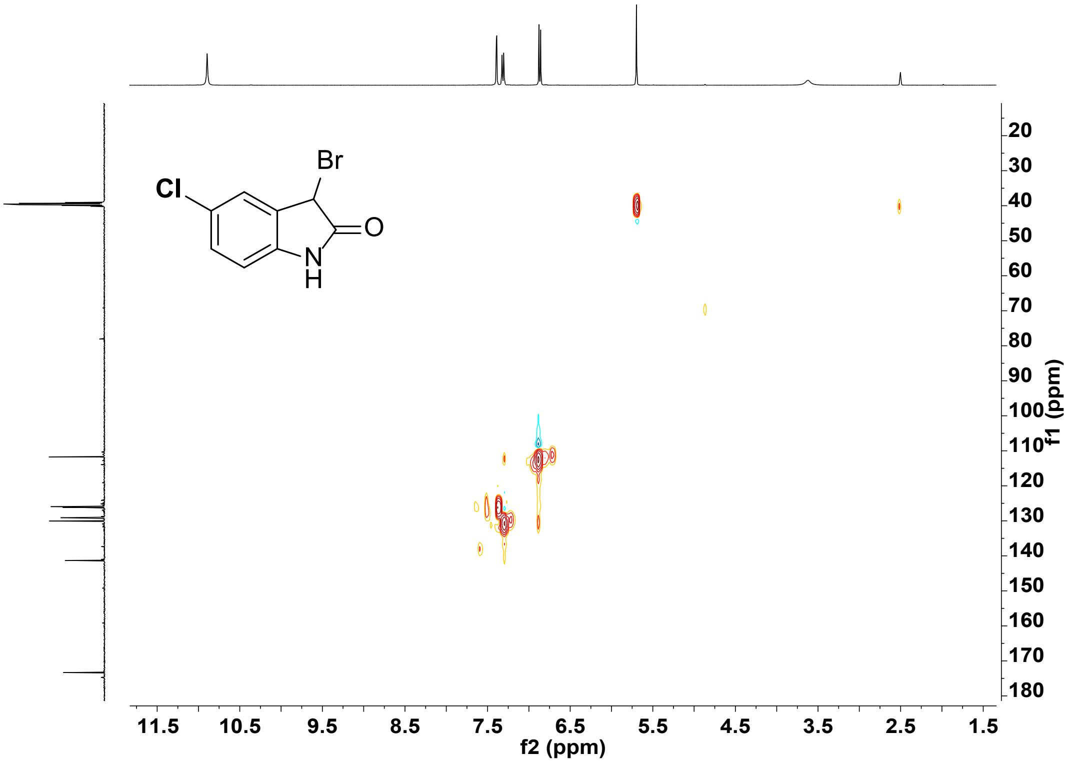

HSQC spectrum of **4e**

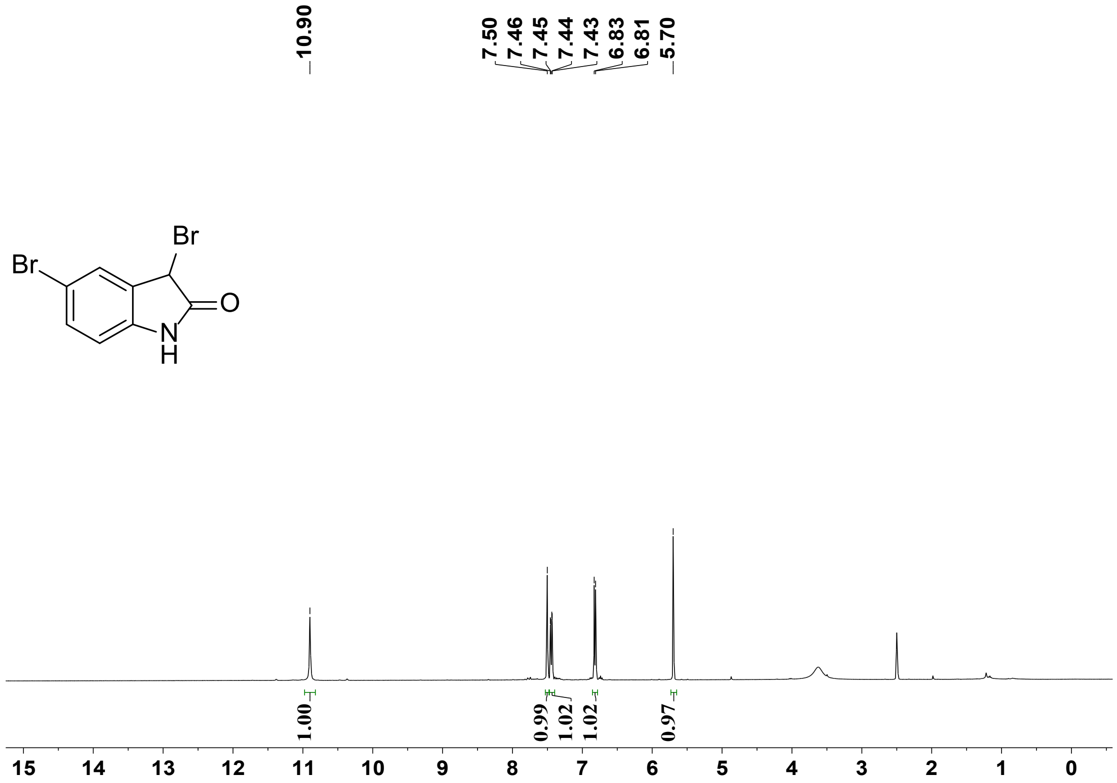

[1]**H NMR** spectrum (400 MHz, DMSO-d_6) of **4f**

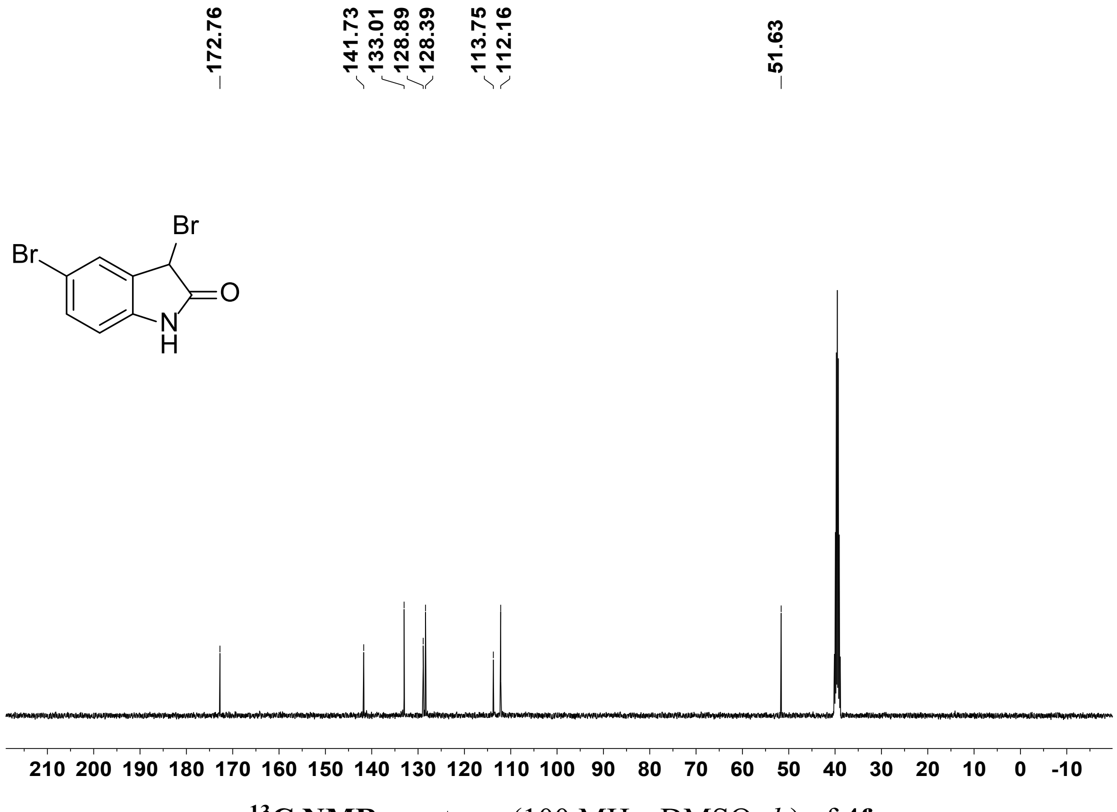

[13]**C NMR** spectrum (100 MHz, DMSO-d_6) of **4f**

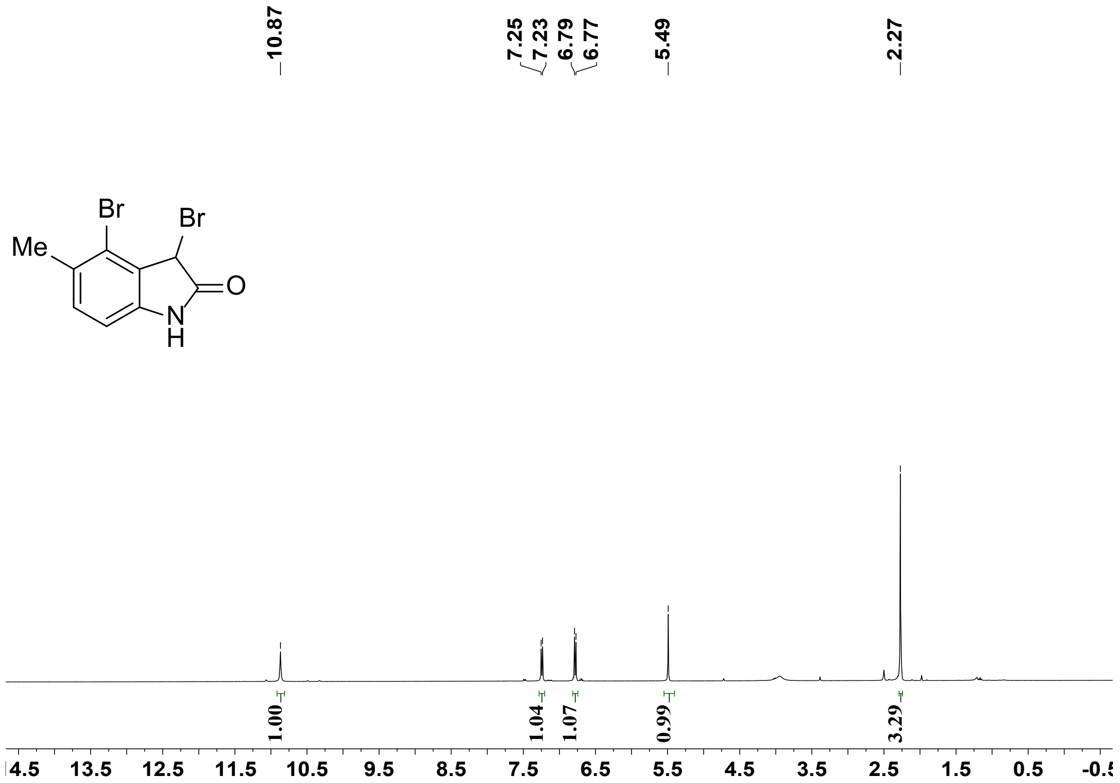

^{1}H NMR spectrum (400 MHz, DMSO-d_6) of **4g**

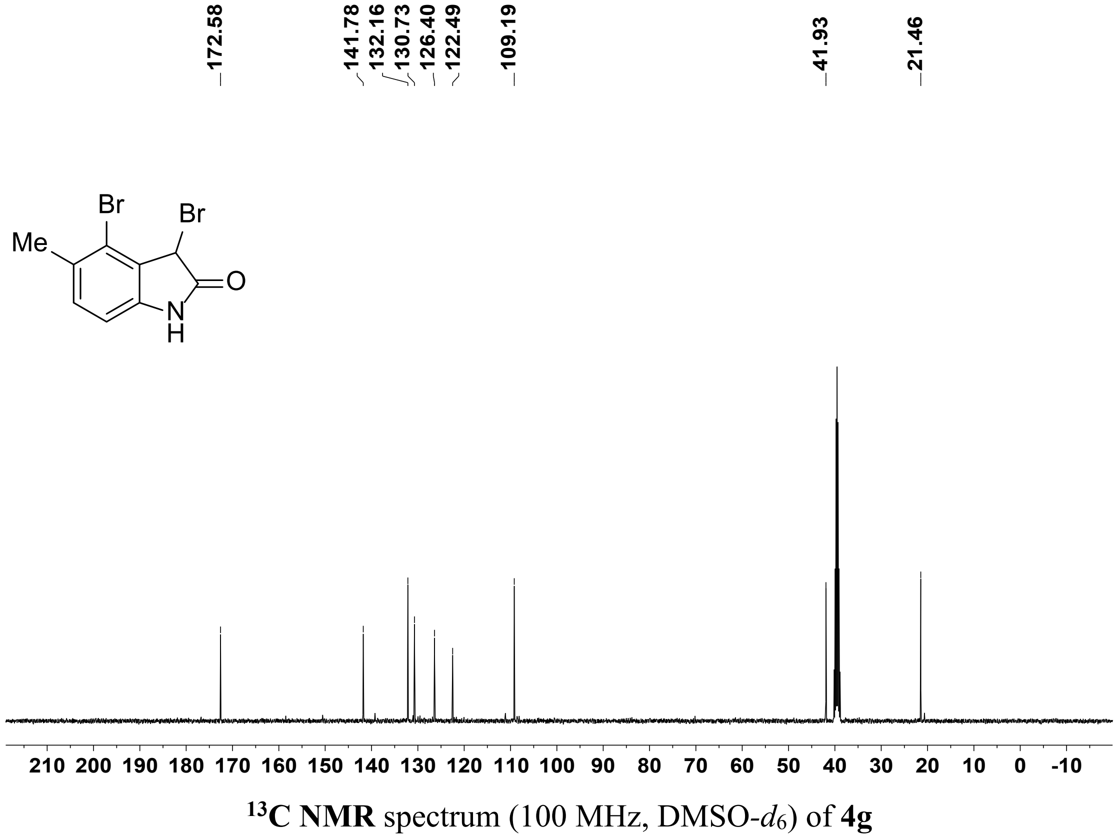

^{13}C NMR spectrum (100 MHz, DMSO-d_6) of **4g**

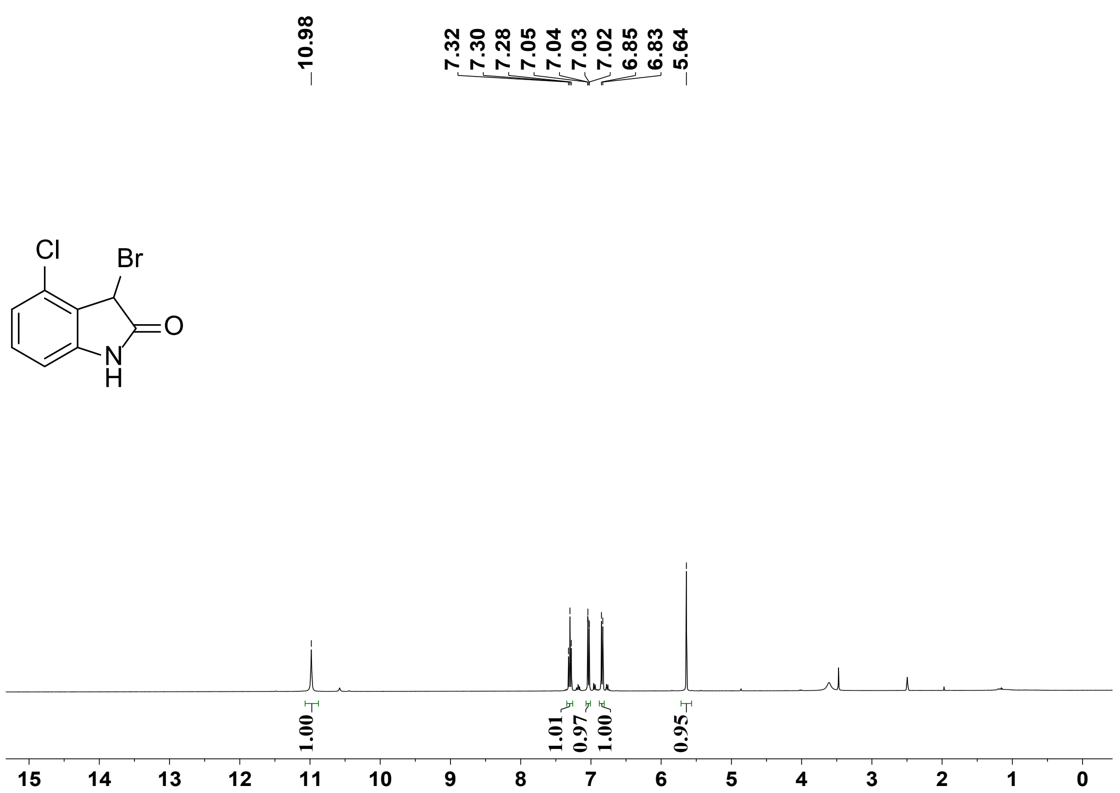

^{1}**H NMR** spectrum (400 MHz, DMSO-d_6) of **4h**

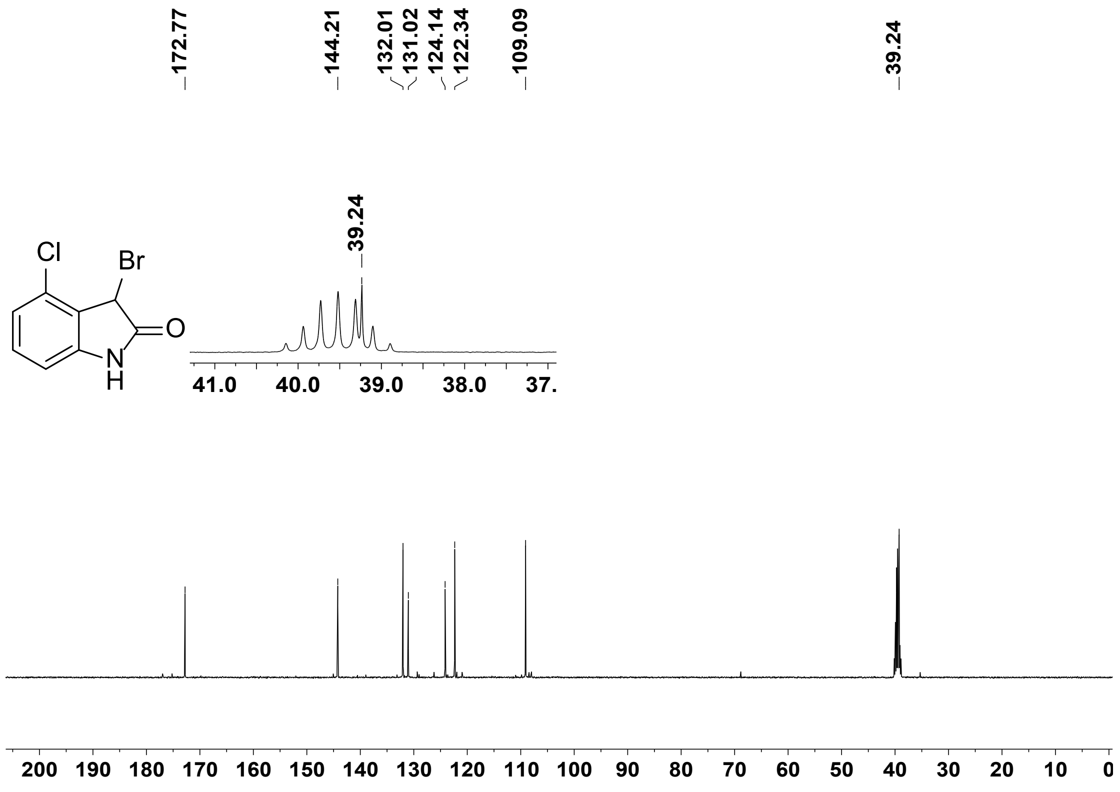

^{13}**C NMR** spectrum (100 MHz, DMSO-d_6) of **4h**

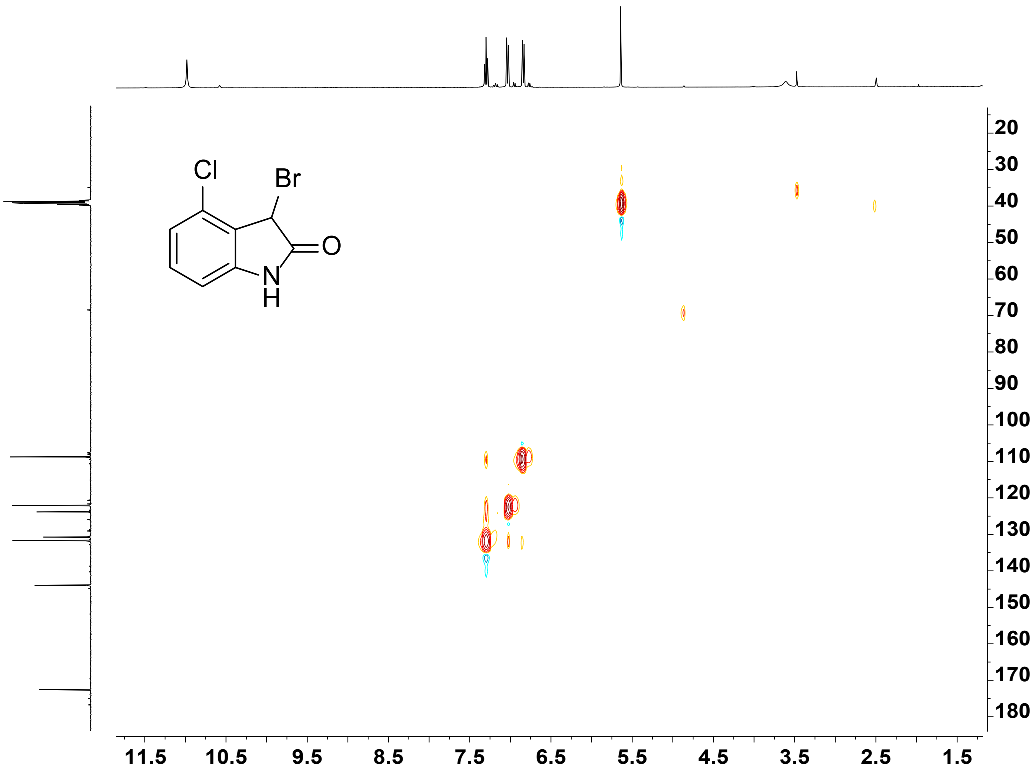

HSQC spectrum of **4i**

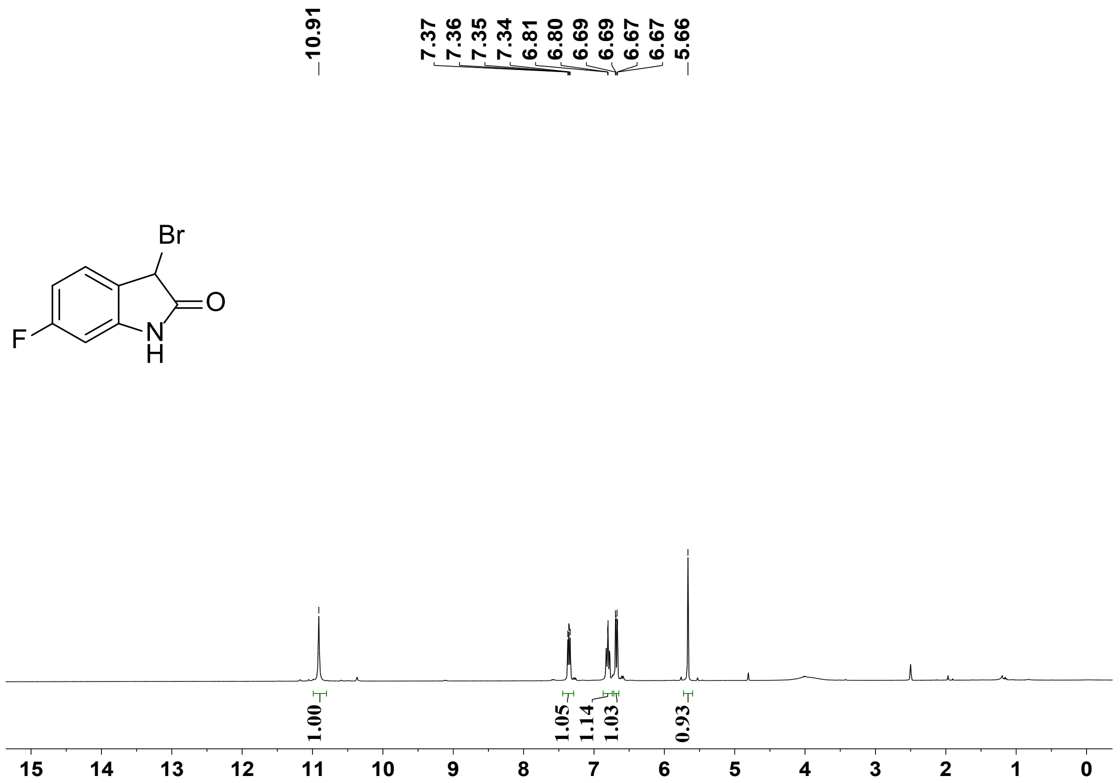

^{1}H NMR spectrum (400 MHz, DMSO-d_6) of **4i**

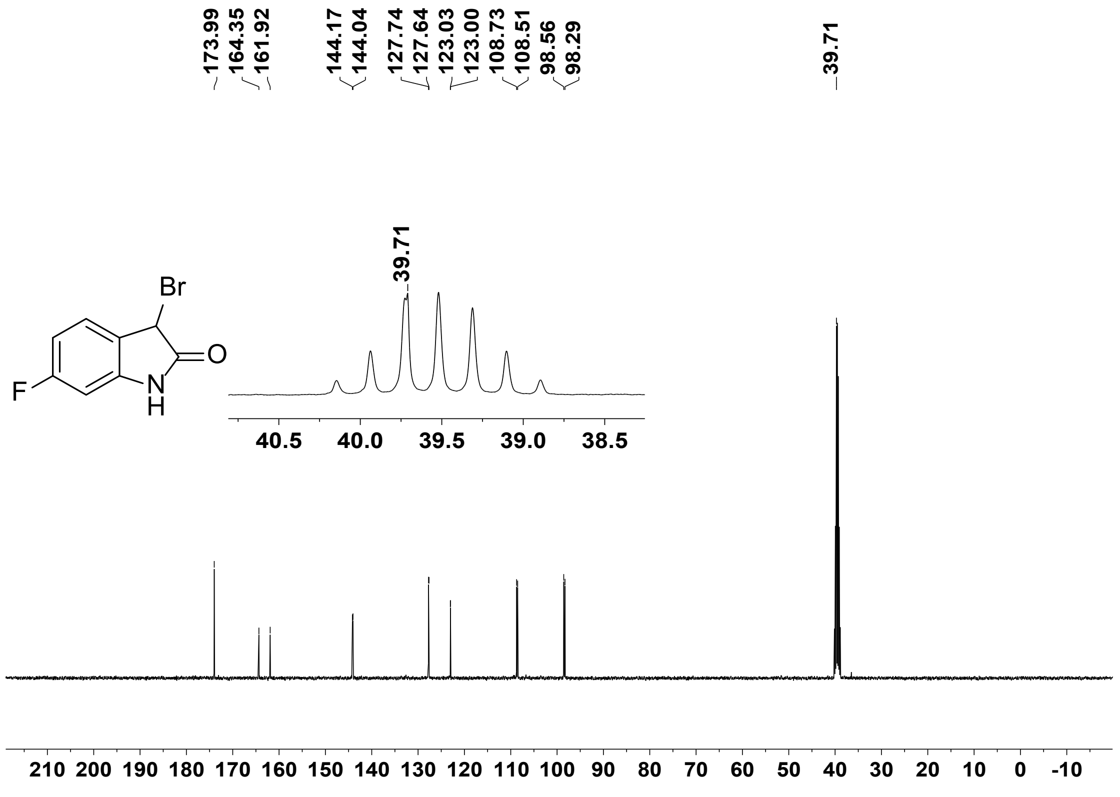

^{13}C NMR spectrum (100 MHz, DMSO-d_6) of **4i**

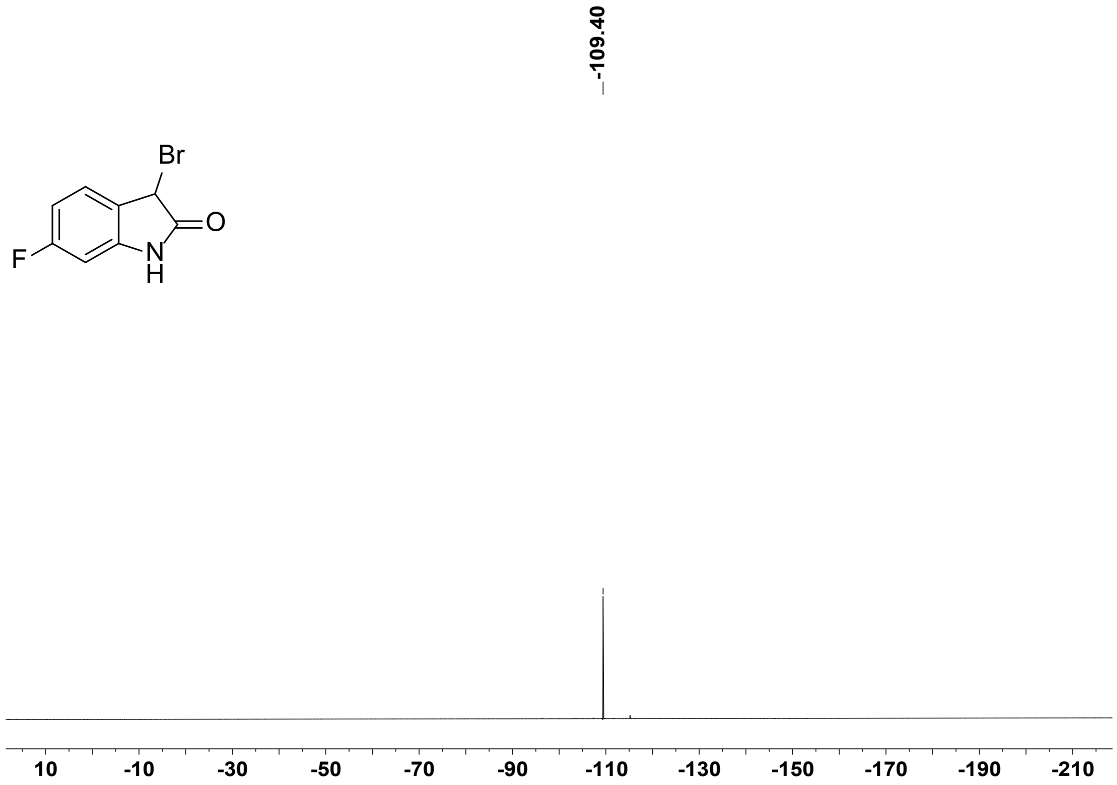

^{19}F NMR spectrum (377 MHz, DMSO-d_6) of **4i**

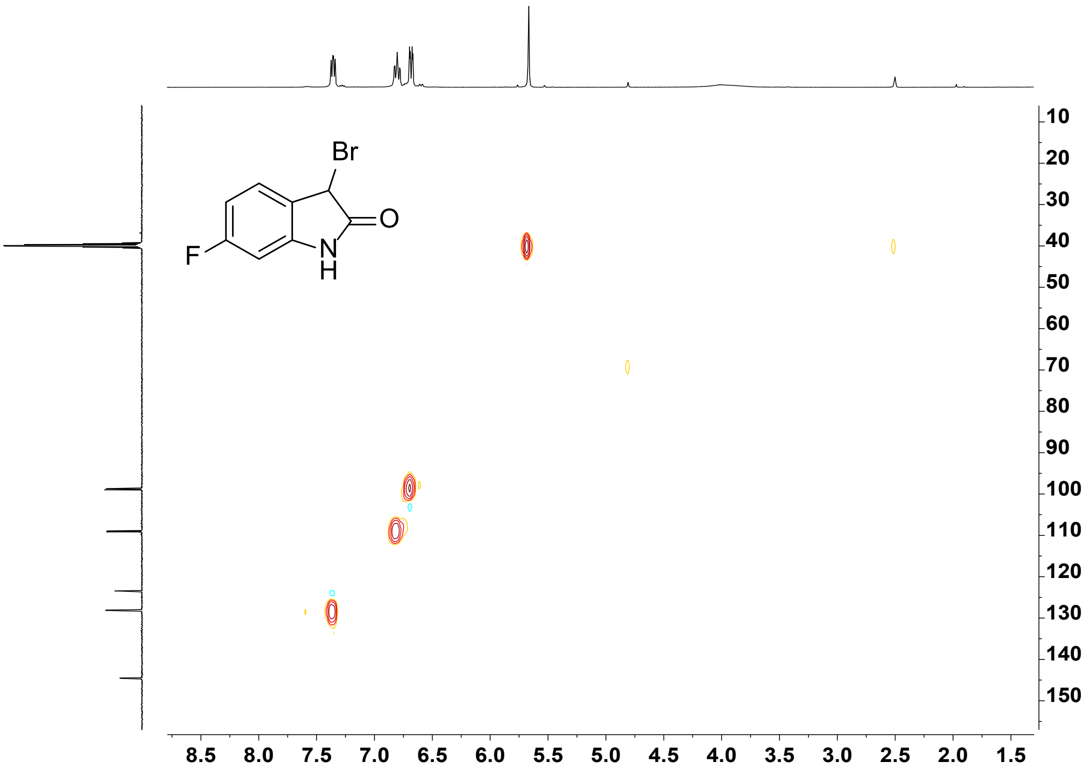

HSQC spectrum of **4i**

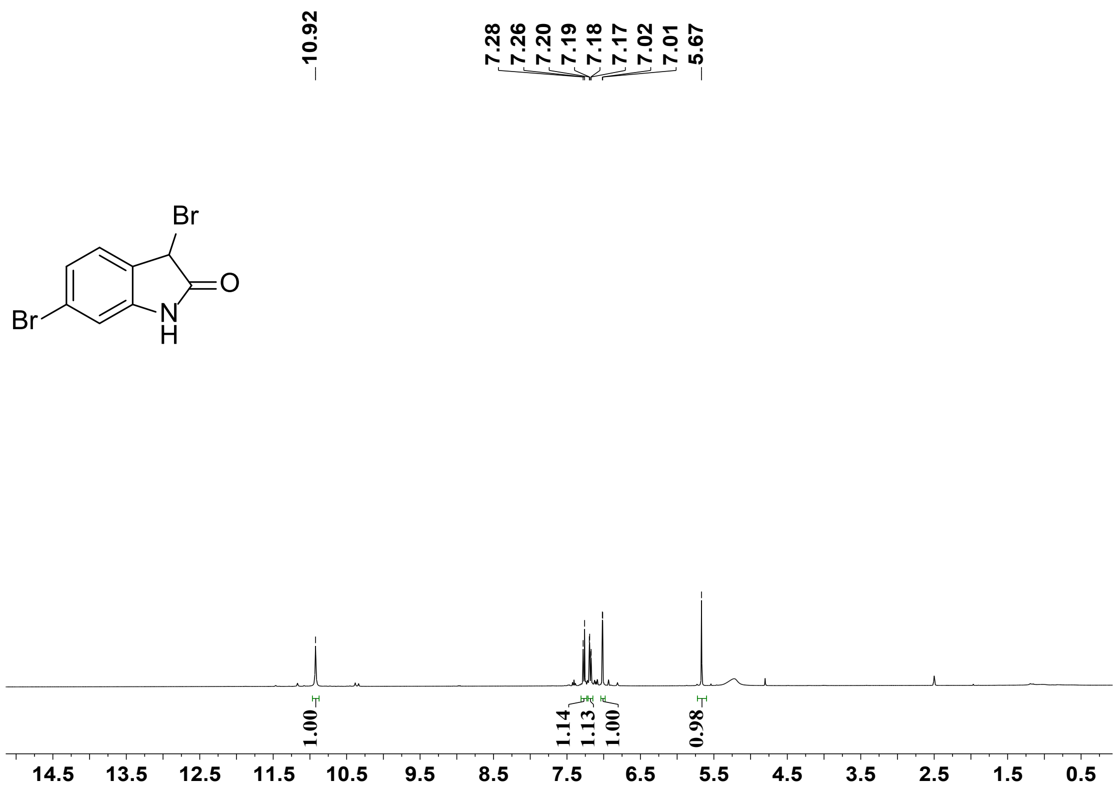

^{1}H NMR spectrum (400 MHz, DMSO-d_6) of **4j**

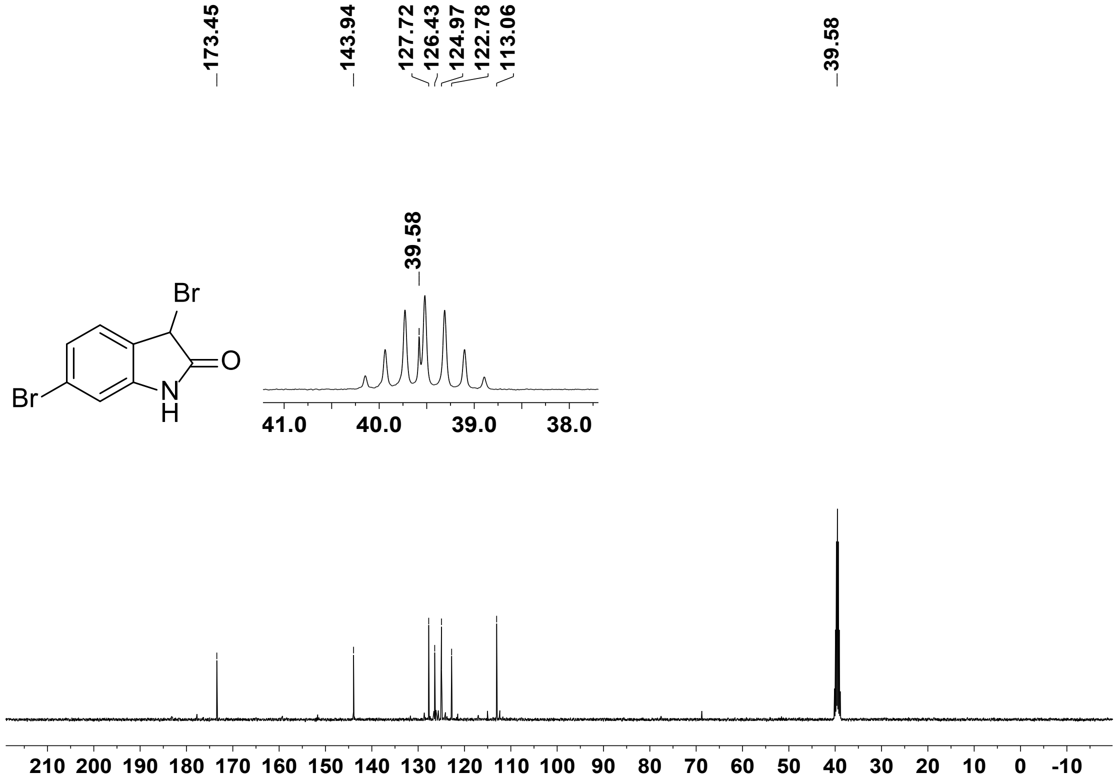

^{13}C NMR spectrum (100 MHz, DMSO-d_6) of **4j**

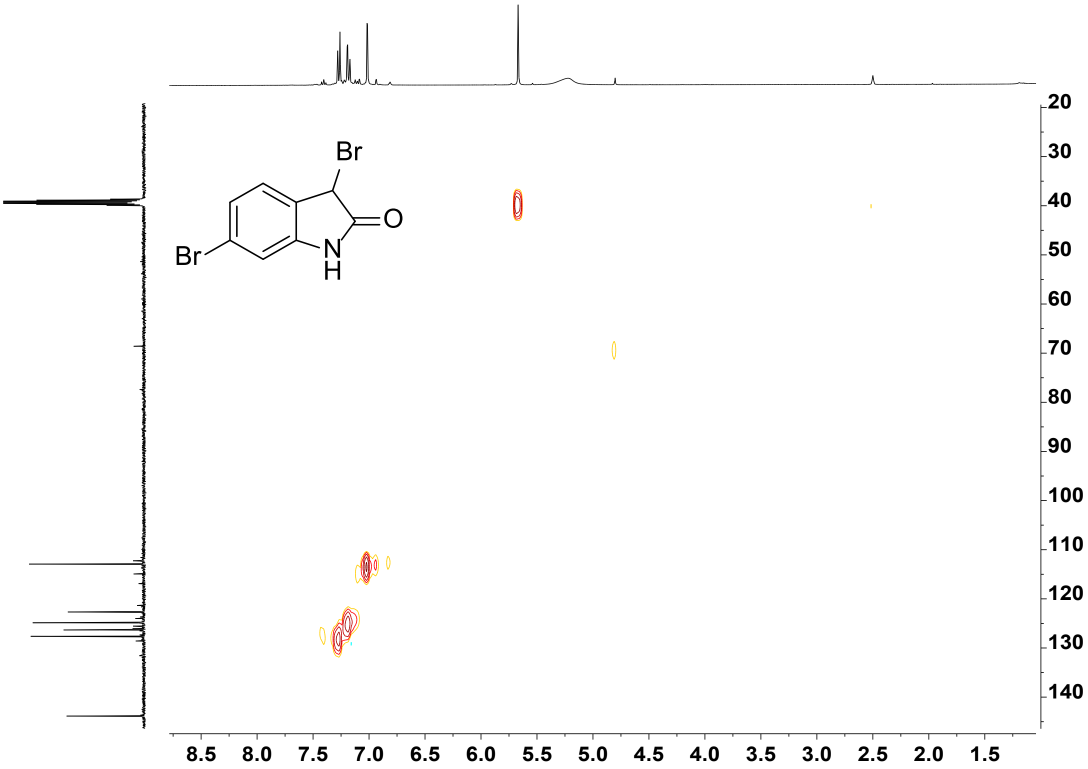

HSQC spectrum of **4j**

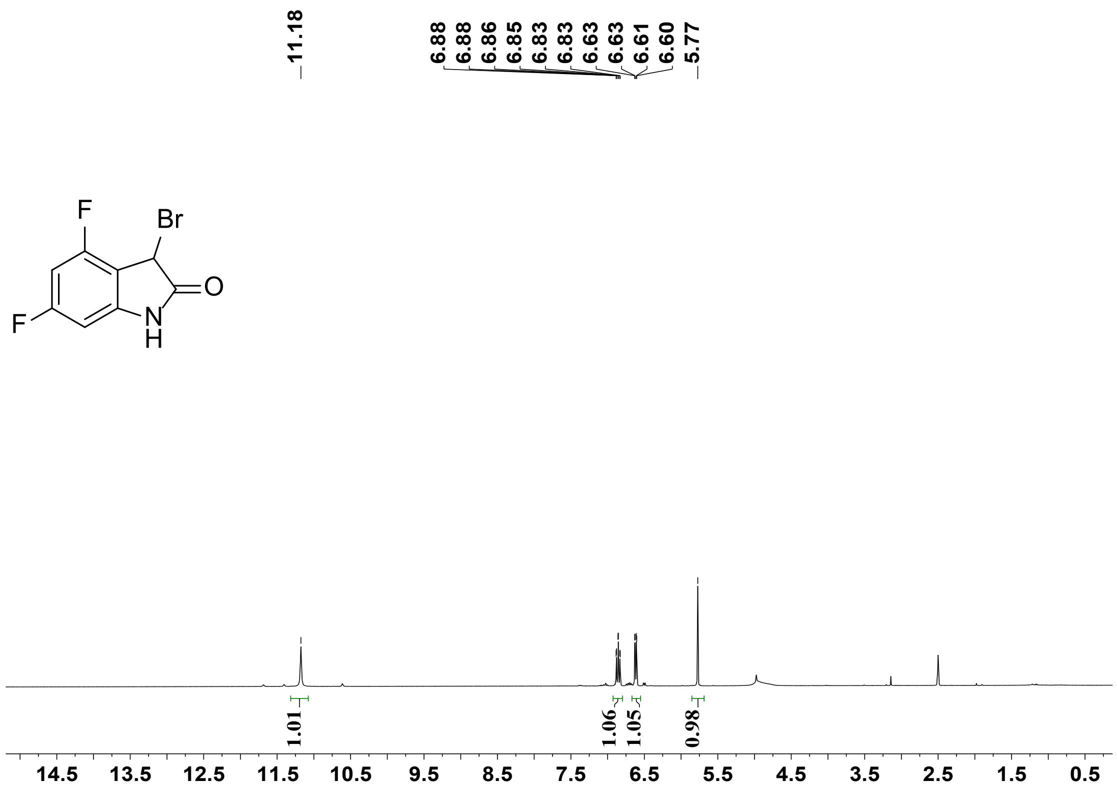

^{1}H NMR spectrum (400 MHz, DMSO-*d*6) of **4k**

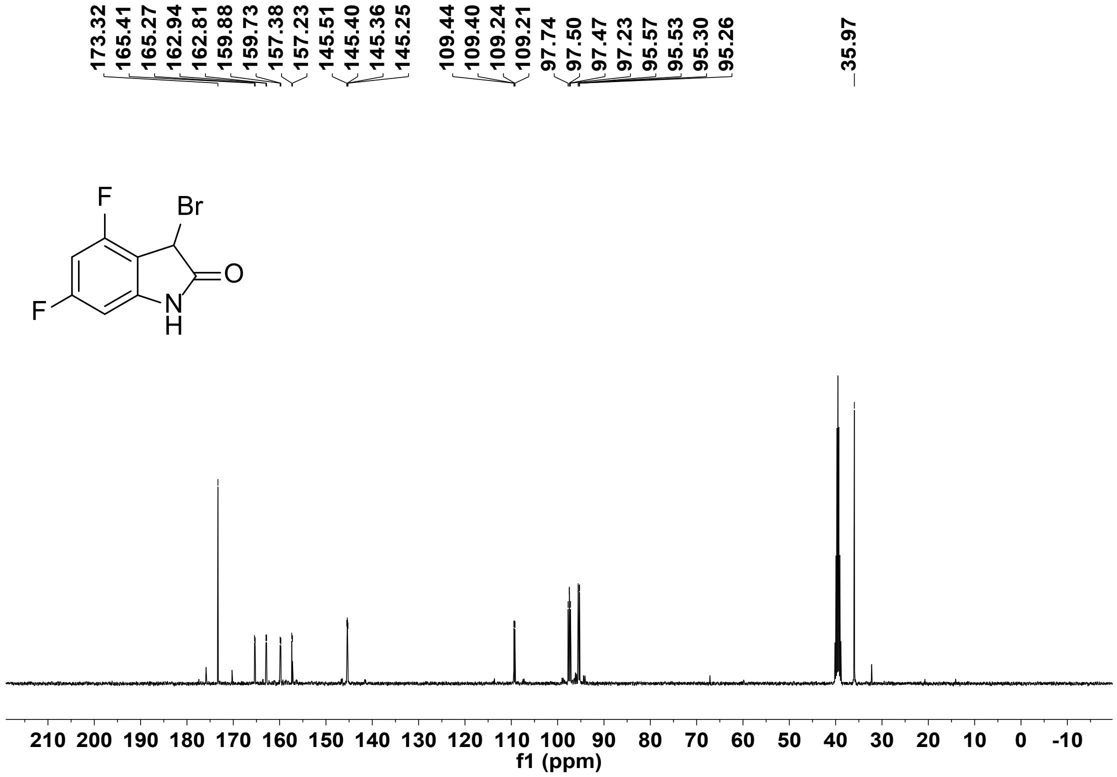

^{13}C NMR spectrum (100 MHz, DMSO-*d*6) of **4k**

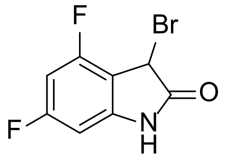

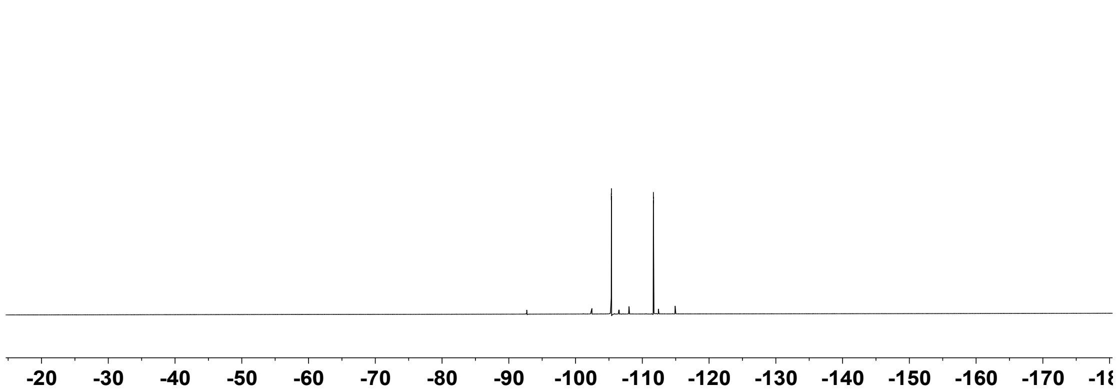

^{19}F NMR spectrum (377 MHz, DMSO-d_6) of **4k**

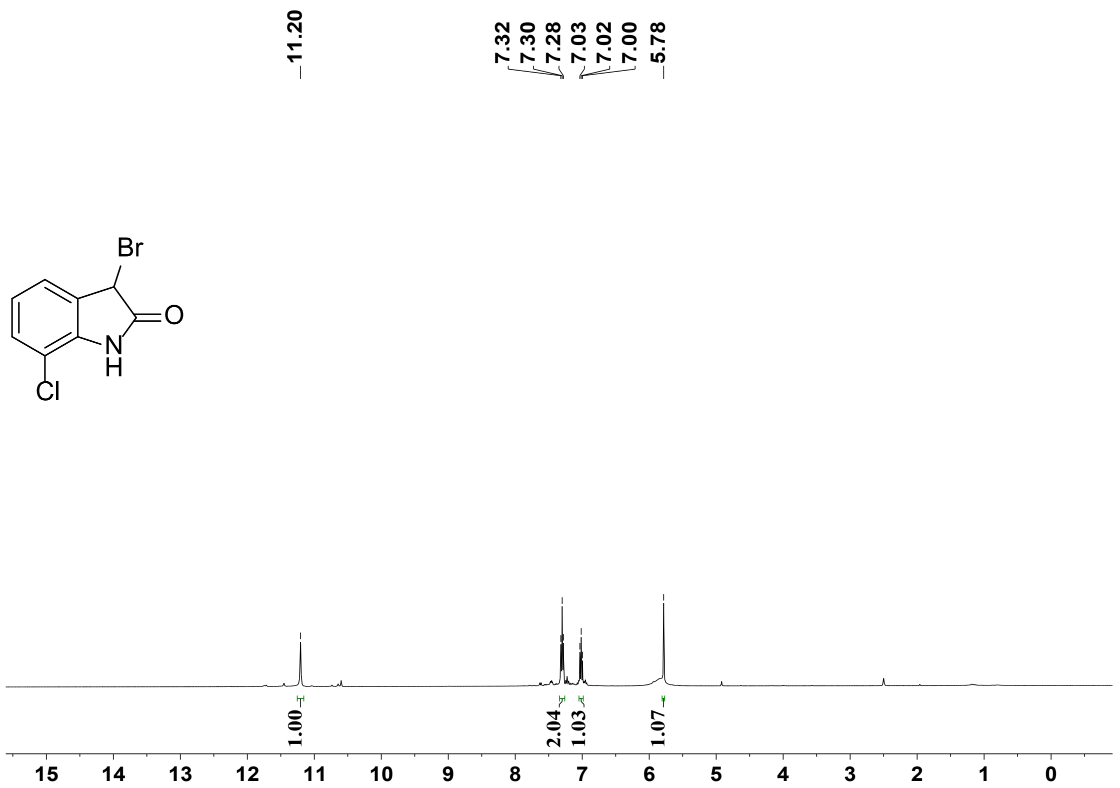

^{1}**H NMR** spectrum (400 MHz, DMSO-d_6) of **4l**

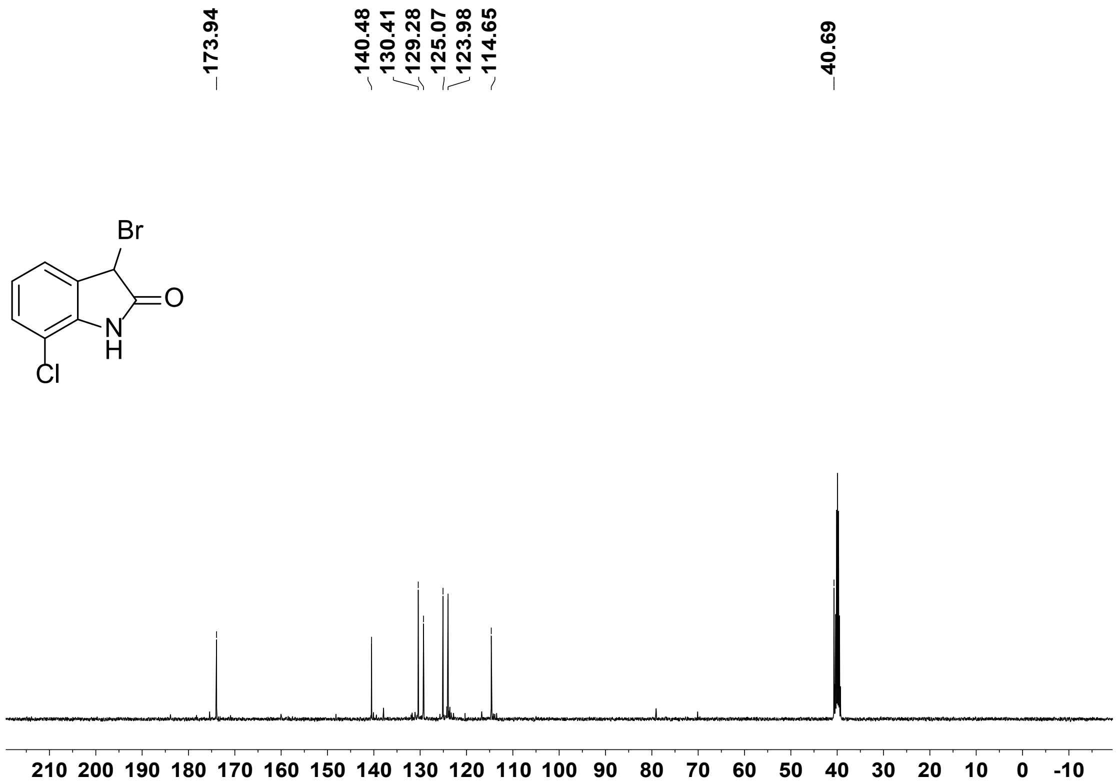

^{13}**C NMR** spectrum (100 MHz, DMSO-d_6) of **4l**

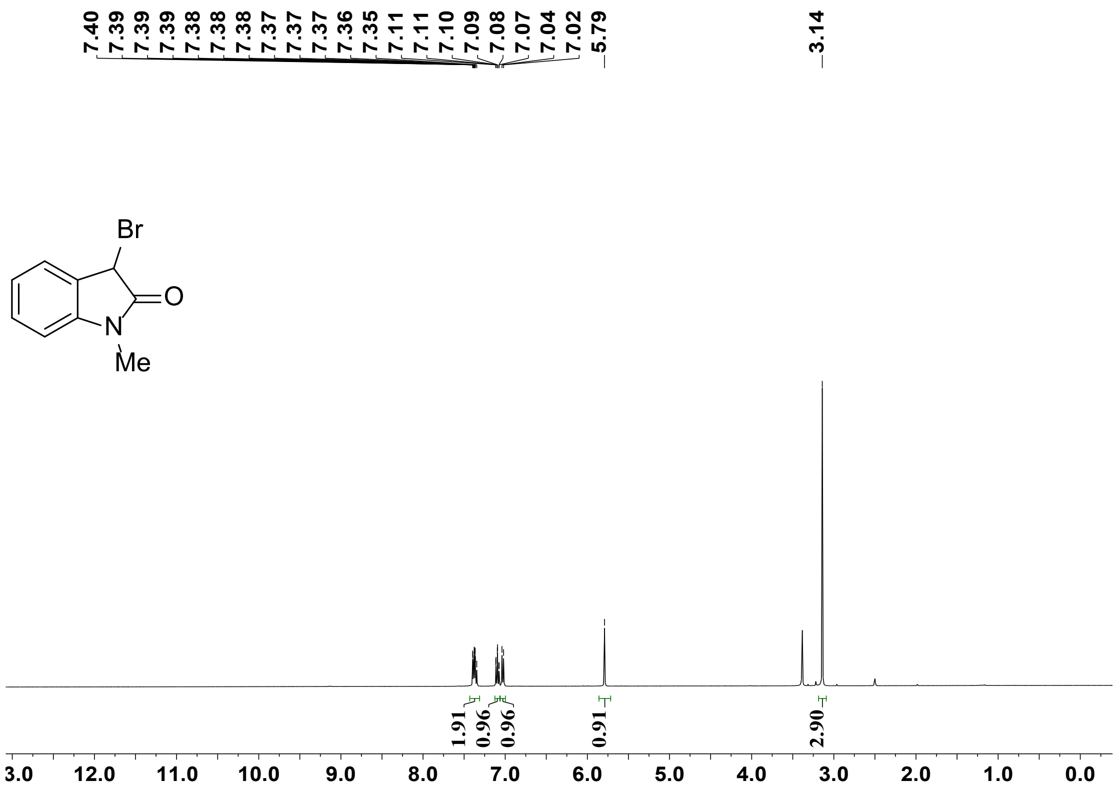

^{1}H NMR spectrum (400 MHz, DMSO-d_6) of **4m**

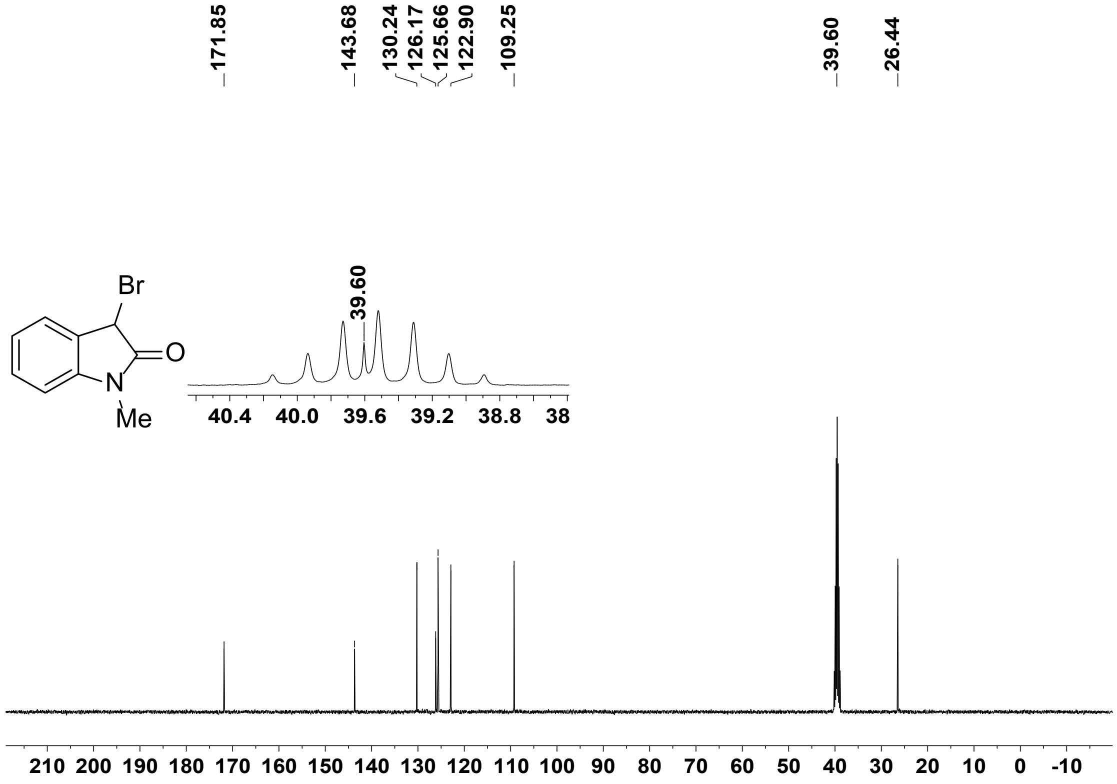

^{13}C NMR spectrum (100 MHz, DMSO-d_6) of **4m**

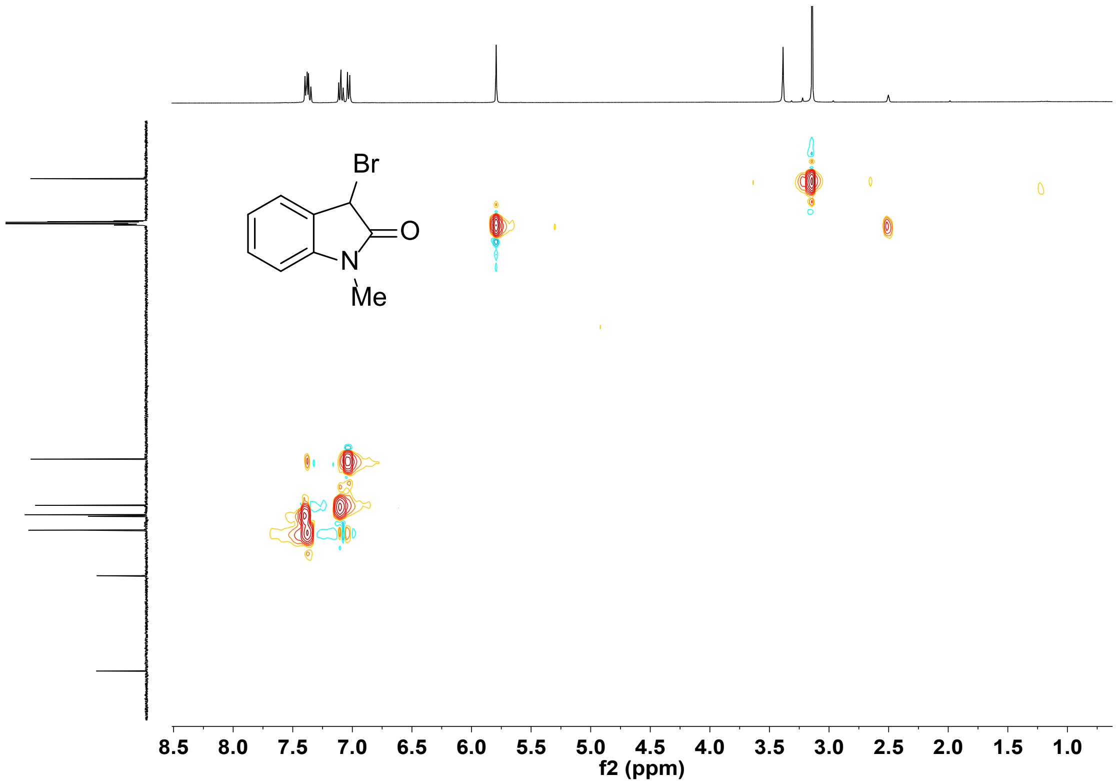

HSQC spectrum of **4m**

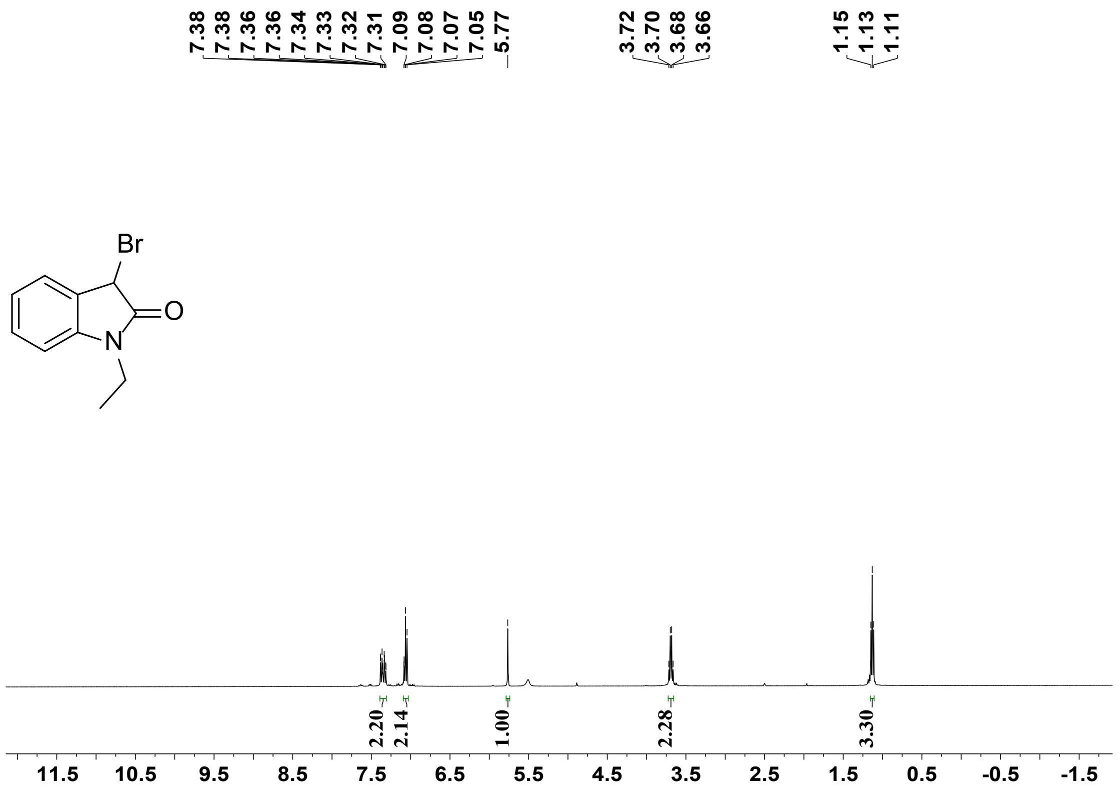

^{1}H NMR spectrum (400 MHz, DMSO-d_6) of **4n**

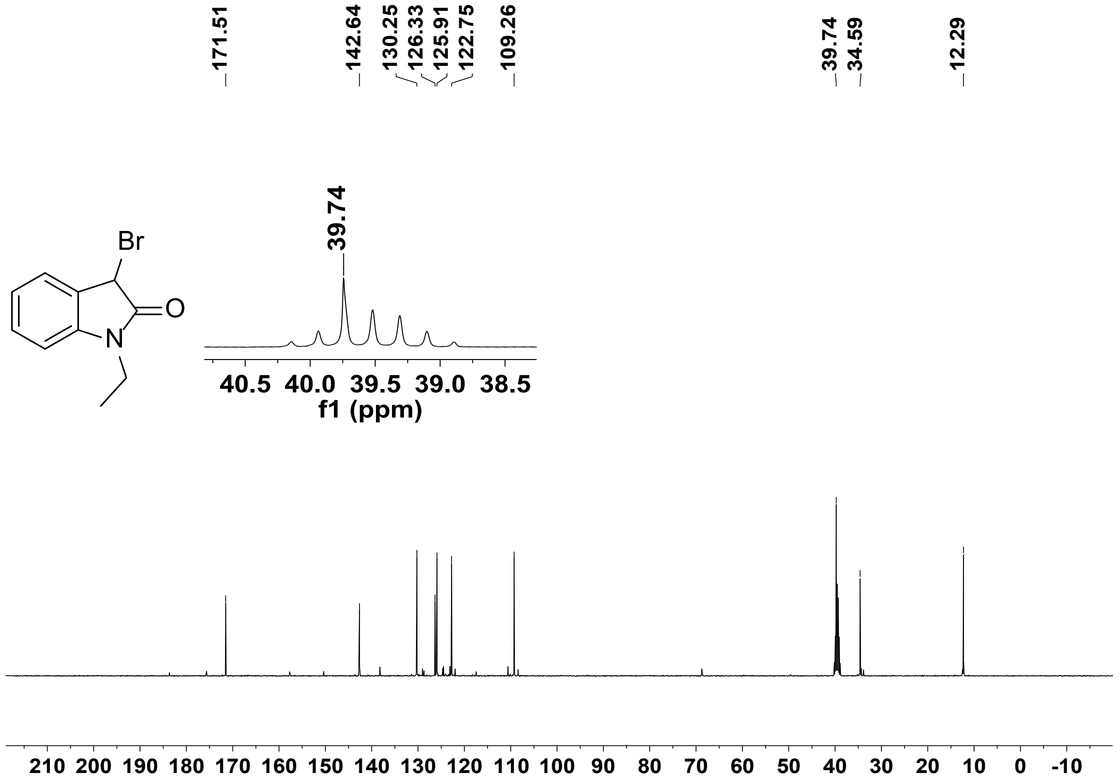

^{13}C NMR spectrum (100 MHz, DMSO-d_6) of **4n**

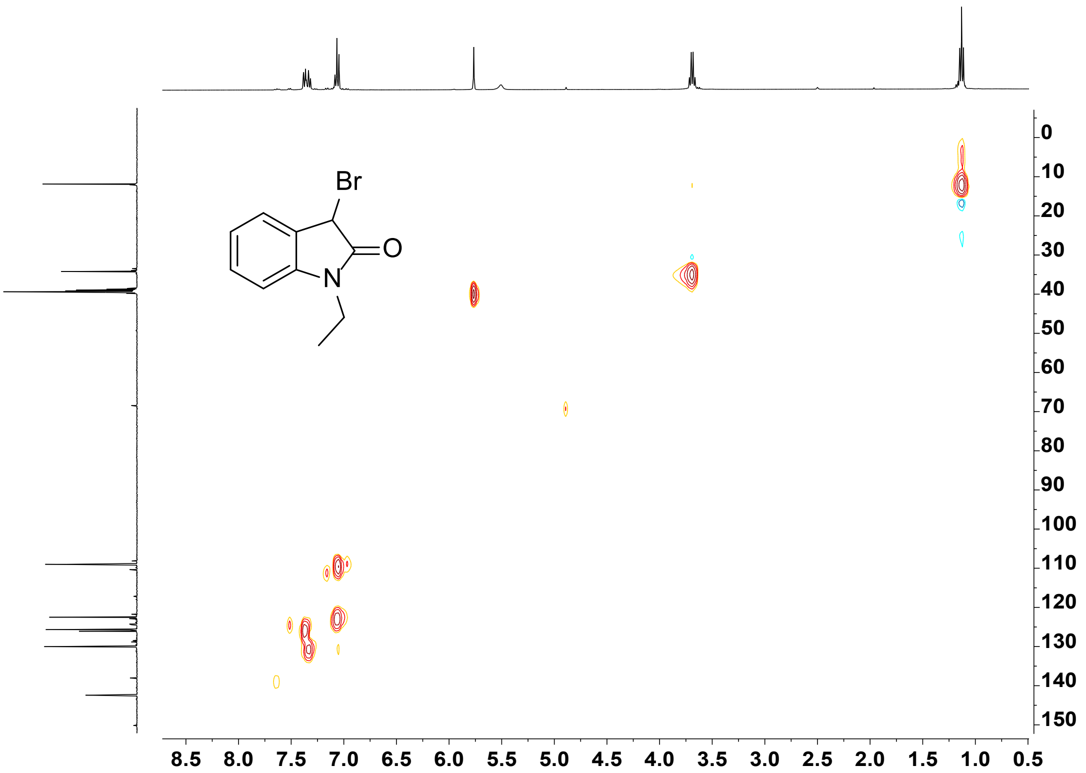

HSQC spectrum of **4n**

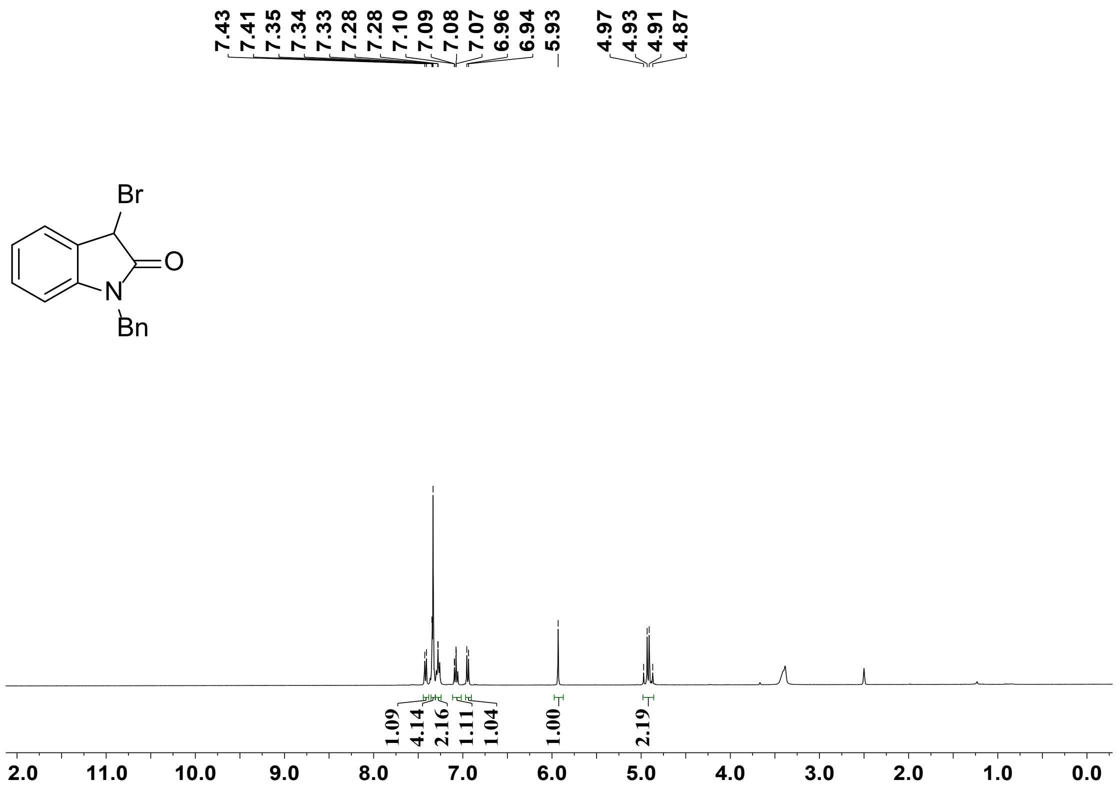

^{1}H NMR spectrum (400 MHz, DMSO-d_6) of **4o**

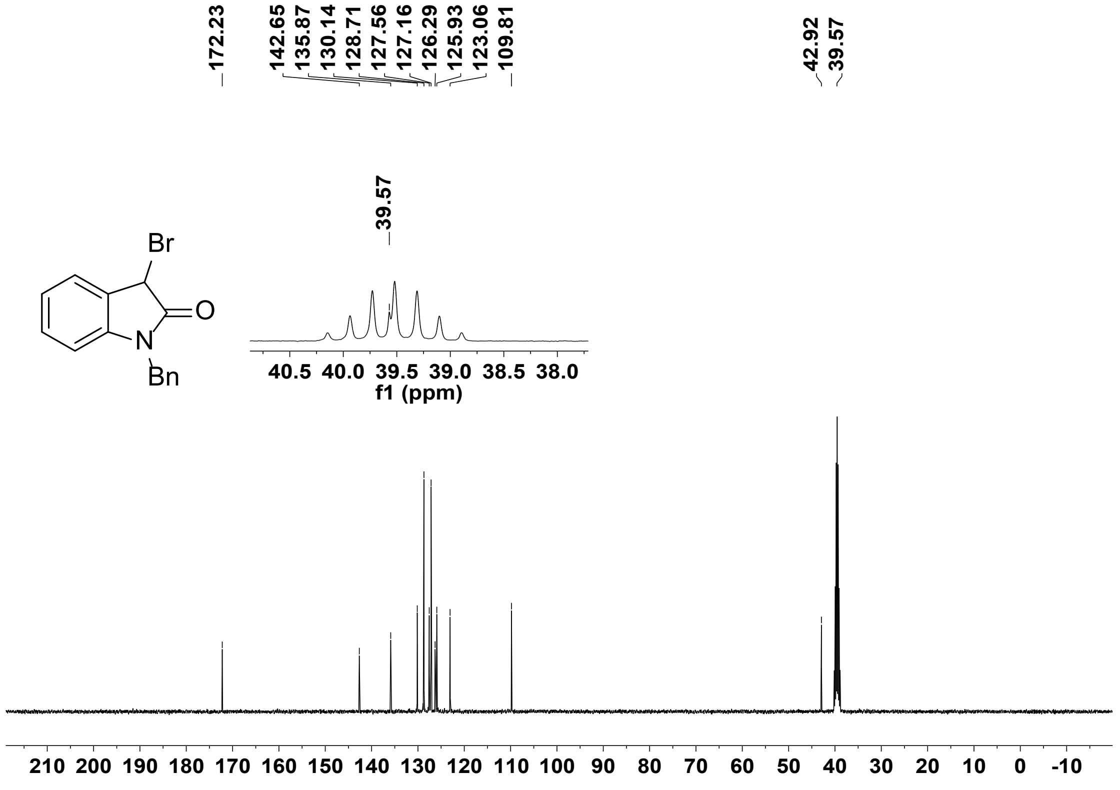

^{13}C NMR spectrum (100 MHz, DMSO-d_6) of **4o**

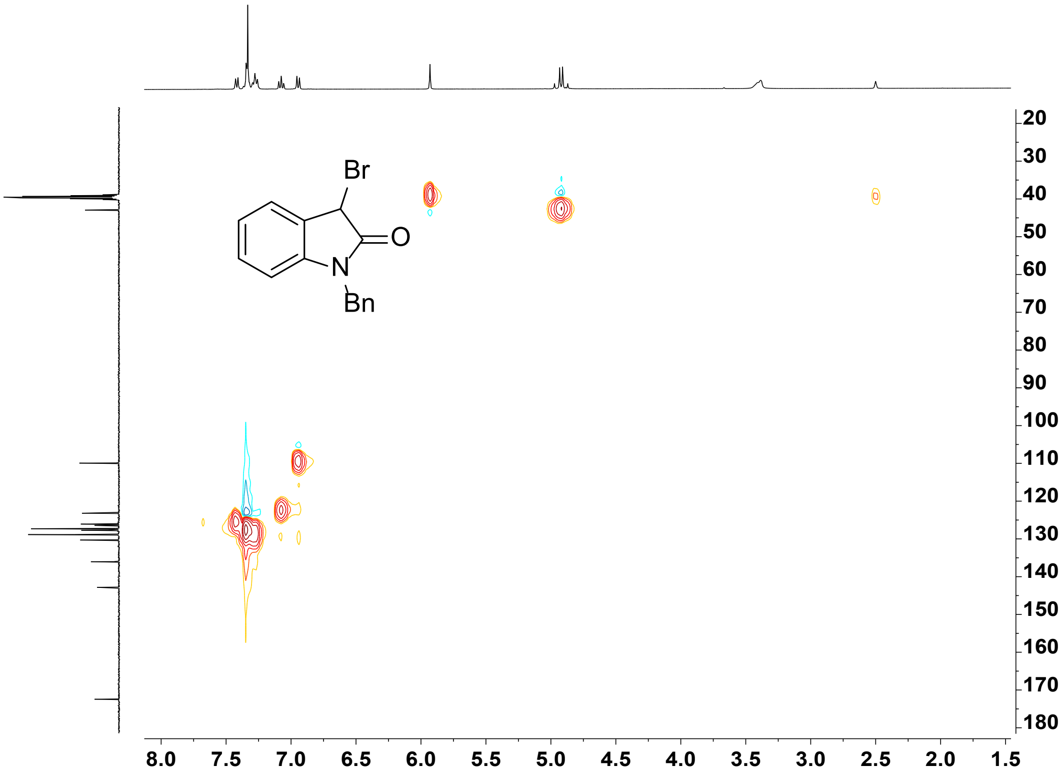

HSQC spectrum of **4o**

References

1 Hayashi, M.; Nakamura, S. *Angew. Chem. Int. Ed. Engl.* **2011,** *50* (10), 2249-2252. doi: 10.1002/anie.201007568

2 El Kaïm, L.; Gaultier, L.; Grimaud, L.; Dos Santos, A. *Synlett* **2005,** *2005* (15), 2335-2336. doi: 10.1055/s-2005-872670

3 Terada, M.; Kondoh, A.; Takei, A. *Synlett* **2016,** *27* (12), 1848-1853. doi: 10.1055/s-0035-1561859

4 Pu, X.; Li, Q.; Lu, Z.; Yang, X. *Eur. J. Org. Chem.* **2016,** *2016* (36), 5937-5940. doi: https://doi.org/10.1002/ejoc.201601226

5 César da Silva, R.; Chatterjee, I.; Escudero-Adán, E.; Weber Paixão, M.; Melchiorre, P. *Asian J. Org. Chem.* **2014,** *3* (4), 466-469. doi: https://doi.org/10.1002/ajoc.201400014

6 Noole, A.; Järving, I.; Werner, F.; Lopp, M.; Malkov, A.; Kanger, T. *Org. Lett.* **2012,** *14* (18), 4922-4925. doi: 10.1021/ol302245b

7 Wang, C.-S.; Li, T.-Z.; Cheng, Y.-C.; Zhou, J.; Mei, G.-J.; Shi, F. *J. Org. Chem.* **2019,** *84* (6), 3214-3222. doi: 10.1021/acs.joc.8b03004

8 Wang, X.; Dong, K.; Yan, B.; Zhang, C.; Qiu, L.; Xu, X. *RSC Advances* **2016,** *6* (74), 70221-70225. doi: 10.1039/C6RA16868J

9 Lakshmi Reddy, V.; Prathima, P. S.; Rao, V. J.; Bikshapathi, R. *New J. Chem.* **2018,** *42* (24), 20152-20155. doi: 10.1039/C8NJ04855J

10 Kajita, H.; Togni, A. *ChemistrySelect* **2017,** *2* (3), 1117-1121. doi: https://doi.org/10.1002/slct.201700024

11 Marek, L.; Kolman, L.; Váňa, J.; Svoboda, J.; Hanusek, J. *Beilstein Journal of Organic Chemistry* **2021,** *17,* 527-539. doi: 10.3762/bjoc.17.47
